# Emerging Neural Recording and Neurostimulation Technologies Based on Brain–Computer Interface: A Promising Approach for Neuropsychiatric Disorders

**DOI:** 10.1002/mco2.70739

**Published:** 2026-04-13

**Authors:** Yeguang Xu, Danyang Chen, Qing Ye, Peng Zhang, Jian Shi, Shengjie Li, Yuhao Sun, Zhixian Zhao, Yingxin Tang, Ping Zhang, Zhouping Tang

**Affiliations:** ^1^ Department of Neurology Tongji Hospital Tongji Medical College Huazhong University of Science and Technology Wuhan Hubei China; ^2^ Brain‐Computer Interface Research Institute Tongji Hospital Tongji Medical College of Huazhong University of Science and Technology Wuhan Hubei China; ^3^ Tongji Hospital Tongji Medical College Huazhong University of Science and Technology Wuhan Hubei China; ^4^ Hubei Bioinformatics and Molecular Imaging Key Laboratory Department of Biomedical Engineering College of Life Science and Technology Huazhong University of Science and Technology Wuhan Hubei China; ^5^ School of Mechanical Science and Engineering Huazhong University of Science and Technology Wuhan Hubei China

**Keywords:** brain–computer interface, closed‐loop stimulation, deep brain stimulation, endovascular electrode arrays, epidural spinal cord stimulation, neuropsychiatric disorders

## Abstract

Neurological and psychiatric disorders, arising from disruptions in neural circuitry, pose a major and growing challenge to global healthcare systems. Brain–computer interface (BCI) technology has emerged as a promising approach, enabling direct communication between the brain and external devices. By facilitating bidirectional interaction with the nervous system, BCIs open new avenues for both diagnosis and treatment. In this review, we examine recent advances in recording and stimulation technologies within the BCI framework and evaluate their therapeutic potential across major neuropsychiatric disorders. We focus particularly on post‐stroke motor rehabilitation as a representative paradigm, providing detailed analysis of the mechanisms, clinical evidence, and future prospects of endovascular BCI, BCI‐integrated epidural spinal cord stimulation, and BCI‐driven deep brain stimulation. We further extend the discussion to movement disorders such as Parkinson's disease and epilepsy, as well as cognitive and psychiatric conditions including Alzheimer's disease and depression, highlighting how BCI‐based approaches enable symptom detection and closed‐loop neuromodulation. Additionally, we address ethical and societal considerations accompanying clinical translation of these advanced neurotechnologies. By integrating current evidence, this review highlights a paradigm shift toward more active, precise, and personalized neural rehabilitation enabled by BCI systems, while outlining key challenges and future directions for research and clinical application.

## Graphical abstract

This review explores brain–computer interface (BCI)–based neural recording and stimulation for treating neuropsychiatric disorders. It details three emerging technologies: endovascular BCI for minimally invasive signal acquisition, BCI‐integrated epidural spinal cord stimulation for motor restoration, and closed‐loop deep brain stimulation for adaptive neuromodulation. Focusing on post‐stroke recovery, it extends the application to Parkinson's disease, epilepsy, Alzheimer's disease, and depression. The article analyzes their mechanisms, preclinical/clinical evidence, and technical challenges, highlighting the paradigm shift toward active, precise neural rehabilitation and discussing the ethical and translational pathway for future clinical impact.

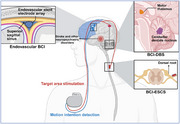

## 1 Introduction

Neurological and psychiatric disorders are among the leading causes of disability and diminished quality of life worldwide [1]. These conditions encompass a wide spectrum of clinical manifestations: some primarily impair motor function or cause seizures, while others lead to emotional disturbances and cognitive decline [2, 3]. Although conventional treatments, including pharmacotherapy, physical rehabilitation, and psychotherapy, provide benefit for many patients, they are often limited by plateauing efficacy, adverse effects, and substantial interindividual variability in treatment response [4, 5, 6]. For example, in post‐stroke motor rehabilitation, training‐based interventions can promote neuroplasticity, but functional recovery frequently plateaus within 6 months, particularly in patients with severe deficits or in the chronic phase [7, 8]. In Parkinson's disease (PD), long‐term levodopa therapy is commonly associated with motor complications [9]. Similarly, a significant proportion of patients with drug‐resistant epilepsy (DRE) or treatment‐resistant depression fail to achieve adequate symptom control with existing therapies [10, 11]. Despite their heterogeneity, many neuropsychiatric disorders share a common underlying feature: an imbalance in excitatory and inhibitory signaling or disrupted functional connectivity within specific neural circuits [12, 13]. This shared pathophysiology has driven growing interest in interventional technologies capable of directly interfacing with the nervous system, simultaneously recording neural activity and delivering targeted neuromodulation. In recent years, brain–computer interface (BCI) technology has emerged as a transformative paradigm with the potential to overcome longstanding limitations in current therapeutic approaches.

BCI systems enable real‐time recording and decoding of neural activity, transforming brain signals into control commands for external devices while simultaneously delivering sensory feedback, including motor, tactile, and visual inputs. Through this bidirectional communication, BCIs have the capacity to augment intrinsic neural function and provide meaningful therapeutic and rehabilitative benefits for patients with neurological and psychiatric disorders [14, 15]. A typical BCI system operates through several integrated stages: signal acquisition and preprocessing, feature extraction and decoding, device control, and feedback delivery [16]. Early research in the BCI field focused primarily on assistive communication and control technologies for individuals with severe motor impairments, particularly those associated with conditions such as amyotrophic lateral sclerosis (ALS) and stroke [15]. However, BCI applications have expanded rapidly into neurorehabilitation and psychiatric domains. In rehabilitation, BCIs, often combined with functional electrical stimulation, robotic devices, or virtual reality, are designed to promote use‐dependent neuroplasticity and restore lost function [17]. Moreover, advances in neuromodulation have enabled the development of closed‐loop BCI systems, in which therapeutic interventions such as deep brain stimulation (DBS) are dynamically adjusted based on real‐time detection of pathological neural biomarkers. Examples include β‐band oscillations in PD and epileptiform activity in epilepsy, allowing for more precise and adaptive treatment strategies [18, 19].

**FIGURE 1:**
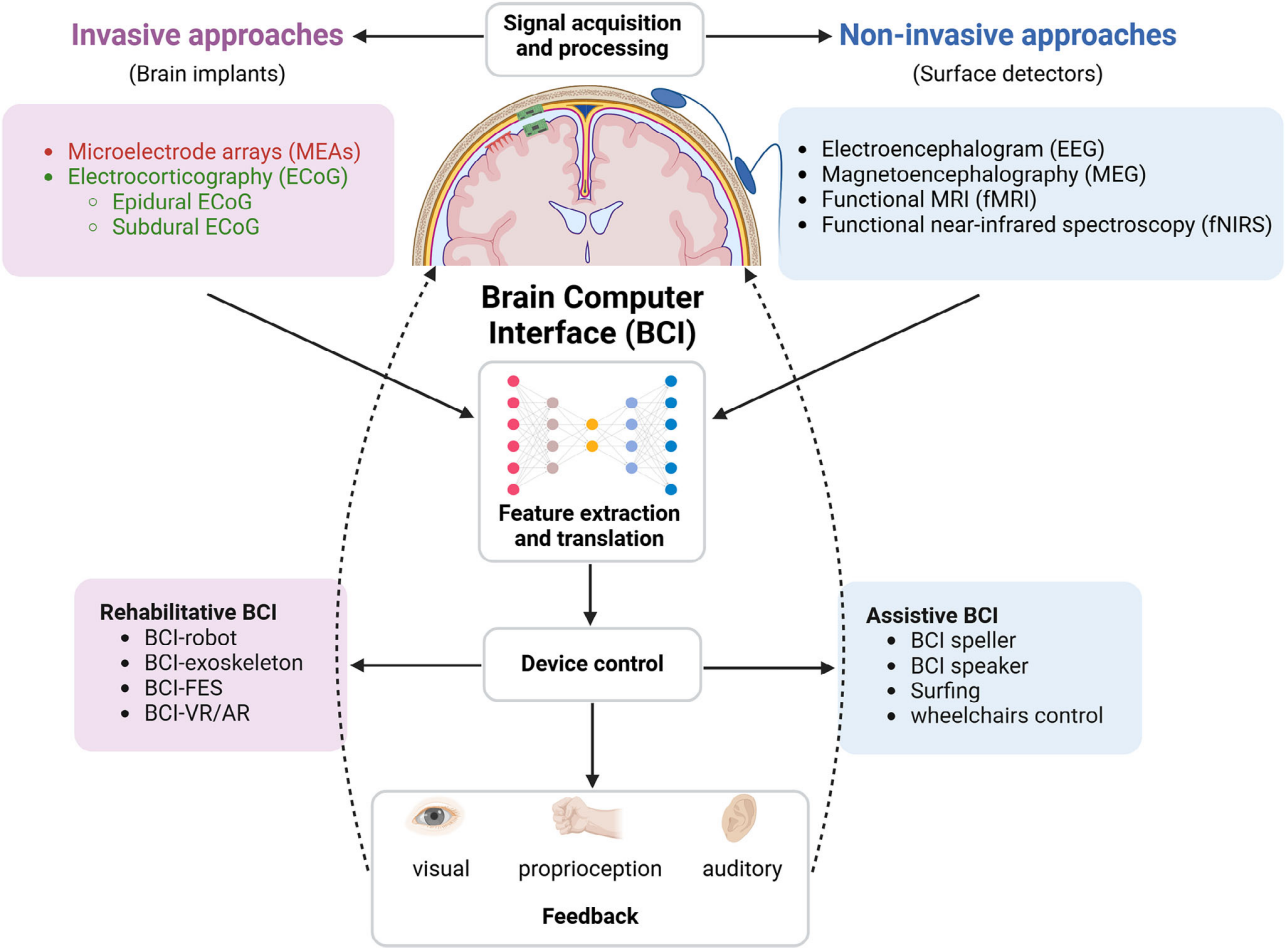
An overview of BCI. The typical BCI system framework is presented, with a particular focus on the invasive and non‐invasive methods of signal acquisition, as well as the design of rehabilitative and assistive BCI systems tailored for stroke patients. BCI, brain–computer interface; FES, functional electrical stimulation; VR/AR, virtual reality/augmented reality. This figure was created using BioRender.com.

Based on the mode of neural signal acquisition, BCI systems are broadly classified into non‐invasive and invasive approaches (Figure 1; see Table 1 for a detailed comparison) [20, 21, 22, 23]. Non‐invasive methods rely on neuroimaging and electrophysiological techniques such as electroencephalography (EEG), functional magnetic resonance imaging (fMRI), magnetoencephalography (MEG), and functional near‐infrared spectroscopy (fNIRS) [24]. While these approaches are attractive due to their safety and accessibility, their clinical utility is often constrained by limited spatial resolution, signal fidelity, and susceptibility to noise [25, 26, 27]. In contrast, invasive BCI systems, particularly those based on microelectrode arrays, enable direct recording from targeted cortical regions, offering superior spatiotemporal resolution and higher information transfer rates [28]. These systems are typically implemented via two main strategies: intracortical implantation using penetrating microelectrode arrays (MEAs) and cortical surface recording using electrocorticography (ECoG). ECoG can be further divided into epidural and subdural configurations, depending on whether electrodes are placed above or beneath the dura mater in contact with the cortical surface [29, 30, 31]. Although MEAs provide high‐quality neural signals, they require craniotomy and penetration of brain tissue, which introduces surgical risks and limits long‐term scalability. Signal degradation over time remains a major challenge, often necessitating repeated recalibration. In addition, implantation‐related trauma can trigger reactive gliosis and scar formation, creating insulating barriers that attenuate signal transmission [29, 32, 33]. By contrast, ECoG represents a semi‐invasive alternative that can be deployed through burr holes without penetrating the cortex, thereby reducing tissue damage and improving long‐term stability [30]. Recent advances in ECoG systems, including wireless signal transmission, have further reduced infection risk and improved clinical feasibility [34]. However, ECoG is inherently limited to recording activity from superficial cortical regions.

**TABLE 1:** Comparison of BCI signal acquisition technologies.

Type	Technology	Invasiveness	Signal type	Temporal resolution	Spatial resolution	Advantages	Limitations	Reference
Invasive	MEAs	Penetrating the cerebral cortex	LFP and spikes	Ultra‐high (< 1 ms)	High (10–100 µm)	Enables single‐neuron recording and fine motor control; supports continuous use for months to years; LFPs provide a stable basis for long‐term decoding.	Requires craniotomy with infection risk; signal degradation over time due to immune response and loss of spike activity.	[28, 32, 33]
	ECoG	Epidural or subdural	LFP	High (ms level)	High (mm level)	Higher spatial resolution and bandwidth than EEG; less susceptible to EMG/EOG interference; lower tissue damage than MEAs.	Requires burr hole surgery; limited long‐term stability data.	[30, 31]
	fUS	Epidural	Blood flow	High (ms level)	High (µm level)	Sensitive to hemodynamic changes; useful in motor decoding research.	Still in early development; limitations not fully characterized.	[21, 27]
Interventional	Endovascular electrode arrays	Endovascular placement	LFP	High (ms level)	High (mm level)	Minimally invasive; avoids craniotomy; signal quality intermediate between EEG and ECoG.	Requires long‐term anticoagulation; implantation of subclavicular transmitter increases cost; vascular stent placement is irreversible.	[29, 35]
Non‐invasive	EEG	None	Scalp electrical activity	High (ms level)	Low (cm level)	Excellent temporal resolution; safe, accessible, and cost‐effective.	Lower signal quality; susceptible to EMG/EOG artifacts; requires conductive media.	[20, 22]
	MEG	None	Magnetic fields	High (ms level)	Medium‐high (mm‐cm level)	Direct measurement without skull distortion; complementary to EEG.	Expensive; requires shielded environment; limited sensitivity to deep sources.	[23, 24]
	fMRI	None	BOLD signal	Low (s level)	High (mm level)	High spatial resolution; whole‐brain coverage; integrates structural and functional imaging.	Low temporal resolution; expensive; not suitable for movement tasks.	[25, 26]
	fNIRS	None	Hemodynamic changes	Medium‐low (s level)	Medium (cm level)	Portable; tolerant to motion; suitable for bedside and pediatric use.	Limited penetration depth; signals may be contaminated by superficial tissues.	[24, 25]
	fTCD	None	Blood flow velocity	Medium (s level)	Low (cm level)	Safe and low cost.	Requires coupling agents; limited spatial resolution.	[23]

Abbreviations: BOLD, blood oxygenation level dependent; ECoG, electrocorticography; EEG, electroencephalography; EMG, electromyography; EOG, electrooculography; fMRI, functional magnetic resonance imaging; fNIRS, functional near‐infrared spectroscopy imaging; fTCD, functional transcranial doppler imaging; fUS, functional ultrasound imaging; LFP, local field potential; MEAs, microelectrode arrays; MEG, magnetoencephalography.

More recently, endovascular electrode arrays have emerged as a promising alternative for neural signal acquisition in BCI systems [35]. Unlike MEAs, which require craniotomy, or ECoG, which involves burr hole placement, endovascular devices are delivered through the vascular system into intracranial blood vessels. By utilizing the brain's natural vascular network, this approach enables access to target regions in a minimally invasive manner. Such technology holds particular promise for neurological conditions characterized by motor impairment, including stroke [35]. From a safety perspective, patients with stroke, who are often medically fragile and may present with coagulation abnormalities, may not tolerate the invasiveness of craniotomy [36, 37]. In contrast, endovascular approaches are already well established in clinical practice. Digital subtraction angiography (DSA) via endovascular access remains the gold standard for cerebrovascular imaging, and endovascular thrombectomy is widely used in the management of ischemic stroke [38, 39, 40]. These routine applications provide strong evidence supporting the safety and reliability of endovascular techniques. Importantly, emerging studies indicate that endovascular electrode arrays can achieve neural recordings with spectral content and bandwidth comparable to those obtained from epidural surface electrodes, enabling high‐fidelity and stable long‐term signal acquisition [41]. Moreover, based on the brain's extensive vascular network, endovascular systems may offer broader spatial coverage than conventional invasive electrodes [42]. Early clinical investigations have demonstrated that endovascular BCI systems can enable patients with severe upper limb paralysis to control digital devices, providing initial evidence of safety and feasibility in human subjects [35]. This approach is currently being evaluated in ongoing clinical trials (ClinicalTrials.gov identifier: NCT05035823). Collectively, these findings highlight the considerable promise of endovascular BCI for the rehabilitation of motor deficits, particularly in stroke, and highlight the need for further investigation.

Neural stimulation technologies modulate central and peripheral nervous system activity through targeted electrical interventions, thereby promoting neuroplastic processes such as synaptogenesis and functional reorganization [43]. Non‐invasive approaches, including transcranial direct current stimulation (tDCS) and repetitive transcranial magnetic stimulation (rTMS), modulate cortical excitability and have been explored for enhancing motor recovery after stroke and alleviating symptoms in psychiatric disorders such as depression [44]. However, current evidence demonstrating consistent and clinically meaningful benefits remains limited [43, 45]. In contrast, invasive neuromodulation techniques are advancing rapidly. In 2021, the U.S. Food and Drug Administration (FDA) approved vagus nerve stimulation (VNS) for the treatment of moderate‐to‐severe upper limb motor impairment in patients with chronic ischemic stroke [46]. DBS has also received FDA approval for neurological conditions including PD and epilepsy [47, 48]. Beyond these established therapies, emerging approaches, such as epidural spinal cord stimulation (ESCS), as well as non‐invasive modalities such as ultrasound stimulation and temporal interference stimulation, have shown promising therapeutic effects across a range of neurological and psychiatric disorders [49, 50]. Increasingly, evidence suggests that integrating these neuromodulation strategies into BCI‐based closed‐loop systems may further enhance treatment precision and improve clinical outcomes [51, 52, 53].

This review aims to synthesize recent advances in neural recording and neuromodulation technologies within the BCI framework and to evaluate their potential applications across major neuropsychiatric disorders. We begin by focusing on post‐stroke motor rehabilitation as a central paradigm, providing a detailed analysis of the mechanisms, clinical evidence, and future directions of endovascular BCI, BCI‐integrated ESCS (BCI‐ESCS), and BCI‐driven DBS (BCI‐DBS). We then extend the discussion to movement disorders such as PD and epilepsy, as well as cognitive and psychiatric conditions including Alzheimer's disease (AD) and depression, highlighting how BCI‐enabled recording and stimulation approaches can support diagnosis, symptom monitoring, and therapeutic neuromodulation. We also briefly summarize emerging applications in other neurological and psychiatric conditions. Finally, we address the ethical and societal considerations associated with the clinical translation of BCI technologies.

## 2 Motor Disturbances

Motor disturbances constitute a major source of disability in neurological disorders and arise from disruptions within the complex neural circuits that govern voluntary movement. Although the underlying etiologies and pathophysiological mechanisms differ, conditions such as stroke, PD, and epilepsy share a common feature of motor circuit dysfunction, making them important targets for BCI‐based interventions. In this section, we adopt a structured approach. We begin with stroke as a central and instructive model (Section 2.1), as its focal vascular pathology provides a well‐defined framework for examining both mechanisms and clinical applications. In particular, we highlight three emerging technologies: endovascular BCI for neural signal acquisition, and BCI‐ESCS and BCI‐DBS for motor restoration. Building on this foundation, we then turn to PD (Section 2.2), a progressive neurodegenerative disorder in which closed‐loop DBS, guided by real‐time detection of pathological biomarkers such as β‐band oscillations, has been extensively developed and clinically validated. Finally, we discuss epilepsy (Section 2.3), illustrating how BCI‐inspired responsive neurostimulation systems use real‐time seizure detection to deliver on‐demand therapy, representing a distinct application of closed‐loop control for episodic motor and non‐motor events.

### 2.1 Stroke

Stroke is defined as an acute neurological deficit resulting from cerebrovascular events, either ischemic or hemorrhagic, that disrupt cerebral perfusion. It is characterized by a high incidence, substantial risk of recurrence, significant disability and mortality rates, and a considerable socioeconomic burden [54]. Currently, stroke is the second leading cause of death worldwide and the leading cause of long‐term adult disability. With advances in acute stroke care, particularly interventional therapies, an increasing number of patients now survive the initial event and transition into the chronic phase of recovery [55]. Despite improved survival, up to half of stroke survivors experience persistent disabilities, including impairments in motor, cognitive, language, and speech functions [56]. Among these, motor dysfunction, especially involving the upper extremities, is the most prevalent and has a profound impact on independence in activities of daily living [57, 58]. The underlying pathophysiology primarily involves structural damage or functional disconnection of descending motor pathways, most notably the corticospinal tract (CST), following ischemic or hemorrhagic injury [59]. Current rehabilitation strategies for post‐stroke motor recovery are largely based on conventional physical therapy, incorporating both active and passive training approaches. These typically rely on repetitive, task‐specific practice to drive neuroplasticity and include interventions such as constraint‐induced movement therapy, neuromuscular electrical stimulation, robotic‐assisted training, motor imagery, and virtual reality‐based therapies [60]. Although these approaches can promote functional recovery to some extent by enhancing neuroplasticity [61, 62], their effects often plateau within approximately 6 months after stroke onset [8], yielding only modest improvements relative to untreated patients. As a result, many individuals fail to regain the level of function required for independent daily living [63]. In addition, patients with severe motor impairment are often unable to participate effectively in conventional rehabilitation programs due to insufficient residual motor function, which is typically required to engage in and benefit from these therapies [16]. This limitation highlights the need for novel rehabilitation strategies that can be applied across a broader spectrum of stroke severity and that more effectively promote motor recovery. Given that functional restoration after stroke is fundamentally dependent on neuroplasticity, interventions capable of enhancing and sustaining plastic changes have become a key focus of research [25].

BCI‐based neurorehabilitation represents one such promising approach. By enabling patients to actively engage in modulating their own neural activity, BCI systems shift rehabilitation from a largely passive process to a more interactive and adaptive one, with the potential to improve functional outcomes [64]. Two main categories of BCI systems have been explored to improve quality of life in individuals with stroke (Figure 1). Assistive BCIs are designed to bypass damaged neural pathways by allowing direct communication with and control of external devices [65]. As noted above, endovascular BCI systems, comprising intravascular electrode arrays interfaced with external digital platforms, can support functions such as communication and internet use in patients with severe motor impairment. In contrast, rehabilitative BCIs aim to restore lost function by promoting both functional and structural neuroplasticity. This is achieved through several key mechanisms: (i) neurofeedback training, (ii) reinforcement‐based operant conditioning, (iii) repeated task engagement to strengthen neural circuits, and (iv) Hebbian learning principles [15, 65]. Importantly, assistive and rehabilitative BCIs are not mutually exclusive. Some systems initially provide external assistance for movement but, when combined with sustained training, can facilitate the recovery of voluntary motor control over time [16, 66]. Neural stimulation has also emerged as a key component of post‐stroke motor rehabilitation. For example, functional electrical stimulation (FES) has been widely used as an open‐loop intervention to improve motor function [25, 45, 67]. More recently, closed‐loop systems that integrate BCI with FES (BCI‐FES) have demonstrated superior outcomes compared with FES alone, likely due to their ability to link neural intent with real‐time motor output [65, 67, 68]. Building on this concept, emerging neuromodulation strategies combined with BCI may further enhance recovery. In particular, ESCS and DBS have shown considerable promise for both immediate functional improvement and longer term motor recovery in patients with stroke [51, 69, 70].

This section focuses on key clinical studies, current applications, and future directions of endovascular electrode arrays, ESCS, and DBS. Special emphasis is placed on the therapeutic potential of integrating these neural recording and stimulation technologies within BCI frameworks for post‐stroke motor rehabilitation.

#### 2.1.1 Endovascular BCI for Stroke

The concept of endovascular BCI can be traced back to 1973, when Penn and colleagues demonstrated the feasibility of endovascular electroencephalography by positioning electrodes within distal branches of the middle cerebral artery via the internal carotid artery [71]. This pioneering work introduced a novel approach for detecting abnormal neural activity in deep brain regions, signals that were not accessible using conventional scalp EEG, particularly in patients with refractory epilepsy. Subsequent advances led to the development of transvenous approaches, which enabled safer and more stable long‐term implantation with continuous signal monitoring [72]. Furthermore, the relatively low complication rates associated with intracranial venous sinus stenting, combined with ongoing challenges in achieving reliable neural signal acquisition, have accelerated the development of stent‐based endovascular electrode arrays for BCI applications [73].

**FIGURE 2:**
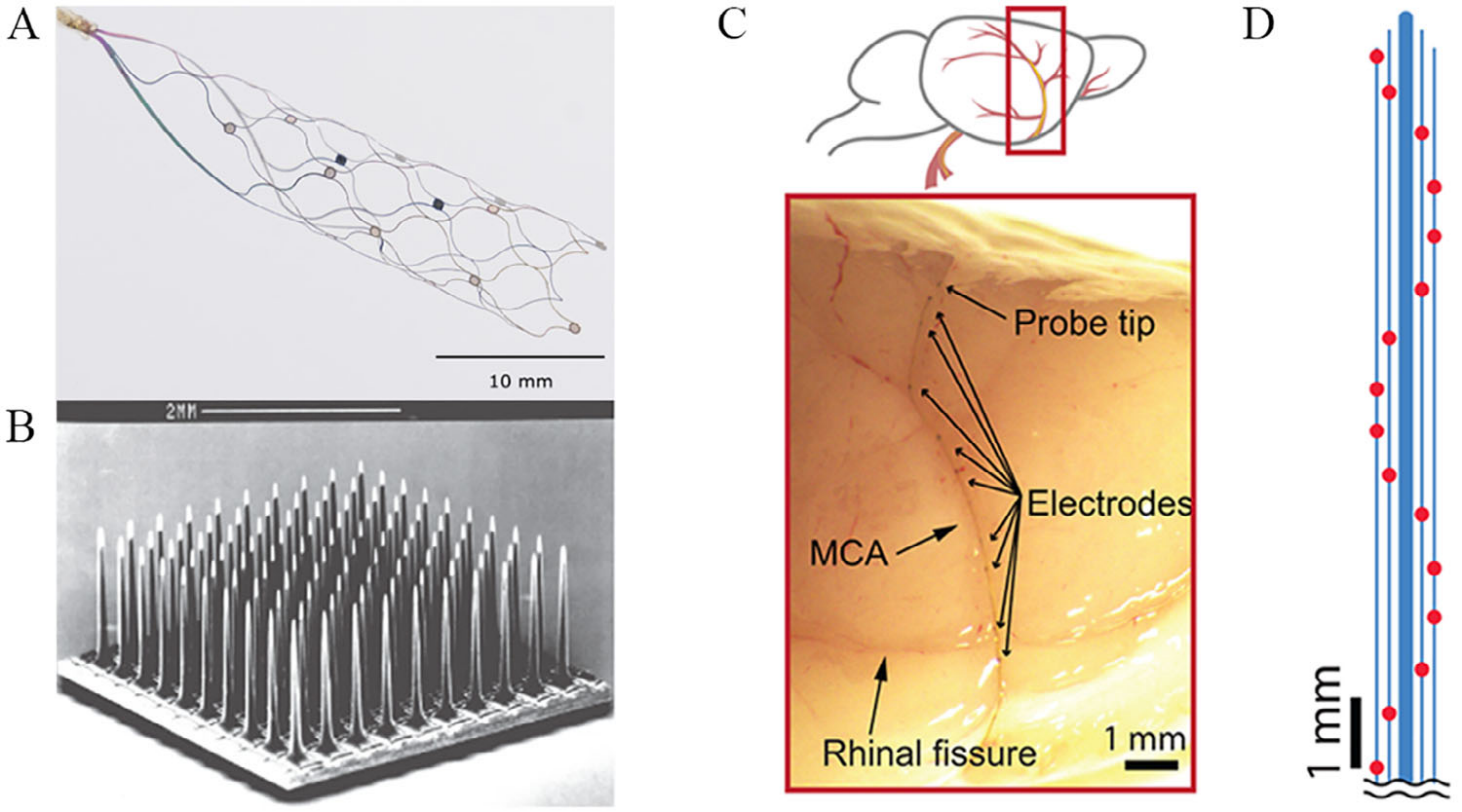
Structural diagrams of the Stentrode, Utah Arrays, and ultra‐flexible endovascular electrode. (A) The Stentrode: sixteen electrodes are arrayed around a nitinol scaffold measuring 8 mm × 40 mm. Authorization was obtained to make minor modifications [74]. Copyright 2021, The Author(s). Published by BMJ Publishing Group Ltd. (B) Utah Arrays, comprising 100 electrodes, features 96 signal‐conducting electrodes, in addition to the four corner electrodes. Authorization was obtained to make minor modifications [95]. 2017 Elsevier Ltd. All rights reserved. (C) Ultra‐flexible endovascular electrode was implanted into MCA of a rat, with a diameter less than 100 µm. (D) The electrode distribution map of the ultra‐flexible endovascular probe features a total of 16 electrodes arrayed across the probe's surface. MCA, the middle cerebral artery. (C and D) Authorization was obtained to make minor modifications [96]. Copyright 2023, The Authors, some rights reserved; exclusive licensee American Association for the Advancement of Science. No claim to original U.S. Government Works.

At present, the Stentrode (Synchron, Inc.) represents the most advanced endovascular stent‐based electrode array. This self‐expanding nitinol device (8 mm × 40 mm) incorporates 16 circumferentially arranged electrodes, each measuring 0.5 mm in diameter (Figure 2A). Under three‐dimensional digital subtraction angiography (3D‐DSA) guidance, the device is delivered via the jugular vein and deployed within the superior sagittal sinus, adjacent to the precentral gyrus of the primary motor cortex. The lead exits the venous system and is tunneled subcutaneously to an implanted telemetry unit positioned in an infraclavicular pocket. The recorded cortical electrical signals are transmitted to an external telemetry unit through infrared transmission, thus enabling external device control [35, 74]. Preclinical studies in sheep have evaluated the signal quality of endovascular electrode arrays in comparison with epidural and subdural ECoG. For instance, Oxley et al. implanted Stentrodes adjacent to the motor cortex in the superior frontal gyrus and performed endovascular recordings in awake animals for up to 190 days [41]. Signal bandwidth remained stable throughout the study period, and both the power spectral density and recording bandwidth were comparable to those obtained with epidural ECoG, although slightly lower than those achieved with subdural recordings. Notably, endothelialization of the stent began within 6 days of implantation, leading to integration with the vessel wall. This process is critical, as it stabilizes the device, reduces the risk of migration and thrombosis by minimizing exposed metallic surfaces, and may enhance signal acquisition by bringing the electrodes into closer proximity to the target cortical regions [75, 76]. In addition, John et al. demonstrated that, four weeks after implantation in ovine models, endovascular arrays achieved spatial resolution and signal‐to‐noise ratios comparable to both epidural and subdural ECoG systems [77].

Compared with epidural and subdural ECoG and MEAs, endovascular electrode arrays offer significant advantages in terms of safety and long‐term stability. As noted earlier, MEA implantation requires craniotomy, which carries risks such as intracranial hemorrhage, infection, meningitis, and disruption of the blood‐brain barrier [78]. Over time, implanted electrodes may also provoke tissue reactions, including gliosis and scar formation, leading to signal degradation and, in some cases, an increased risk of seizures [42]. These concerns are particularly relevant in patients with stroke, who are often medically fragile and may have coagulation abnormalities that further elevate surgical risk [37]. ECoG, which involves placing electrodes on the cortical surface through burr holes, is less invasive than MEA implantation but still imposes a nontrivial procedural burden, especially for patients with compromised health status. In contrast, endovascular approaches are markedly less invasive, avoiding both craniotomy and direct cortical exposure. As a result, they are associated with shorter recovery times and a reduced risk of procedure‐related complications. Importantly, techniques such as digital subtraction angiography and endovascular thrombectomy are already well established in stroke care, providing a strong clinical foundation for the safety and feasibility of endovascular interventions [38, 39, 79]. In addition, multiple studies have demonstrated the long‐term safety of transvenous electrode leads and intracranial venous sinus stenting [73, 80, 81, 82]. The first‐in‐human clinical trial of endovascular BCI, published in 2023, further supports these observations. Although conducted in patients with motor pathway impairment due to ALS and primary lateral sclerosis (PLS), conditions that share key features with stroke, such as corticospinal tract dysfunction and paralysis, the study provides important insights into device performance and durability [35]. The cohort included five participants (four with ALS and one with PLS), with four completing 12 months of follow‐up. Across this period, signal quality remained stable, with a mean bandwidth of 233 Hz and minimal variation between sessions, indicating strong long‐term recording stability. Imaging assessments using CT venography at 3 and 12 months post‐implantation revealed no clinically significant electrode migration and no evidence of vascular occlusion or thrombosis. These findings are consistent with preclinical data suggesting that endothelialization of the stent promotes stable integration with the vessel wall, thereby reducing the risks of device displacement and thrombotic complications [75, 76]. From a safety perspective, no device‐related serious adverse events resulting in death or permanent disability were reported. Functionally, the system demonstrated promising decoding performance, enabling the differentiation of five to seven attempted movement types. All participants successfully achieved computer‐based communication using the device, with a mean selection accuracy of 93.9%, an average typing rate of 16.6 correct characters per minute, and 97.2% accuracy in generated text. These results provide early evidence that endovascular BCI systems can serve as effective assistive technologies for individuals with severe motor impairment, and they highlight their potential relevance for stroke rehabilitation. Nevertheless, these findings should be interpreted with caution. The study was a small, early‐phase feasibility trial without a randomized controlled design, and thus does not provide high‐level clinical evidence. Moreover, the current follow‐up duration, ranging from 12 to 36 months in the ongoing trial (NCT03834857), is insufficient to fully assess long‐term durability over periods of 5–10 years or longer. Future large‐scale, well‐controlled clinical studies with extended follow‐up are therefore essential to determine how signal quality, device performance, and clinical benefit evolve over time, and to establish the suitability of endovascular BCI for patients with post‐stroke paralysis.

Most previous BCI studies in stroke have focused on decoding neural activity from the motor cortex, which imposes relatively strict inclusion criteria and typically limits participation to patients with preserved cortical structure and function [83, 84]. However, stroke pathology is highly heterogeneous. Ischemic stroke most commonly involves the middle cerebral artery (MCA) territory and frequently results in damage to the sensorimotor cortex [85, 86], whereas hemorrhagic stroke more often affects small vessels in deep brain regions, including the basal ganglia, thalamus, brainstem, and cerebellum [87]. As a result, current endovascular BCI approaches that target the motor cortex may be best suited to patients with intact cortical architecture, particularly those with ischemic stroke sparing critical regions, as well as many patients with hemorrhagic stroke. A key advantage of endovascular electrode arrays is their potential to access a broader range of neural signals compared with MEAs and ECoG. By utilizing the brain's extensive vascular network, these systems may expand the applicability of BCI to a wider population of stroke patients, including those with sensorimotor cortical damage. The cerebral vasculature provides natural pathways to both cortical and subcortical targets that are difficult to reach with conventional electrode technologies [42]. On the one hand, endovascular approaches may enable access to deep cortical regions, particularly within sulcal structures that are rich in motor‐related information. Notably, a large portion of the primary motor cortex (M1) lies within the central sulcus, and signals from this region are highly informative for decoding both executed and imagined movements [78, 88]. On the other hand, this approach also opens the possibility of recording from subcortical structures involved in motor control. For example, the basal ganglia integrate inputs from the primary motor cortex, supplementary motor area, and premotor cortex, and play a vital role in coordinating movement [89, 90]. Blood vessels in proximity to these structures, such as branches of the anterior cerebral artery, may provide feasible routes for positioning endovascular electrodes [91]. However, it is important to note that subcortical decoding remains largely theoretical at present, as no studies have yet demonstrated reliable extraction of motor information from these regions using BCI systems.

Achieving sustained motor recovery after stroke will require control signals that are more precise and information‐rich than those currently obtained with endovascular BCI systems. In particular, effective operation of external devices that provide meaningful motor feedback, such as multi‐degree‐of‐freedom robotic prostheses [92] or FES [93], depends on neural recordings with high spatial resolution and a favorable signal‐to‐noise ratio [94]. Two main strategies may improve the performance of endovascular electrode arrays: increasing electrode density and reducing device size. By comparison, commonly used intracortical microelectrode arrays, such as the Utah array, contain 100 electrodes (typically 96 active channels), and are often implanted in pairs to yield up to 192 recording channels [95]. In contrast, the current Stentrode platform includes only 16 electrodes [74]. Simply scaling up by adding more stent‐based devices is not practical; for example, achieving a comparable number of channels would theoretically require multiple implants within the superior sagittal sinus, which is neither feasible nor safe. Increasing electrode density within a single device is therefore a more realistic direction for development. Another promising approach is device miniaturization, which enables deployment in smaller vessels located closer to cortical targets. This strategy could improve both spatial resolution and signal quality [29]. Zhang et al. demonstrated an ultra‐flexible, 16‐channel endovascular electrode capable of being implanted into vessels as small as 100 µm in diameter in rodent brains [96] (Figure 2C,D). This design allowed neural recordings from regions supplied by both the middle cerebral artery and the anterior cerebral artery, effectively covering multiple cortical areas. Notably, the device was able to capture not only local field potentials but also single‐neuron activity, representing a substantial improvement in signal fidelity. Miniaturized endovascular arrays may also enable implantation across multiple sites within the dense vascular network of the sensorimotor cortex, facilitating simultaneous recordings from regions such as the supplementary motor area, premotor cortex, and posterior parietal cortex. Such distributed recording could enhance decoding of motor intent and ultimately support more precise motor control and functional recovery in patients with stroke [14, 29, 97, 98].

In summary, existing studies provide preliminary evidence supporting the feasibility of endovascular BCI for neural signal acquisition, with early demonstrations of improved communication, device control, and motor function in patients with paralysis following stroke. However, further high‐quality preclinical and clinical studies are needed to rigorously evaluate the safety, performance, and therapeutic efficacy of endovascular electrode arrays, particularly in stroke‐specific models and patient populations.

#### 2.1.2 ESCS for Stroke

Over more than five decades of development, ESCS has shown substantial promise in restoring motor function in individuals with spinal cord injury (SCI) and, more recently, in patients with post‐stroke paralysis. Notably, ESCS can enable the immediate recovery of certain motor abilities, allowing patients to perform complex activities such as walking, swimming, cycling, and object manipulation. In some cases, these improvements can be sustained beyond the stimulation period following structured rehabilitation [70, 99, 100]. Recent studies further suggest that combining ESCS with BCI systems in individuals with SCI‐related paraplegia leads to more natural motor control and greater long‐term functional recovery compared with ESCS alone [51]. This integrated approach offers a compelling new avenue for restoring upper limb function in patients with stroke. In the following sections, we examine the potential mechanisms underlying the combined use of BCI and ESCS for post‐stroke motor impairment, drawing on insights from SCI research and emerging evidence in stroke rehabilitation.

The origins of ESCS can be traced to the “Gate Control Theory of Pain” proposed by Melzack and Wall in 1965, which led to its initial application in the treatment of chronic pain, an indication that remains its only current approval by the US FDA [101, 102]. Interestingly, subsequent observations revealed that non‐patterned electrical stimulation of the lumbar spinal cord in individuals with complete SCI could evoke rhythmic, alternating leg movements. This finding provided direct evidence for the presence of central pattern generators (CPGs) within the human lumbosacral spinal cord [103]. CPGs were first identified in lower animals as neural networks within the spinal cord capable of generating rhythmic motor outputs independently of supraspinal input or sensory feedback [104]. This discovery opened new possibilities for applying ESCS to restore locomotor function in individuals with SCI. Experimental studies have shown that ESCS can activate these intrinsic spinal circuits to produce rhythmic motor activity even in the absence of descending input from the brain [105]. Moreover, animal studies in cats and rodents with spinal cord transection have demonstrated that task‐specific training combined with ESCS can enable the relearning of standing and walking functions [106, 107]. Building on these findings, early clinical evidence supporting the role of ESCS in motor recovery emerged in 2002 [108]. In that study, a patient with chronic incomplete SCI was able to achieve a near‐effortless and coordinated gait pattern during ESCS following a rehabilitation program that combined partial weight‐bearing training with continuous stimulation. The patient subsequently regained the ability to perform a range of functional activities in both home and community settings. Further mechanistic insights have been provided by Minassian and colleagues, who demonstrated how ESCS may engage and modulate spinal locomotor networks, including CPG‐related circuitry [109, 110, 111]. Spinal cord stimulation (SCS) promotes the depolarization of large‐diameter primary afferent fibers in the dorsal roots, which in turn activate interneurons and motor neurons within the lumbar spinal cord. These neural elements contribute to the control of lower limb movement through both monosynaptic and polysynaptic pathways, enabling frequency‐dependent modulation of motor output. Importantly, early studies demonstrated that initiating ESCS in combination with proprioceptive input, such as passive treadmill stepping, can immediately elicit functional movements that resemble natural gait. This finding highlights the critical role of sensory feedback in shaping motor output during stimulation and suggests that coupling ESCS with afferent input may enhance its therapeutic effects [112]. Building on this concept, Edgerton and colleagues proposed that, in the absence of supraspinal input, movement can be driven by sensory signals alone. ESCS increases the excitability of spinal circuits, allowing them to effectively process proprioceptive input by engaging CPGs. Through this mechanism, sensory feedback can modulate key aspects of movement, including speed, direction, and load‐bearing capacity [113, 114].

A major milestone in this field was the landmark case report published by Harkema et al. in 2011, which provided compelling clinical evidence for the efficacy of ESCS in individuals with severe paralysis following SCI [115]. In that study, a patient with chronic motor‐complete but sensory‐incomplete SCI underwent intensive weight‐bearing training combined with ESCS. Remarkably, within 7 months of implantation, the patient was able to achieve full weight‐bearing standing when stimulation was applied. Even more unexpectedly, partial recovery of voluntary motor function was observed. Electromyographic analyses suggested that ESCS does not directly generate movement; rather, it facilitates the activation of spinal circuits that are modulated by sensory input, even in the absence of clinically detectable supraspinal control. The recovery of voluntary movement was hypothesized to result from the re‐engagement of residual, previously subclinical descending pathways, as well as the formation of new connections through activity‐dependent plasticity. ESCS appears to raise the excitability of spinal interneuronal networks to a near‐threshold state, thereby amplifying the influence of weak supraspinal signals and enabling voluntary control via descending pathways. Subsequent clinical studies have extended these findings to patients with varying degrees of SCI severity [116, 117, 118, 119, 120] (Table 2). Despite differences in stimulation parameters and rehabilitation protocols, these studies consistently report improvements in standing and walking when ESCS is active, as well as partial gains in voluntary motor function even when stimulation is turned off. This growing body of evidence suggests that combining ESCS with task‐specific training and sensory feedback can provide both immediate functional assistance and promote longer term recovery of voluntary motor control.

**TABLE 2:** Summary of key clinical studies investigating ESCS in SCI and stroke.

							Stimulation parameters							
Author (Year)	Subjects (sex)	Years since injury	Injury level/AIS	Intervention	Stimulation site	Channels	Frequency (Hz)	Pulse width (µs)	Amplitude	Stimulation pattern	Optimization strategy	Evaluation criteria	Main findings	References
**Open‐loop ESCS for lower limb motor function in SCI**														
Herman et al. [108]	1 (M)	3.5	C5‐6 AIS C	ESCS + partial‐weight bearing therapy	Upper lumbar enlargement	8	Set between sensory and motor thresholds.			—	Multiple parameter sets tested to optimize gait.	Walking speed, step symmetry, perceived effort, physical capacity, metabolic activity.	Immediate improvements in gait rhythm; ESCS showed superior performance during long‐distance walking despite convergence in short‐distance outcomes.	[108]
Minassian et al. [112]	15	—	AIS A	ESCS + manually assisted treadmill stepping	Upper lumbar segments	4	2.2–50	210	1.0–10 V	Bipolar; frequency‐modulated sequences	—	Clinical examination and brain motor control protocol	5–15 Hz induced extension; 25–50 Hz produced rhythmic stepping; ESCS enhanced spinal network excitability and step‐like output.	[112]
Harkema et al. [115]	1 (M)	3.4	C7‐T1 AIS B	ESCS + intensive standing/walking training	L1‐S1	16	5–40	210 or 450	0.5–10 V	Bipolar; tonic (standing), rhythmic (walking)	Systematic parameter mapping for optimal motor patterns	EMG, kinematics, ground reaction forces	Regained partial supraspinal control after 7 months, but only with ESCS.	[115]
Angeli et al. [116]	4 (M)	2.2–4.2	C7 AIS B, T5‐T6 AIS A, C6‐C7 AIS B, T5 AIS A	ESCS + locomotor + home‐based training	L1‐S1	16	25–30	—	—	Bipolar; continuous	Individually optimized for joint‐specific movement	Force, endurance, EMG	Subthreshold ESCS enabled voluntary motor control in individuals with complete paralysis.	[116]
Rejc et al. [117]	4 (M)	2.2–4.2	C7 AIS B, T5‐T6 AIS A, C6‐C7 AIS B, T5 AIS A	ESCS + standing training	L1‐S1	16	25–60	—	1.0–9 V	Bipolar	Parameter adjustment during upright posture	EMG, ground reaction forces	Two AIS A participants achieved independent standing; sensory input critical for weight bearing.	[117]
Rejc et al. [120]	1 (M)	4.2	C7 AIS B	ESCS + activity‐based training	L1‐S1	16	30–65	—	0.4–3.5 V	Multipolar	Activity‐specific configurations (standing, stepping, voluntary movement)	EMG, kinematics, ground reaction forces	Long‐term ESCS led to recovery of voluntary control and independent standing without stimulation.	[120]
Gill et al. [118]	1 (M)	3	T6 AIS A	ESCS + multimodal rehabilitation training	L1‐S1	16	20–25	210	3.3–6 V	Multipolar; alternating programs	Interleaved programs for bilateral control	EMG, motor evoked potentials, MAS	Achieved independent standing and assisted overground walking with ESCS.	[118]
Angeli et al. [119]	4 (3 M, 1F)	2.5–3.3	T4 AIS A, T4 AIS A, C5 AIS B, T1 AIS B	ESCS + treadmill training	L1‐S1/S2	16	5–50	450	1.0–10 V	Bipolar/multipolar; tonic/non‐patterned/optimized stimulation	Periodic optimization every 2–4 weeks.	EMG, kinematics, kinetics	Enabled voluntary walking (AIS B) and treadmill walking (AIS A) with stimulation.	[119]
**Closed‐loop spatiotemporal ESCS for lower limb motor function in SCI**														
Wagner et al. [127]	3 (M)	4–6	C7 AIS C, C4 AIS D, C7 AIS C	Foot trajectory‐triggered ESCS + locomotor training	L1‐S1	16	20–120	300	0.6–8 mA	Monopolar/multipolar; spatiotemporal stimulation	EMG‐guided optimization	Kinematics, ground reaction forces, EMG	Rapid recovery of adaptive walking; voluntary control restored even without stimulation.	[126]
Rowald et al. [100]	3 (M)	1.3–8.9	T4 AIS A, T6‐T7 AIS A, T5‐T6 AIS B	Kinematics‐triggered ESCS + rehabilitation	T12‐S2	16	20 or 100	500	0.5 V	Monopolar/multipolar; spatiotemporal stimulation	Computational modeling + personalized programming	EMG, posture, kinematics	Restoration of multiple motor functions within one day; improved community mobility.	[100]
Lorach et al. [51]	1 (M)	10	C5‐C6	Brain‐triggered ESCS + rehabilitation	L1‐S1	16	40	300	14–16 mA	Monopolar/multipolar; spatiotemporal stimulation	Brain–machine interface‐driven parameter switching	Gait tests, neurological classification	Enabled natural movements and walking even without stimulation after training.	[51]
**Open‐loop ESCS for upper limb motor function in SCI**														
Lu et al. [142]	2 (M)	2–2.5	C5 AIS B, C6 AIS B	ESCS	C4/C5‐T1	16	2–40	210	0.1–10 mA	Bipolar; continuous	Electrode pair screening.	MEG, grip strength, ARAT	Approximately threefold increase in hand strength and improved voluntary control with ESCS.	[142]
**Open‐loop ESCS for upper limb motor function in stroke**														
Powell et al. [70]	2 (F)	3–9	Hemorrhagic/ischemic stroke	ESCS	C3‐T1	16	50–100	200–400	2.1–6.2 mA	Multipolar; continuous	EMG and preference guided	Strength, kinematics, safety	Immediate and sustained improvements in upper limb function even without training.	[70]

Abbreviations: AIS, American Spinal Cord Injury Association; ARAT, Action Research Arm Test; EMG, electromyography; ESCS, epidural spinal cord stimulation; MAS, Modified Ashworth Scale; SCI, spinal cord injury.

Despite these promising advances, current therapeutic strategies remain insufficient to fully reintegrate individuals with SCI into community life or restore independent activities of daily living. Preclinical and clinical studies investigating ESCS have proposed a unifying mechanistic framework: ESCS increases the excitability of residual spinal circuits below the level of injury by engaging primary sensory afferents within the dorsal roots, thereby enhancing the responsiveness of surviving CST inputs and facilitating the re‐emergence of supraspinal voluntary control over limb movements [70, 114, 116, 121, 122]. In addition, precise spatiotemporal alignment between descending supraspinal signals and ESCS‐induced activation of proprioceptive circuits is thought to strengthen residual synaptic connections through bidirectional spike timing‐dependent plasticity [123]. This mechanism may explain why the therapeutic effects of ESCS are often limited when conventional continuous, open‐loop stimulation paradigms are used. Although such stimulation can recruit afferent fibers, activate motor neurons, and elevate overall lumbosacral excitability, it may also interfere with the physiological transmission of proprioceptive signals along the same pathways [124]. These limitations have driven the development of closed‐loop, spatiotemporal ESCS approaches that are synchronized either with gait cycle phases [125, 126, 127] or with cerebral motor intentions [128, 129]. In particular, gait phase‐dependent stimulation aims to replicate the natural spatiotemporal patterns of motor neuron activation observed during normal locomotion [130, 131, 132]. Building on this concept, Wenger et al. [127] implemented foot trajectory‐triggered spatiotemporal ESCS [133] in individuals with both complete and incomplete SCI, achieving recovery of lower limb motor function within 1 week. Participants regained voluntary leg movements and were able to walk independently with crutches, with some functional gains persisting even after stimulation was discontinued. Subsequent refinements in both hardware and software enabled the same group to induce multi‐functional motor recovery, including standing and walking, in individuals with complete SCI within a single day [100]. Following 5 months of rehabilitation, participants achieved meaningful improvements in community mobility. These findings highlight the superiority of closed‐loop, spatiotemporal stimulation over conventional continuous paradigms, as it not only enables rapid restoration of volitional motor control in individuals with severe paralysis but also promotes durable, plasticity‐mediated functional recovery.

Another emerging paradigm in closed‐loop spatiotemporal ESCS is the use of cerebral motor intention to establish a brain‐spinal interface, commonly referred to as a BCI‐ESCS system [134]. Theoretically, BCI‐driven ESCS addresses several limitations inherent to gait phase‐based closed‐loop systems. Compared with direct brain‐intention control, gait cycle‐dependent stimulation is subject to temporal delays and reduced precision, limiting its ability to synchronize perfectly with voluntary movement. Moreover, in individuals with complete paralysis following stroke or SCI, anticipated movements cannot be reliably inferred from lower limb kinematics, rendering gait phase‐based strategies ineffective [100, 133]. This approach also lacks the capacity to dynamically adjust stimulation amplitude, thereby constraining voluntary control and limiting adaptability to complex, real‐world environments [51]. In 2016, Capogrosso et al. pioneered a BCI‐ESCS framework by integrating motor cortical activity with spatiotemporal stimulation. In non‐human primates, this system restored weight‐bearing capacity in paralyzed limbs within 6 days after SCI, without the need for prior training [128]. Subsequent work introduced a proportional BCI‐ESCS paradigm capable of continuously modulating stimulation amplitude in real time. Using this approach, paralyzed rodents rapidly regained overground locomotion and were able to adapt foot elevation for tasks such as stair climbing, with minimal calibration and no pretraining [129]. Notably, brain‐controlled stimulation both accelerated and enhanced long‐term motor recovery compared with continuous spinal stimulation alone. Building on these preclinical advances, Lorach et al. translated this technology to humans by implanting cortical and spinal interfaces in an individual with incomplete cervical SCI [51]. The system decoded motor intentions, including movement probability, amplitude, and direction, from electrocorticographic signals and converted them into stimulation commands delivered via an implanted pulse generator. Compared with gait phase‐based spatiotemporal ESCS, the BCI‐driven system enabled more natural and precise motor control, helped overcome functional recovery plateaus, and improved voluntary movement even in the absence of stimulation [127]. Ultimately, the participant achieved independent, crutch‐assisted walking [51, 135]. These findings indicate that BCI‐ESCS may induce greater neuroplastic changes in residual neural pathways than either continuous open‐loop stimulation or gait phase‐based closed‐loop approaches. By effectively re‐establishing communication between the brain and spinal cord, this strategy simultaneously engages neural circuits above and below the lesion, promoting Hebbian‐like reorganization of spared connections [123, 136, 137]. Such mechanisms hold significant promise for enhancing motor recovery in individuals with neurological impairments following stroke and SCI.

Recent advances in ESCS and BCI‐ESCS for lower limb rehabilitation have stimulated growing interest in cervical stimulation as a strategy to restore upper limb motor function [138]. Emerging evidence suggests that this approach holds considerable promise for improving arm and hand function after stroke [139]. Notably, upper and lower limb motor systems differ substantially in both organization and functional demands [118, 119, 127]. Locomotion relies primarily on relatively stereotyped, cyclic movement patterns, whereas upper limb function, particularly hand use, requires fine motor control, precise sensory feedback, and strong dependence on CST‐mediated voluntary input [138, 140, 141]. The first clinical study targeting upper limb paralysis with ESCS, reported in 2016, involved implantation of cervical epidural electrode arrays in two individuals with motor‐complete SCI [142]. After 20–22 stimulation sessions, grip strength during stimulation increased approximately threefold from baseline, accompanied by improvements in voluntary hand control. However, compared with the robust gains observed in lower limb function, continuous ESCS has generally produced more limited improvements in the upper extremities [118, 119]. This discrepancy likely reflects the non‐specific nature of continuous stimulation, which can disrupt the physiological transmission of proprioceptive signals and reduce its effectiveness in restoring fine motor control [124]. To overcome these limitations, more targeted neuromodulation strategies have been developed. For instance, personalized stimulation protocols that selectively engage individual cervical dorsal root afferents have been combined with BCI‐driven control. In preclinical studies, this approach enabled voluntary, autonomous motor control in macaques with unilateral cervical SCI, resulting in significant improvements in arm and hand function [143].

**FIGURE 3:**
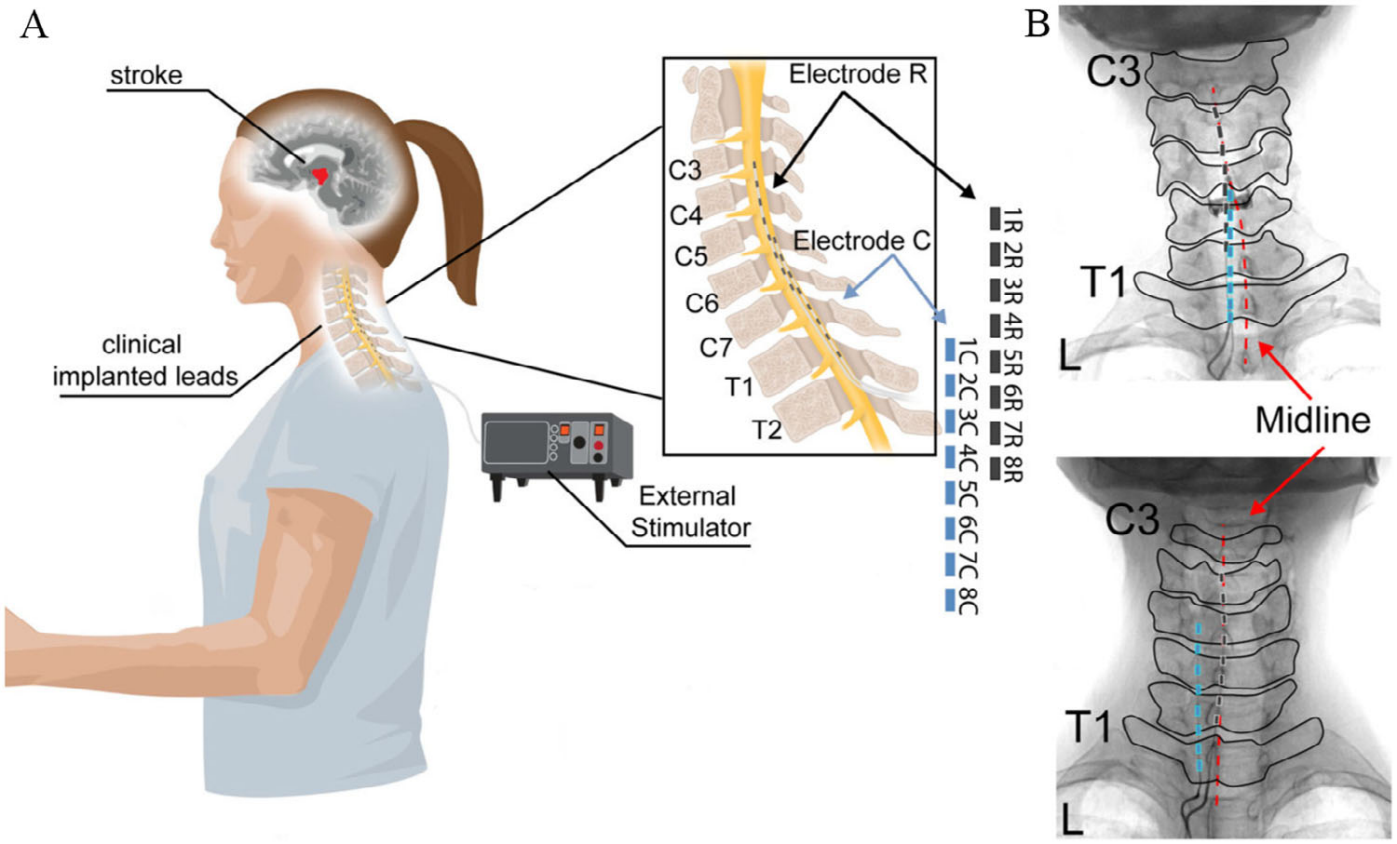
Schematic diagram and X‐ray images depicting the implantation of two staggered linear leads into the epidural region of the spinal cord on the hemiplegic side of two patients. (A) Schematic sagittal view illustrating two staggered linear leads spanning C3‐T1, with electrode R covering C3 to C6 and electrode C covering C5 to T1. (B) X‐ray images of two participants showing the positions of the rostral (blue) and caudal (dark gray) leads relative to the midline (red). (A and B) Authorization was obtained to make minor modifications [70]. Copyright 2023, The Author(s), under exclusive license to Springer Nature America, Inc.

Although most ESCS and BCI‐ESCS studies have focused on SCI, these approaches are highly relevant to stroke rehabilitation [144]. Upper limb motor deficits following stroke and SCI share common pathophysiological mechanisms, particularly disruption of CST pathways [59, 145, 146]. Importantly, however, stroke often spares a substantial proportion of descending projections, and spinal circuits below the lesion typically remain anatomically intact but functionally suppressed [147]. Because spinal stimulation can enhance the excitability of these preserved circuits [116], ESCS may yield even greater therapeutic benefit for upper limb recovery after stroke than after SCI [99, 138, 148]. To date, most studies of spinal stimulation in stroke populations have employed non‐invasive transcutaneous spinal cord stimulation (TSCS) [99]. Although TSCS has demonstrated modest improvements in motor function, these gains are generally insufficient to produce meaningful improvements in quality of life. Its limited efficacy likely reflects poor spatial specificity and interference with normal proprioceptive signaling [143, 149]. Powell et al. conducted the first clinical study investigating cervical ESCS for upper limb paralysis following stroke [70]. Owing to the greater anatomical length and segmental complexity of the cervical spinal cord compared with the lumbosacral region, the authors implemented a novel electrode configuration consisting of two staggered linear leads placed ipsilateral to the paretic limb within the epidural space. This design enabled broad and selective coverage of dorsal root entry zones from C3 to T1, overcoming the limited segmental reach of conventional paddle leads (Figure 3). After 29 days of continuous open‐loop ESCS, two individuals with moderate‐to‐severe post‐stroke hemiparesis exhibited immediate improvements in arm and hand strength, motor control, and functional performance during stimulation. Notably, these gains persisted to some extent even after stimulation was discontinued, despite the absence of structured motor training. Importantly, no serious adverse events related to electrode implantation or stimulation, such as pain exacerbation or limb rigidity, were reported, supporting the safety and feasibility of cervical ESCS for upper limb rehabilitation after stroke. Although this study primarily evaluated short‐term assistive effects under continuous stimulation, it provides an important proof of concept for clinical translation. Building on these findings, the integration of BCI‐ESCS with task‐specific motor training may offer a more effective strategy for achieving sustained functional recovery and facilitating meaningful reintegration into daily life. To date, BCI‐FES has emerged as a prominent closed‐loop approach in stroke rehabilitation [65, 68]. However, FES directly activates muscles to generate movement, often producing non‐physiological contractions that lead to rapid muscle fatigue and limit sustained force production [150]. In addition, BCI‐FES typically requires complex stimulation protocols and decoding algorithms, making the coordinated activation of multiple muscles and smooth execution of functional movements challenging [143, 151]. In contrast, ESCS modulates spinal circuits by recruiting dorsal root afferent fibers, which in turn engage motor neurons through trans‐synaptic mechanisms and amplify residual supraspinal control. This indirect mode of activation avoids the muscle fatigue associated with FES while preserving voluntary control of movement [116, 124, 152]. When combined with BCI technologies, ESCS can deliver real‐time, intention‐driven stimulation, providing immediate assistive effects and enabling more natural and precise motor control than continuous open‐loop paradigms [51, 128, 129, 153]. Moreover, BCI‐ESCS appears to confer substantial rehabilitative benefits beyond its assistive function, promoting durable neurological recovery through activity‐dependent neuroplasticity [51, 129]. This effect likely arises from the integration of closed‐loop neuromodulation with repetitive motor training, which reinforces residual neural pathways [154, 155]. Therefore, future clinical studies should prioritize the combination of BCI‐ESCS with structured rehabilitation protocols to maximize motor recovery in individuals with post‐stroke hemiparesis and ultimately improve long‐term functional outcomes and quality of life.

Although BCI‐ESCS demonstrates considerable promise for treating post‐stroke paralysis, several important challenges remain. Non‐invasive EEG, while effective for decoding motor intentions under controlled or static conditions, is highly susceptible to motion artifacts during everyday activities and is limited by relatively poor spatial resolution [25, 156]. In contrast, implanted cortical electrode arrays provide higher signal fidelity but are inherently invasive, carrying risks such as surgical complications and long‐term signal instability, concerns that are particularly relevant in patients with stroke, who may present with frailty or coagulation abnormalities [42, 78]. Thus, emerging endovascular BCI technologies may offer a compelling compromise by improving signal quality while minimizing surgical risk. Another major limitation lies in the spatial specificity of epidural spinal stimulation. Although current electrode designs can differentiate between major dorsal roots to facilitate gross flexion and extension of the upper and lower limbs, their resolution remains insufficient for fine motor control [70, 100]. This limitation is especially pronounced in the forearm and hand, where overlapping motor neuron pools make it difficult to selectively activate individual muscles. As a result, stimulation of single dorsal roots cannot reliably produce individuated finger movements, constraining the recovery of dexterous hand function in patients with stroke [70, 157, 158]. Future advances may improve targeting precision through high‐density electrode arrays and current steering techniques designed to selectively recruit specific afferent fiber populations [159]. Alternatively, combining ESCS with complementary approaches, such as FES, may help restore fine motor control by integrating the strengths of both modalities [68, 143]. Optimization of stimulation parameters also remains an unresolved challenge. Key variables, including frequency, amplitude, and pulse width, have complex and context‐dependent effects on motor output. For example, Minassian et al. have demonstrated that lower stimulation frequencies (5–15 Hz) preferentially induce extensor activation in the lower limbs, whereas higher frequencies (25–50 Hz) elicit rhythmic alternating flexion‐extension patterns [112]. However, as summarized in Table 1, there is substantial heterogeneity across clinical studies, with stimulation frequencies ranging from 2.2 to 120 Hz, pulse widths from 200 to 500 µs, and amplitudes spanning 0.4–10 V or 0.1–16 mA. Despite this variability, many parameter combinations have shown efficacy, often when paired with rehabilitation training or other interventions. Thus, no universally optimal parameter set has yet been established, highlighting the need for individualized, adaptive stimulation strategies tailored to each patient's neurophysiological profile.

Across studies, a wide range of parameter optimization strategies has been employed. Most commonly, investigators iteratively test multiple stimulation configurations and select the optimal set for each patient based on changes in target muscle EMG activity and motor performance. However, because most reports focus primarily on post‐optimization outcomes and provide limited detail on the optimization process itself, direct comparison of different strategies remains challenging. Recent technological advances are beginning to address this limitation and are opening new avenues for personalized therapy. For example, Rowald et al. redesigned the conventional 5‐6‐5 paddle lead, originally developed for dorsal column stimulation in pain management, to more selectively target spinal dorsal roots [100]. This modification enables more precise recruitment of muscle groups. They also developed software tools that allow rapid configuration of activity‐specific stimulation programs, facilitating real‐time adaptation of parameters for different tasks and supporting individualized motor rehabilitation. Similarly, Lorach et al. employed high‐resolution structural imaging to construct patient‐specific spinal cord models, enabling optimization of electrode placement prior to surgery. Final electrode positioning was further refined using intraoperative electrophysiological recordings. Taken together, these approaches highlight the importance of personalized electrode design and parameter tuning in improving functional outcomes [51]. Despite these advances, several key challenges remain. A deeper understanding of how specific stimulation parameters influence motor recovery is still needed, along with robust methods for tailoring both electrode configurations and stimulation settings to individual patients. In addition, the invasiveness of ESCS, particularly the biomechanical risks associated with laminectomy and device implantation, remains a major barrier to widespread clinical adoption in stroke populations [144, 159]. Non‐invasive alternatives, such as TSCS, have shown encouraging results and may help address these concerns. For instance, Moritz et al. applied TSCS to the cervical spinal cord using the ARC^EX^ device in 60 individuals with upper limb paralysis following SCI during structured rehabilitation [160]. After 2 months of therapy, no serious device‐related adverse events were reported, and 72% of participants achieved clinically meaningful improvements in strength and functional outcomes. Secondary analyses also demonstrated significant gains in hand strength, upper limb motor and sensory function, and patient‐reported quality of life. These findings support the safety and therapeutic potential of TSCS for upper extremity rehabilitation. However, as discussed above, TSCS is limited by its relatively low spatial specificity and its potential to disrupt normal proprioceptive signaling, which may constrain its overall efficacy. Further clinical studies are therefore required to determine whether TSCS can achieve substantial and durable motor recovery in patients with stroke [143, 149]. Finally, methodological limitations across existing studies, including small sample sizes, lack of blinding, and challenges in disentangling the effects of motor training from those of electrical stimulation, continue to hinder progress in the field [144, 159]. Addressing these issues through technological innovation and more rigorous study design will be essential for accelerating the clinical translation of BCI‐ESCS and maximizing its potential to improve motor recovery and quality of life in individuals with stroke.

#### 2.1.3 BCI‐DBS for Stroke

DBS is an established neuromodulatory therapy that involves the stereotactic implantation of electrodes into specific deep brain structures. These electrodes are connected via extension and lead to an implantable pulse generator, enabling chronic, adjustable electrical stimulation of targeted regions [148]. Standard DBS systems typically incorporate four to eight cylindrical contacts, allowing flexible and programmable delivery of stimulation. Key parameters, including contact configuration, amplitude, frequency, and pulse width, can be readily tailored to individual patients, supporting personalized treatment strategies [161]. Importantly, the effects of DBS are not limited to local target nuclei. Through distributed neural networks, stimulation can influence distant brain regions, including the cerebral cortex, thereby modulating pathological circuit activity [162]. DBS has been widely applied in the treatment of neurological disorders, with well‐established efficacy in movement disorders such as PD, essential tremor, and dystonia, for which it has received regulatory approval [25, 163]. In addition, growing evidence supports its safety and therapeutic potential in selected psychiatric conditions, including obsessive‐compulsive disorder (OCD) and major depressive disorder (MDD), as well as in neurodegenerative diseases such as AD [164]. Given its broad neuromodulatory capacity, DBS has attracted increasing interest as a potential treatment for post‐stroke sequelae [165].

To date, most studies have focused on maladaptive conditions following stroke, including central neuropathic pain [166], epilepsy [162], and hyperkinetic movement disorders (e.g., dystonia, tremor, and chorea) [167], where symptom overlap with established DBS indications has facilitated clinical translation. Although largely limited to case reports and small case series, these studies provide preliminary evidence supporting both the safety and efficacy of DBS in post‐stroke populations, with therapeutic effects observed even long after the initial insult [25, 162, 167]. In contrast, paralytic motor deficits, particularly hemiplegia, the most common and functionally disabling consequence of stroke, have received comparatively little attention in DBS research. This gap likely reflects several factors. Patients with severe hemiplegia are often medically fragile, which may limit their suitability for invasive neurosurgical procedures [36, 37]. Moreover, the historical focus of DBS has been on movement and psychiatric disorders, resulting in relatively limited preclinical and clinical investigation into its potential for restoring motor function after stroke or SCI [165]. Nevertheless, a small number of studies targeting other post‐stroke conditions have reported incidental improvements in motor function following DBS, implicating structures such as the posterior limb of the internal capsule as potentially relevant targets [162]. Although these findings remain preliminary and should be interpreted with caution, they suggest that DBS may have broader therapeutic effects on motor circuits than previously recognized. Building on this foundation, more recent preclinical and early clinical studies have begun to directly evaluate DBS as a strategy for enhancing motor recovery after stroke [69, 168, 169, 170, 171]. The relevant clinical studies are summarized in Table 3.

**TABLE 3:** Clinical trials of DBS for motor function recovery in patients with paralysis.

							Stimulation parameters					
Author (year)	Subjects (sex)	Type of injury	Years since injury	Severity of injury	Intervention	Stimulation site	Frequency (Hz)	Pulse width (µs)	Amplitude (mA)	Evaluation criteria	Main findings	References
Baker et al. [69]	12 (8 M, 4F)	Unilateral middle cerebral artery infarction (excluding diencephalon and basal ganglia)	1.2–3.4	Moderate to severe upper limb paralysis	DBS + upper limb rehabilitation (4–8 months)	Contralesional dentate nucleus of the cerebellum	30 or 185	90 or 200	4.4–9	Safety: Incidence of serious adverse events Efficacy: Fugl‐Meyer assessment for upper extremity, Arm Motor Ability Test Neuroimaging: ^18^F‐FDG PET/CT.	1. DBS combined with rehabilitation is safe and feasible 2. Significant motor improvement observed even up to 3 years post‐stroke 3. Improvement correlates with cortical reorganization 4. Patients with preserved distal motor function benefit most.	[69]
Ho et al. [168]	1 (M)	Severe bilateral CST lesions due to traumatic subarachnoid hemorrhage	—	Bilateral upper limb paralysis requires maximal assistance for daily activities	DBS	Bilateral motor thalamus	55	—	—	Electrophysiology: Motor evoked potential amplitude Behavioral: Muscle strength, force control, object manipulation Onset: Immediate effect with stimulation ON, disappears when OFF Safety: No adverse effects reported.	Increased motor evoked potential amplitude; improved grip strength; reduced force control errors; enhanced elbow extension during weight‐bearing; able to reach, lift, and bring objects to the mouth smoothly.	[168]
Cho et al. [171]	2 (1 M, 1F)	Chronic incomplete SCI (P1: T1 AIS C; P2: C5 AIS D)	5–16	P1: severe paralysis, requires walker and full‐length leg orthosis P2: moderate impairment, able to walk short distances independently.	DBS + lower limb rehabilitation (3 months)	Bilateral lateral hypothalamic area	20 or 40	60	2–10	Safety: Adverse events, vital signs, body weight, hormone levels Efficacy: ISNCSCI lower extremity motor score, 6‐min walk test, 10‐m walk test, electromyography, kinematics.	Safety: No serious adverse events Efficacy: Immediate gait improvement with DBS; long‐term functional gains after rehabilitation even when DBS off P1: independent from orthosis P2: able to ascend/descend stairs independently.	[171]

Abbreviations: AIS, American Spinal Cord Injury Association; CST, corticospinal tract; DBS, deep brain stimulation; F, female; ISNCSCI, International Standards for Neurological Classification of Spinal Cord Injury; M, male; P, participant.

As discussed above, post‐stroke motor deficits are largely attributable to damage to the CST, which disrupts communication between the motor cortex and lower motor centers. Although this damage is often incomplete, the remaining descending excitatory pathways are typically insufficient to effectively activate spinal motor neurons and support voluntary movement [172]. In principle, neuromodulatory strategies that restore connectivity across disrupted pathways or enhance residual signaling could promote motor recovery after stroke. A range of neurostimulation approaches provides preliminary support for this concept. Non‐invasive techniques such as tDCS and rTMS can increase motor cortex excitability and thereby enhance CST output [173, 174, 175]. At the spinal level, dorsal root electrical stimulation has been shown to increase the responsiveness of spinal motor neurons to residual CST input, potentially enabling the recovery of voluntary movement [70]. More recently, DBS has been explored for post‐stroke motor recovery based on similar mechanistic principles. Analogous to cortical stimulation [176], DBS is thought to modulate motor cortex excitability indirectly by targeting motor‐related deep brain nuclei, thereby augmenting residual CST output [69, 168]. In 2023, Baker et al. reported the first phase I clinical trial evaluating DBS for stroke rehabilitation. This study investigated continuous stimulation of the cerebellar dentate nucleus (DN) combined with an intensive, task‐specific rehabilitation program [69]. The trial enrolled 12 patients with moderate‐to‐severe upper limb hemiparesis 12–36 months after ischemic stroke. Electrodes were implanted in the contralesional DN, delivering continuous stimulation (24 h per day) for 4–8 months. This intervention was paired with a 14‐month rehabilitation program emphasizing highly repetitive, challenging, and functionally relevant upper limb tasks under therapist supervision. No serious perioperative or stimulation‐related adverse events were reported. Importantly, 9 of the 12 participants demonstrated motor improvement, with a median increase of seven points on the upper limb Fugl‐Meyer Assessment. Notably, therapeutic effects were observed even in patients treated up to 3 years post‐stroke, suggesting that the efficacy of DN‐DBS may be relatively independent of chronicity. Mechanistically, DN stimulation is proposed to activate the dentatothalamocortical pathway, thereby enhancing excitability in ipsilesional motor cortical regions, promoting cortical reorganization, and facilitating functional recovery [177]. This hypothesis is supported by extensive preclinical evidence [178, 179, 180, 181]. Consistent with this framework, metabolic imaging in the phase I trial indicated that DN‐DBS combined with rehabilitation increased motor‐related cortical excitability and promoted neuroplastic reorganization. These findings provide initial evidence supporting the safety and feasibility of DN‐DBS as a therapeutic strategy for motor recovery after stroke. However, as an open‐label, non‐randomized phase I study, this trial lacked the methodological rigor needed to disentangle the specific effects of DBS from those of intensive rehabilitation. Well‐designed randomized controlled trials are therefore essential to more precisely define the therapeutic contribution of DN stimulation in chronic stroke recovery. Complementary evidence has been provided by Ho et al., who demonstrated that targeted electrical stimulation of the motor thalamus can acutely enhance motor cortex excitability and CST activity, leading to immediate improvements in upper limb motor function following CST injury in both macaques and humans [168]. Using high‐definition fiber tracking, the authors identified the ventral laterolateral (VLL) nucleus in the macaque motor thalamus, corresponding to the ventral intermediate (VIM) and ventral oral posterior (VOP) nuclei in humans, as having the strongest projections to M1. In macaques, targeted stimulation of the VLL increased the excitability of corticospinal neurons within M1 and produced immediate improvements in upper limb motor output, including enhanced motor evoked potential amplitudes, improved movement kinematics, and greater grip strength in both intact and CST‐lesioned animals. In healthy participants, VIM and VOP nuclei were likewise shown to preferentially project to M1, and their stimulation increased motor cortical and CST excitability in a frequency‐dependent manner, resulting in immediate gains in motor performance. Notably, in a patient with severe bilateral CST damage, DBS implantation in the motor thalamus produced rapid and substantial improvements in strength, voluntary control, and overall upper limb function at optimal stimulation frequencies of 50–80 Hz. These benefits were reversible, disappearing once stimulation was discontinued. Compared with conventional DBS protocols (typically >100 Hz), the lower stimulation frequencies were associated with minimal side effects. However, further studies are required to determine whether such stimulation can induce durable, plastic changes that translate into long‐term functional recovery. Importantly, the immediate facilitatory effects of motor thalamic stimulation observed by Ho et al., together with the sustained improvements achieved through DN stimulation combined with rehabilitation in the study by Baker et al., parallel the dual mechanisms previously described for ESCS, namely, short‐term assistance of motor output and longer term promotion of neuroplastic recovery. DBS and ESCS likely share convergent mechanisms, both acting to potentiate residual CST pathways and thereby facilitate voluntary motor control after stroke. Building on this framework, integrating BCI technology with DBS to create a closed‐loop BCI‐DBS system may further enhance activity‐dependent plasticity. Similar to the synergistic effects reported with combined BCI and ESCS, such an approach could optimize rehabilitation outcomes by coupling neural intention with targeted neuromodulation, ultimately leading to greater and more sustained motor recovery in patients with stroke.

**FIGURE 4:**
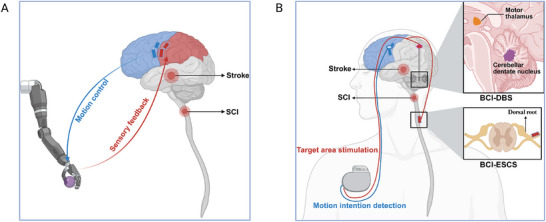
An overview of two types of bidirectional BCI. (A) Sensory feedback bidirectional BCI controls a robotic arm to grasp objects by detecting motor intent and provides sensory feedback through cortical stimulation of the somatosensory cortex using sensors from the robotic arm, thereby enabling enhanced control of the robotic arm. (B) Another form of bidirectional BCI facilitates motor function recovery by detecting motor intent from the motor cortex and stimulating other regions. When deep brain structures are stimulated, it forms a BCI‐DBS system, whereas stimulation of the spinal cord results in a BCI‐ESCS system. BCI, brain–computer interface; DBS, deep brain stimulation; ESCS, epidural spinal cord stimulation; SCI, spinal cord injury. This figure was created using BioRender.com.

BCI‐DBS can be conceptualized as a form of brain‐controlled brain stimulation (Figure 4B). Although this specific term has not been widely formalized, closely related concepts have been explored in the context of closed‐loop DBS, particularly for neuropsychiatric disorders associated with aberrant brain states, such as PD and depression [164, 182]. Closed‐loop DBS systems are designed to modulate pathological neural activity by delivering fixed or dynamically adjusted stimulation to targeted deep brain nuclei, guided by disease‐relevant electrophysiological biomarkers used as feedback signals [27, 161]. Depending on the control strategy, closed‐loop DBS can be broadly categorized into reactive and adaptive stimulation. Reactive systems deliver stimulation with predefined parameters in response to detected neural events, whereas adaptive systems continuously adjust stimulation parameters in real time according to fluctuations in neural biomarkers [183]. Most recent advances in closed‐loop DBS have focused on PD. For example, adaptive DBS (aDBS) systems that use elevated β‐band activity in basal ganglia local field potentials as a control signal have shown promising efficacy in alleviating motor symptoms [184]. Other studies have explored alternative control signals, including abnormal cortical γ activity [185] or decoded motor intentions [186] to drive stimulation in a more physiologically relevant manner. To date, however, BCI‐DBS has not been applied to neurological conditions characterized by paralytic motor deficits, such as stroke or SCI. Nevertheless, rapid progress in both BCI and DBS technologies highlights their therapeutic potential in neurorehabilitation [1, 25]. BCI‐based training has been shown to promote neuroplasticity, facilitating structural and functional reorganization in both cortical and subcortical regions, thereby supporting the recovery or relearning of motor functions after stroke [65]. Moreover, emerging DBS studies suggest that targeted stimulation can enhance cortical excitability and drive both immediate improvements in motor performance and longer term functional recovery [69, 168]. It is therefore plausible to hypothesize that combining these two technologies could synergistically promote neuroplasticity in the brain, potentially leading to more effective rehabilitation outcomes for stroke patients.

Two potential strategies can be envisioned for implementing BCI‐DBS in patients with stroke. The first, analogous to closed‐loop DBS in PD, uses electrophysiological biomarkers associated with post‐stroke motor deficits as feedback signals to drive stimulation in targeted brain regions. Previous studies have identified several characteristic abnormalities following stroke, including reduced cortical activity, slowing of electroencephalographic rhythms, diminished sensorimotor rhythm power, and increased interhemispheric asymmetry [187, 188, 189]. In principle, these features could serve as control signals for DBS. However, a key challenge is the identification of biomarkers that are both sufficiently specific and spatially localized to allow simultaneous sensing and modulation, similar to the use of pathological β oscillations in the subthalamic nucleus (STN) in PD [190]. A second, and potentially more promising, approach is to use motor intention as the control signal for DBS. This paradigm is already widely employed in BCI‐based stroke rehabilitation. Systems such as BCI‐FES [68] and BCI‐ESCS [51], which rely on decoding motor intention, have demonstrated encouraging improvements in motor recovery in paralyzed patients. Moreover, recent work showing successful motor intention‐driven thalamic DBS in patients with essential tremor provides preliminary evidence supporting the feasibility of this strategy [186]. At present, the most promising DBS targets for BCI integration appear to be DN [69] and the motor thalamus [168]. BCI‐DBS targeting the motor thalamus may offer immediate assistive benefits for patients with post‐stroke hemiparesis, as stimulation triggered by detected motor intent could transiently enhance motor output. In contrast, BCI‐DBS targeting the DN, when combined with structured physical rehabilitation, may be better suited to promoting longer term, sustained motor recovery, potentially exceeding the effects of conventional open‐loop DBS paired with rehabilitation.

Despite these promising early findings, the long‐term safety and tolerability of invasive stimulation of deep brain structures remain critical considerations for the broader application of BCI‐DBS in stroke populations. Adverse events associated with DBS generally fall into three categories: surgical complications, hardware‐related issues, and stimulation‐induced effects. Surgical risks include venous infarction and intracranial hemorrhage, both of which can result in permanent neurological deficits in a small proportion of patients [191, 192, 193, 194]. The overall risk of intracerebral hemorrhage is estimated at approximately 1%–2%, including minor cases [195, 196]. Seizures, a potential complication of any supratentorial procedure, occur in roughly 1% of DBS cases [197]. Hardware‐related complications include infection, skin erosion, lead migration, and lead fracture. Device‐related infections have been reported in approximately 3%–8% of patients, while lead migration or fracture occurs in about 2%–3% of cases [197]. Stimulation‐related adverse effects, arising from modulation of the target region or adjacent neural structures, may occur during or after treatment. Although these effects are often manageable through adjustment of stimulation parameters, they can limit the maximum tolerable stimulation intensity and, ultimately, the overall therapeutic benefit achievable with DBS [162, 165]. Despite these concerns, DBS is generally regarded as a safe and well‐tolerated intervention overall [197]. A systematic review examining DBS for post‐stroke movement disorders included 29 studies encompassing 53 patients, all of whom demonstrated clinical improvement. Notably, only two complications were reported: one case of speech disturbance and one hardware‐related infection [167]. Similarly, the clinical trial by Baker et al. evaluating DBS for post‐stroke motor rehabilitation placed strong emphasis on safety and tolerability [69]. In that study, 12 patients underwent 4–8 months of continuous stimulation combined with 14 months of rehabilitation, corresponding to a cumulative 168 months of electrode implantation and 72 months of active stimulation. No serious perioperative complications, such as infection, hemorrhage, or death, were observed, and no device malfunctions were reported. Although 51 adverse events occurred during the trial, only 21 were considered related to study participation, most of which were mild and transient, including pain, nausea, vomiting, and temporary weakness. These findings provide encouraging preliminary evidence supporting the safety of DBS in stroke populations. However, as the electrodes were explanted after 1 year, the long‐term safety profile and potential delayed adverse effects remain uncertain and warrant further investigation in larger and longer duration studies. Recent advances in non‐invasive DBS technologies offer promising alternatives that may mitigate the risks associated with surgical implantation. Approaches such as transcranial focused ultrasound (FUS) [198], transcranial focused magnetic stimulation [199], and transcranial temporal interference electrical stimulation (tTIS) [49, 200, 201] have demonstrated the ability to target deep brain structures and modulate neural activity non‐invasively. Among these, FUS has already entered early‐stage clinical research in stroke. In a phase I safety and feasibility study, Huang et al. administered low‐intensity FUS to 18 stroke patients and reported no serious adverse events [202]. Importantly, higher intensity stimulation was associated with improvements in motor learning and increased corticospinal excitability. tTIS has also been explored for thalamic stimulation in patients with PD. In a recent study, 20 min of targeted stimulation in eight patients was well tolerated and showed good safety and compliance, with preliminary indications of motor symptom improvement [203]. If these non‐invasive neuromodulation approaches can achieve therapeutic effects comparable to, or exceeding, those of invasive DBS, they may represent more accessible and scalable options for a broader population of stroke patients.

The major challenge in translating BCI‐DBS into clinical practice for post‐stroke motor rehabilitation is the lack of high‐quality evidence. Although studies by Ho et al. [168] and Baker et al. [69] have reported encouraging outcomes, these early investigations were primarily designed to assess safety and feasibility rather than efficacy. Well‐designed, blinded randomized controlled trials are therefore needed to define the optimal patient population, timing of intervention, and stimulation parameters, and to establish more robust evidence regarding the safety and therapeutic value of BCI‐DBS in stroke rehabilitation.

#### 2.1.4 Bidirectional BCI for Stroke

Based on the directionality of information flow between the brain and external devices, BCI systems can be broadly classified as unidirectional or bidirectional. Unidirectional BCIs transmit neural signals from the brain to external devices, typically with limited feedback (e.g., visual cues), and are most commonly used for communication and device control [204, 205]. In contrast, bidirectional BCIs operate as closed‐loop systems with “brain‐in‐the‐loop” functionality, enabling dynamic interaction between neural activity and external inputs [206]. Bidirectional BCIs can be implemented in two main ways. First, they can provide artificial sensory feedback, via stimulation of the brain or peripheral nerves, to mimic natural sensory input and improve control of external devices [207]. Second, they can transform recorded neural signals into electrical stimulation delivered to either the same or different neural regions, thereby facilitating activity‐dependent plasticity (e.g., Hebbian mechanisms) and promoting motor recovery [208] (Figure 4).

To date, one of the most notable examples of sensory feedback‐based bidirectional BCI is the neuroprosthetic system developed by Flesher et al. [66]. In this approach, microelectrode arrays were implanted in the motor cortex of paralyzed individuals to decode motor intention and enable control of a robotic arm for object manipulation. Simultaneously, arrays implanted in the somatosensory cortex delivered intracortical microstimulation to evoke tactile sensations based on sensory input from the robotic limb. This bidirectional configuration, incorporating real‐time somatosensory feedback, significantly improved patients’ ability to control the prosthetic device. By establishing a closed‐loop interaction between neural activity and external output, sensory feedback allows users to adapt more rapidly and effectively. Beyond improving task performance, this approach can reduce the cognitive burden of training and enhance user engagement, both of which are critical factors for achieving meaningful and sustained rehabilitation outcomes. A recent advance in the second category of bidirectional BCI is the brain–computer–brain system developed by Rosary et al., designed for personalized transcranial alternating current stimulation (tACS) [209]. In this approach, the peak frequency of motor imagery‐related activity detected by EEG in healthy participants was used to deliver individualized tACS in real time. Preliminary findings revealed frequency‐specific effects of tACS on brain function, suggesting that personalized stimulation protocols may facilitate post‐stroke motor recovery by modulating motor cortical oscillations. Importantly, this work also demonstrates the feasibility of this bidirectional framework by addressing a key technical challenge, namely, the potential interference between simultaneous neural recording and stimulation. Such systems could achieve truly “precision rehabilitation,” in which stimulation parameters are continuously adjusted based on real‐time neural activity in patients undergoing stroke recovery. Within the broader bidirectional BCI framework, one of the most clinically promising approaches at present is the BCI‐FES system. FES can evoke functional movements by recruiting proprioceptive pathways, including Golgi tendon organs and muscle spindle afferents, thereby providing rich somatosensory feedback to the central nervous system [210]. As a result, BCI‐FES not only fulfills the criteria of bidirectional BCI with sensory feedback but also aligns with the second paradigm, in which neural signals are transformed into stimulation delivered to different anatomical regions. Strong clinical evidence supporting this approach comes from a randomized controlled trial conducted by Biasiucci et al. [68]. In that study, 27 stroke patients were randomly assigned to either a BCI‐FES group (*n* = 14) or a sham‐FES control group (*n* = 13). Participants completed 10 sessions of motor intention‐based hand extension training over a 5‐week period. Notably, only the BCI‐FES group demonstrated significant improvements in upper limb motor function, and these gains were sustained for 6–12 months after the intervention. These findings highlight bidirectional BCI as a key future direction for enhancing motor recovery after stroke.

Beyond its capacity for neural signal recording, endovascular BCI has also been explored as a platform for electrical stimulation. Preclinical studies have demonstrated that endovascular stimulation of the motor cortex in sheep can successfully evoke facial and limb movements, providing early evidence for its potential use in cortical neuromodulation and motor recovery after stroke [211]. In addition, Neudorfer et al. investigated the feasibility of targeting deep brain structures via the vascular system by mapping neuroanatomical DBS targets onto probabilistic vascular atlases derived from healthy individuals [212]. Their analysis suggested that several key structures implicated in post‐stroke motor dysfunction are anatomically accessible through endovascular routes [169, 213]. These include the dentato‐rubro‐thalamic tract associated with the superior cerebellar artery (specifically the S2 segment, corresponding to the pathway from the DN to the motor cortex), as well as the STN, which is supplied by the anterior choroidal artery. Importantly, available evidence indicates that the adverse effects of endovascular stimulation are broadly comparable to those associated with conventional electrode implantation, while still preserving the ability to effectively entrain neural activity within target regions. A key theoretical advantage of this approach is the possibility of deploying multiple electrode arrays across different vascular territories, thereby enabling simultaneous modulation of several brain regions involved in motor recovery. On this basis, endovascular BCI offers dual therapeutic potential. First, by establishing a bidirectional interface within the primary sensorimotor cortex, it can provide sensory feedback and enhance control of external devices. Compared with traditional microelectrode arrays, this approach is considerably less invasive and causes less tissue damage, making it more suitable for a wider population of stroke patients. Second, through coordinated, multi‐site stimulation, endovascular systems could support a second form of bidirectional BCI, in which distributed neural circuits are simultaneously modulated, potentially yielding synergistic effects on neurorehabilitation outcomes. However, realizing these advantages will require further technological advances. Current systems rely largely on implanted pulse generators with wired power delivery, which limits the number of electrodes that can be deployed and increases the risk of vascular injury and thrombosis [214]. Future progress will depend on the development of endovascular devices capable of wireless data transmission and energy delivery.

BCI‐ESCS can be viewed as a representative form of bidirectional BCI. At the spinal level, ESCS enhances the excitability of spinal circuits through dorsal root stimulation, thereby facilitating the integration of proprioceptive inputs and responsiveness to voluntary motor commands transmitted via residual corticospinal pathways. In this way, descending cortical motor signals are effectively coupled with ascending sensory feedback, forming a closed‐loop system that exemplifies bidirectional BCI with sensory feedback [70, 114, 116, 121, 122, 123]. At the same time, BCI‐ESCS also aligns with the second form of bidirectional BCI. By decoding motor intention signals from cortical motor areas and using them to modulate stimulation of specific spinal dorsal roots, this approach promotes activity‐dependent neuroplasticity and facilitates the recovery of voluntary motor function [51]. Compared with FES, ESCS offers important physiological advantages. FES directly activates muscles to generate movement, which can lead to rapid muscle fatigue and limit sustained force production, making it less suitable for long‐term rehabilitation [150]. In contrast, ESCS recruits dorsal root afferent fibers and engages spinal motor neurons trans‐synaptically, allowing movements to remain under voluntary control. This mechanism not only reduces fatigue but also better preserves natural motor patterns [116, 124, 152]. Therefore, BCI‐ESCS, by incorporating bidirectional control and sensory feedback, represents a significant advancement in the rehabilitation of patients with paralysis and may ultimately achieve more durable and meaningful recovery than BCI‐FES in stroke populations.

BCI‐DBS may likewise support post‐stroke rehabilitation through bidirectional BCI principles, particularly by restoring or augmenting sensory feedback. A key advantage of DBS is its ability to access deep cortical and subcortical structures via intrinsic neural circuits, including regions that are difficult to reach with surface or intracortical electrodes [162]. For example, area 3a of the primary somatosensory cortex, located deep within the central sulcus and critical for processing proprioceptive input from the thalamus, is typically inaccessible to conventional microelectrode arrays [215]. Thalamic DBS offers a potential alternative for re‐establishing proprioceptive feedback, as stimulation of thalamic nuclei has been shown to evoke sensations corresponding to specific receptive fields [216]. Moreover, BCI‐DBS systems that decode cortical motor intentions to drive stimulation of deep brain targets, such as the DN or motor thalamus, represent clear examples of the second form of bidirectional BCI. Although such approaches have not yet been applied in stroke populations, their feasibility is supported by related work in other neurological conditions. For instance, Herron et al. demonstrated a closed‐loop DBS system in a patient with essential tremor, in which cortical activity recorded via electrocorticography was used to detect movement‐related beta desynchronization [186]. Stimulation was delivered to the ventral intermediate nucleus of the thalamus only when movement intention was detected, effectively suppressing tremor and enabling more normal motor performance. Although bidirectional BCI remains an emerging field, ongoing theoretical and experimental advances suggest that BCI‐DBS holds considerable promise. By integrating intention‐driven control with targeted DBS, this approach may ultimately enable more naturalistic motor function and more effective functional recovery in patients with stroke [1].

#### 2.1.5 Patient Selection for Targeted BCI‐Based Therapies

The successful clinical translation of BCI‐based neural recording and neuromodulation technologies depends critically on identifying patients who are most likely to benefit while minimizing procedural and long‐term risks. Selection among endovascular BCI, BCI‐ESCS, and BCI‐DBS should be guided by a comprehensive evaluation of stroke characteristics, including type, lesion location, and severity, as well as residual neurological function, comorbidities, and individual anatomical suitability. Given the limited number of clinical trials currently available, these recommendations remain largely inferential and are based on emerging evidence from early‐stage studies and related neurotechnological applications.

##### 2.1.5.1 Endovascular BCI

For endovascular BCI, the most suitable candidates are patients with moderate‐to‐severe upper limb paresis or paralysis in whom the primary motor cortex or adjacent sensorimotor regions remain structurally preserved and accessible for signal acquisition [29, 93]. This includes individuals with ischemic stroke affecting the middle cerebral artery territory but with relatively intact cortical areas within the relevant vascular distribution, such as regions adjacent to the superior sagittal sinus [29, 85, 86]. Patients with hemorrhagic stroke involving deep structures (e.g., basal ganglia, thalamus, or internal capsule) while sparing the overlying cortex may also be appropriate candidates, as cortical signals can still be recorded [29, 39, 87]. Importantly, this approach may be particularly advantageous for patients who are medically stable but at high surgical risk, such as those with frailty or coagulation disorders, because it relies on minimally invasive, endovascular techniques adapted from established neurointerventional procedures [39, 79].

In contrast, endovascular BCI is less suitable for patients with extensive cortical damage in target regions, such as large infarctions or hemorrhages involving the primary motor cortex, where viable neural signals are unlikely to be obtained [29, 85, 86]. It is also contraindicated in individuals with conditions that preclude safe endovascular intervention, including severe contrast allergy, uncontrolled coagulopathy, active systemic infection, or significant renal impairment limiting contrast use [35]. Moreover, anatomical vascular constraints, such as severe stenosis, occlusion, or excessive tortuosity of venous sinuses or access vessels, may prevent safe device deployment [35]. Finally, patients requiring extremely high‐resolution neural signals for complex motor control may not yet be ideal candidates, although ongoing advances in electrode design may address this limitation [29].

##### 2.1.5.2 BCI‐ESCS

For BCI‐ESCS, ideal candidates are patients with moderate‐to‐severe motor deficits in whom impaired voluntary drive through the corticospinal tract is the primary limitation, but spinal circuits below the lesion remain anatomically intact, a pattern commonly observed in hemispheric stroke [70]. Successful application also depends on the presence of detectable motor intention or motor imagery signals, as these are required to drive the closed‐loop stimulation system [51, 143]. Furthermore, intact spinal cord anatomy at the targeted stimulation level (cervical for upper limb function and thoracic or lumbar for lower limb function) is essential [138, 157, 217]. Patients who have reached a plateau with conventional rehabilitation may particularly benefit from this approach, as ESCS can enhance spinal excitability and promote activity‐dependent (Hebbian) plasticity when paired with intention‐driven stimulation [51, 136].

Patients who are less suitable candidates for BCI‐ESCS include those with significant structural pathology affecting the spinal cord at or below the intended stimulation level, such as concomitant SCI, severe spinal stenosis, tumors, syringomyelia, or advanced arachnoiditis, as these conditions may disrupt the integrity of spinal circuits required for effective neuromodulation [51, 70, 127]. Contraindications also extend to individuals with medical conditions that increase the risks of spinal surgery or implanted devices, including active spinal infection, severe osteoporosis with elevated fracture risk, bleeding disorders, or situations in which MRI is required but incompatible hardware would be implanted [100, 127, 142]. In addition, patients with a complete absence of detectable motor intention signals, such as those with severe locked‐in syndrome lacking reliable BCI control, are unlikely to benefit, given that BCI‐ESCS depends on cortical input to trigger stimulation [29, 51, 218]. Finally, patients whose primary impairment is severe spasticity without voluntary motor capacity may not be ideal candidates, as ESCS is primarily designed to facilitate volitional motor control rather than directly treat spasticity [99, 118, 219].

##### 2.1.5.3 BCI‐DBS

For BCI‐DBS, the most appropriate candidates are typically patients in the chronic phase of stroke (generally beyond 6–12 months) who exhibit moderate‐to‐severe upper limb motor deficits that have plateaued despite conventional rehabilitation, particularly in the presence of corticospinal tract damage such as lesions involving the internal capsule or corona radiata [69, 162, 167]. Target selection is closely associated with stroke pathology and underlying neuroanatomical pathways. For example, stimulation of the cerebellar DN may be beneficial in patients with cortical or subcortical lesions by enhancing dentatothalamocortical drive to the ipsilesional cortex [69, 177, 220], whereas targeting the motor thalamus (e.g., VIM/VOP nuclei) may directly facilitate corticospinal output via thalamocortical projections [168]. Other targets, including the STN, mesencephalic locomotor region, and hypothalamus, are under investigation for more specific functional impairments such as gait dysfunction or dystonia [169, 170, 171]. Suitable candidates must also be medically stable and able to tolerate stereotactic neurosurgery, including general anesthesia, and should retain sufficient connectivity between the stimulation target and downstream motor pathways to enable effective neuromodulation [69, 168, 177, 220].

Conversely, BCI‐DBS is less appropriate for patients with extensive encephalomalacia or marked brain atrophy involving the intended target or electrode trajectory, as these factors may increase surgical risk, particularly hemorrhage, and reduce therapeutic efficacy [69, 167]. Additional contraindications include uncontrolled seizures, severe cognitive impairment or dementia that limits cooperation or the ability to report adverse effects, active intracranial infection, significant psychiatric instability, and inability to adhere to long‐term device management requirements [69]. Patients in the acute phase of stroke (within approximately 6 months) are also generally not considered ideal candidates, as spontaneous recovery is still ongoing and the risk–benefit profile of invasive intervention is less favorable; accordingly, DBS is primarily explored in the chronic stage [69, 168]. Furthermore, the presence of MRI‐incompatible implants may complicate targeting and follow‐up imaging when MRI guidance is required, unless alternative strategies are available [69].

##### 2.1.5.4 General Considerations for All Modalities

Across all BCI‐based modalities, several general considerations are critical for patient selection. Adequate cognitive function is essential to ensure that patients can understand the therapeutic process, participate in BCI calibration and rehabilitation training, and provide informed consent. Psychological stability is equally important, as engagement and adherence are key determinants of therapeutic success. Furthermore, patient selection should be conducted within a multidisciplinary framework involving neurologists, neurosurgeons, neurorehabilitation specialists, neuropsychologists, and neuroradiologists, ensuring a comprehensive and individualized assessment of suitability for these advanced neurotechnological interventions.

### 2.2 Parkinson's Disease

PD is a progressive neurodegenerative disorder characterized by a broad spectrum of motor and non‐motor symptoms that increasingly impair functional independence [221]. The cardinal motor features, tremor, rigidity, bradykinesia, and postural instability, substantially limit activities of daily living, including self‐care, work‐related tasks, and leisure activities [222]. PD is now recognized as the fastest growing neurological disorder worldwide. Its prevalence more than doubled between 1990 and 2015, exceeding 6 million cases, and reached approximately 8.5 million globally by 2019 [223]. Projections suggest that this number may surpass 12 million by 2040 [224]. Pathophysiologically, PD is characterized by the degeneration of dopaminergic neurons in the substantia nigra pars compacta and the accumulation of α‐synuclein in the form of Lewy bodies. These changes disrupt the functional integrity of the cortico‐basal ganglia‐thalamocortical motor circuit [225]. At the electrophysiological level, this dysfunction is associated with abnormal neuronal synchronization, particularly within the STN. Excessive oscillatory activity in the β frequency band (13–30 Hz) has been strongly linked to key motor symptoms such as bradykinesia and rigidity [226, 227, 228]. Although standard treatments, including levodopa therapy and conventional continuous deep brain stimulation (cDBS), can effectively alleviate symptoms, they are limited by complications such as motor fluctuations, dyskinesia, and potential neural adaptation to chronic stimulation [229]. These limitations have driven a paradigm shift toward closed‐loop or adaptive neuromodulation strategies that dynamically adjust stimulation based on real‐time neural signals, aligning closely with BCI principles.

Central to this advancement is the development of DBS systems capable of both neural recording and stimulation. These systems enable real‐time monitoring of pathological β activity in the STN and allow stimulation parameters to be adjusted accordingly [18]. In adaptive DBS (aDBS), stimulation intensity is increased when β activity rises and reduced or suspended when β activity subsides [230]. This approach not only improves the precision of neuromodulation but also enhances therapeutic efficiency [231]. Clinical studies have demonstrated that aDBS can outperform conventional cDBS in both efficacy and efficiency. For example, Bocci et al. reported that, in eight patients with PD, 8‐h sessions of aDBS resulted in greater motor symptom improvement and lower energy consumption compared with cDBS [232]. Similarly, a longitudinal study by Caffi et al. described a patient undergoing 1 year of continuous aDBS, interspersed with brief periods of cDBS [233]. Remarkably, the patient was able to discontinue dopaminergic medication after 9 months of aDBS, and the therapy preserved physiologically relevant subthalamic β activity, contributing to superior clinical outcomes. Further support comes from a multicenter retrospective study demonstrating that aDBS provides better long‐term control of motor symptoms and allows for greater reductions in dopaminergic medication compared with cDBS [234]. In addition, a meta‐analysis by An et al. found that aDBS reduced total electrical energy delivery by approximately 55% relative to cDBS, while also suggesting improved overall motor outcomes [235].

In addition to aberrant neural activity in deep brain structures such as the STN to guide stimulation, alternative strategies have explored the use of cortical signals, particularly abnormal γ‐band activity, to control aDBS [185]. This approach spatially separates the sensing and stimulation sites, offering several advantages. By avoiding signal acquisition from the stimulation target itself, it reduces contamination from stimulation artifacts and overcomes the limited electrode contact selection inherent in systems that use the same lead for both recording and stimulation [182, 236, 237]. Swann et al. demonstrated that narrowband γ oscillations (60–90 Hz) in the motor cortex are strongly associated with DBS‐induced dyskinesia in patients with PD, with elevated γ activity serving as a reliable biomarker for dyskinetic states [238]. Building on this observation, they developed an aDBS system in which cortical γ activity, recorded via subdural ECoG, was used to dynamically modulate STN stimulation [185]. In this paradigm, stimulation intensity was reduced when γ activity increased and augmented when γ activity decreased. This cortical‐driven control strategy achieved clinical outcomes comparable to conventional continuous DBS while significantly reducing energy consumption by approximately 38%–45%. Importantly, STN β oscillations and cortical γ oscillations exhibit complementary physiological and pathological characteristics. β‐Band activity in the STN is closely linked to motor symptom severity and thus provides a direct and intuitive control signal for aDBS. However, its relatively low amplitude and sensitivity to voluntary movement present limitations. During normal movement, STN β power decreases physiologically, which may be misinterpreted by aDBS systems as a reduction in pathological activity, potentially leading to inappropriate reductions in stimulation and impaired motor performance [239]. In contrast, cortical narrowband γ oscillations are less influenced by voluntary movement and more specifically reflect dyskinesia‐related activity [240, 241]. These complementary features suggest that multimodal control strategies integrating both STN β and cortical γ signals may offer a more robust and physiologically informed framework for aDBS. Such approaches could enhance therapeutic precision, maximizing symptom control while minimizing stimulation‐induced side effects.

Beyond invasive neuromodulation, non‐invasive BCI approaches have also shown promising, albeit preliminary, clinical benefits for both motor and non‐motor symptoms of PD. For example, Turconi et al. reported that 15 sessions of motor imagery‐based EEG‐BCI training in three patients led to reductions in freezing of gait, increased α‐ and β‐band power, and improvements in attention and executive function [242]. Similarly, Lavermicocca et al. demonstrated that 24 sessions of EEG‐based neurofeedback training in 10 patients with mild cognitive impairment resulted in significant gains in attention, memory, reaction time, and overall cognitive efficiency [243]. Pettenuzzo et al. further showed that motor imagery‐driven BCI combined with an exoskeleton glove improved both cognitive and motor‐related functions in 12 patients, including enhancements in sustained attention, working memory, verbal fluency, and visuospatial abilities, with benefits persisting at 4‐week follow‐up [244]. In parallel, studies using functional MRI‐based BCI have demonstrated that patients can learn to modulate activity in motor‐related regions such as the supplementary motor area. Subramanian et al. reported a 37% increase in movement speed alongside improved clinical motor scores following such training [245], while Buyukturkoglu et al. similarly showed that voluntary modulation of supplementary motor area activity could influence motor outcomes [246].

The integration of BCI technology with neural implants offers substantial promise for the management of PD. aDBS has already demonstrated notable advantages in both clinical efficacy and energy efficiency, while neurofeedback and neuroimaging‐guided BCI approaches provide non‐invasive means of modulating motor circuits. However, despite encouraging preliminary findings in both motor and cognitive domains, the current evidence base remains limited by small sample sizes and heterogeneous methodologies across pilot studies. In addition, previous literature is disproportionately focused on invasive aDBS, with comparatively less attention given to non‐invasive BCI paradigms [222]. To advance the field, well‐designed, large‐scale clinical trials are needed to establish strong evidence for the safety and efficacy of BCI‐based interventions in PD. From a technical perspective, each approach presents distinct challenges. Non‐invasive BCI methods are often constrained by signal noise and artifacts, as well as trade‐offs between electrode comfort and signal fidelity. In contrast, invasive techniques, while offering higher precision, are associated with surgical risks and significant economic costs. Against this backdrop, emerging non‐invasive DBS technologies represent a promising direction for overcoming these limitations. One such innovation is tTIS, a non‐invasive technique that applies two or more high‐frequency electrical currents (> 1 kHz) at the scalp. These currents interact to generate a low‐frequency envelope within deep brain regions, with the modulation frequency determined by the difference between the applied signals. This approach enables targeted modulation of deep neural structures without the need for surgical implantation [49]. Early studies have demonstrated its feasibility in PD. For example, Yang et al. applied 20 min of tTIS targeting thalamic subnuclei in eight patients, reporting good tolerability and potential improvements in motor symptoms [203]. Similarly, Lamoš et al. delivered 130 Hz stimulation to the STN using tTIS and successfully suppressed pathological β oscillations [247]. Other non‐invasive approaches have also shown promise. Darmani et al. employed transcranial ultrasound stimulation to target the basal ganglia and demonstrated modulation of β‐band activity using 10 Hz stimulation, further supporting the feasibility of non‐invasive deep brain modulation [248]. Despite these encouraging early results, these technologies remain in the experimental stage, and rigorous clinical trials are still needed to confirm their therapeutic value and long‐term safety.

### 2.3 Epilepsy

Epilepsy is a chronic neurological disorder characterized by abnormal, hypersynchronous electrical activity in the brain, leading to a wide range of clinical manifestations, including impaired consciousness, convulsive seizures, autonomic disturbances, and subtle cognitive dysfunction [249, 250]. Although antiepileptic drugs remain the primary treatment, nearly one‐third of patients develop drug‐resistant epilepsy (DRE), in which seizures persist despite appropriate pharmacological therapy [10]. For patients with DRE who are not suitable candidates for resective surgery, neuromodulation has emerged as an important therapeutic option. Conventional open‐loop approaches, such as VNS and DBS, deliver stimulation according to fixed parameters without accounting for real‐time brain activity. As a result, their efficacy may be limited, and unnecessary stimulation can lead to adverse effects [251]. In contrast, advances in BCI‐inspired technologies have enabled the development of closed‐loop responsive neurostimulation (RNS) systems. These systems integrate high‐resolution neural recording with intelligent, feedback‐driven stimulation strategies [19]. Clinically, they serve two key functions: first, they detect or predict abnormal electrical activity associated with seizures, aiding diagnosis and treatment planning; second, they deliver targeted stimulation in response to these signals to prevent or attenuate seizure onset. Unlike open‐loop systems, RNS operates in an on‐demand manner, activating only when pathological activity is detected. This approach reduces unnecessary stimulation and may minimize long‐term side effects while improving therapeutic precision [48]. In the following section, we review the application of these BCI‐based closed‐loop neuromodulation strategies in the management of epilepsy.

The effectiveness of closed‐loop neuromodulation in epilepsy depends critically on the performance of real‐time decoding algorithms for seizure detection and prediction [252, 253]. EEG, including both intracranial (iEEG) and scalp recordings, remains the primary modality for capturing pathological neural dynamics associated with epileptic activity [254, 255]. To interpret these signals, a wide range of machine learning and deep learning approaches have been developed, typically involving feature extraction followed by automated classification. Commonly analyzed features include frequency band power, spatial energy distribution, and measures of network synchronization [256, 257]. These features are then processed using advanced algorithms, such as support vector machines (SVM) [258], convolutional neural networks (CNN) [259], and long short‐term memory (LSTM) networks [260], to identify transitions into ictal states with high accuracy. Recent studies have demonstrated the potential of these approaches. For example, Burrello et al. developed *Laelaps*, a brain‐inspired algorithm based on symbolic dynamics and hyperdimensional computing, capable of performing efficient end‐to‐end binary operations [261]. When applied to 2656 h of iEEG recordings from 18 patients with DRE, the system detected 116 seizures with zero false positives, achieving an average sensitivity of 85.5% and a mean detection latency of 18.9 s. Similarly, Marleen et al. applied CNN and LSTM architectures to scalp EEG data from patients with focal epilepsy, achieving an area under the ROC curve (AUC) of 0.94, with high specificity (98.0%), albeit moderate sensitivity (47.4%) [262]. Zhong et al. further demonstrated the utility of recurrent neural networks, reporting average accuracies of 73.33% and 81.33% using LSTM and bidirectional LSTM models, respectively [263]. Collectively, these studies provide a strong foundation for real‐time seizure detection and have significantly advanced the development of closed‐loop RNS systems.

At present, open‐loop neuromodulation strategies in epilepsy are primarily represented by VNS and DBS. In VNS, a pulse generator is surgically implanted in the chest, with electrodes wrapped around the left vagus nerve in the neck [264]. Although its precise mechanisms are not fully understood, VNS is thought to modulate neuronal activity in the nucleus of the solitary tract via afferent vagal fibers, with downstream effects on key brain regions including the locus coeruleus, dorsal raphe nucleus, hippocampus, and amygdala [265, 266]. This widespread network modulation is believed to desynchronize epileptiform activity, particularly in higher frequency bands, and alter thalamocortical dynamics [48]. Clinically, VNS has been shown to reduce seizure frequency by more than 50% in a substantial proportion of patients after 1 year of treatment [267, 268, 269]. In open‐loop DBS, electrodes are stereotactically implanted into specific brain targets, most commonly the anterior nucleus of the thalamus (ANT) and the centromedian nucleus (CMN) [270]. The ANT is extensively connected within the limbic system, including projections to the hippocampus, cingulate cortex, and parietal regions, making it a key node in temporal lobe and limbic epilepsy networks [270, 271, 272]. Its efficacy has been validated in patients with focal and secondary generalized DRE [273]. Notably, the landmark SANTE trial, a large, double‐blind randomized controlled study, reported a median seizure reduction of 69% at 5 years, with 68% of patients achieving at least a 50% reduction in seizure frequency [274]. The CMN, part of the intralaminar thalamic nuclei, has widespread connections to the striatum, reticular formation, and sensorimotor cortex, positioning it as an important regulator within cortico‐thalamo‐cortical circuits [270, 275]. CMN‐DBS has shown particular benefit in generalized epilepsies, including Lennox–Gastaut syndrome and idiopathic generalized DRE [276]. Evidence from multiple clinical studies and smaller randomized trials further supports its therapeutic potential in these populations [277, 278, 279]. While these open‐loop approaches have demonstrated substantial efficacy, they do not account for the dynamic and unpredictable nature of seizure activity. As a result, closed‐loop, on‐demand neuromodulation strategies, exemplified by RNS systems, have emerged as a more targeted alternative. By delivering stimulation only in response to detected or predicted seizure activity, these systems aim to improve therapeutic precision while minimizing unnecessary stimulation and associated adverse effects [183, 280, 281, 282].

The RNS system is an FDA‐approved therapy for DRE that utilizes real‐time monitoring and automated feedback to control seizure activity [48]. It continuously records cerebral electrical signals through implanted electrodes or external sensors and, upon detecting electrographic signatures that precede seizure onset, delivers pre‐programmed electrical stimulation to interrupt or mitigate the developing ictal event [19]. A landmark multicenter, double‐blind, randomized controlled trial conducted by Morrell et al. provided Class I evidence for the therapeutic benefit of RNS [283]. In this study, electrodes capable of both recording and stimulation were implanted at subdural or deep cortical seizure foci in patients with DRE. During a 12‐week blinded phase, participants were randomized to receive either active responsive stimulation or sham treatment, followed by an 84‐week open‐label extension. At the end of the blinded phase, patients receiving active RNS exhibited a significantly greater reduction in seizure frequency compared with the control group (37.9% vs. 17.3%). This benefit persisted during the open‐label phase, with additional improvements observed after activation of the device in the former sham group. Over a longer follow‐up, median seizure reductions reached 44% at 1 year and 53% at 2 years, indicating sustained therapeutic effects. Subsequent studies have explored alternative targets and stimulation strategies. For example, Sisterson et al. investigated RNS targeting the CMN of the thalamus in patients with idiopathic generalized DRE [53]. By implanting electrodes bilaterally in the CMN and triggering stimulation in response to intrathalamic epileptiform activity, they reported substantial seizure reductions ranging from 75% to 99% across four patients. These improvements were accompanied by shorter and less severe seizures, as well as meaningful gains in quality of life, all of which were maintained over at least 2 years of follow‐up. Closed‐loop concepts have also been applied to VNS. Winston et al. evaluated a system that delivers stimulation in response to rapid increases in heart rate associated with seizure onset [284]. This approach enabled stimulation within 2 min of seizure detection and resulted in greater seizure reduction and improved clinical outcomes over 9 months compared with conventional open‐loop VNS. In addition to ictal intervention, there is emerging interest in interictal stimulation paradigms. Anderson et al. applied closed‐loop RNS during periods of low epileptiform activity in a cohort of 40 patients [285]. Their findings suggest that such stimulation may induce gradual, long‐term modifications in brain network dynamics, potentially by rebalancing excitatory and inhibitory activity, thereby contributing to improved clinical outcomes. Despite these promising results, broader analyses and meta‐analyses indicate that the long‐term efficacy of RNS may not differ substantially from that achieved with established open‐loop modalities such as VNS and DBS [286, 287]. This suggests that the clinical advantage of closed‐loop over open‐loop neuromodulation may be more modest than initially anticipated.

Ultrasound has recently emerged as a promising non‐invasive neuromodulation approach for epilepsy, with anti‐epileptic effects demonstrated across multiple experimental systems, including human brain tissue, rodent models, non‐human primates, and early‐stage clinical studies [50, 288, 289, 290, 291, 292, 293, 294]. Lin et al. employed low‐intensity pulsed ultrasound, characterized by high spatial precision and deep tissue penetration, to target epileptogenic regions in a penicillin‐induced monkey model [288]. Following a 30‐min stimulation session, both the total number of seizures over a 16‐h period (107.7 vs. 66.0) and the hourly seizure frequency (15.6 vs. 9.6) were significantly reduced compared with controls. In addition, studies using ex vivo brain tissue from patients with epilepsy have shown that ultrasound can suppress epileptiform activity by enhancing inhibitory synaptic input, achieving efficacy rates exceeding 65%. Early clinical investigations further support its feasibility. Lee et al. applied a 10‐min ultrasound stimulation protocol to six patients with DRE, in whom epileptogenic zones had been precisely identified using stereoelectroencephalography (SEEG) [291]. During stimulation, significant changes in spectral power were observed at the targeted electrodes. Clinically, two patients exhibited a reduction in seizure frequency during a 3‐day follow‐up period. In terms of safety, one patient reported a transient sensation of scalp warmth, while another experienced temporary naming and memory deficits that resolved completely within 3 weeks. Similarly, Stern et al. conducted preoperative ultrasound stimulation in eight patients with refractory temporal lobe epilepsy undergoing anterior temporal lobectomy [295]. Histological analysis of resected tissue revealed no evidence of structural damage, and neuropsychological assessments showed no significant postoperative changes, providing preliminary support for the safety of this approach. Building on these findings, closed‐loop ultrasound neuromodulation strategies have begun to emerge. Yang et al. developed a system in a mouse model of temporal lobe epilepsy in which electrodes implanted in the hippocampal CA1 region continuously monitored local field potentials to detect seizures in real time, triggering targeted ultrasound stimulation of the hippocampus [296]. Compared with sham stimulation, this closed‐loop approach significantly delayed seizure onset and reduced seizure duration. Similarly, Zhong et al. combined EEG‐based detection with LSTM and bidirectional LSTM algorithms to identify ictal events and initiate a 10‐min ultrasound intervention targeting the hippocampus [263]. This strategy reduced EEG power spectral density, shortened seizure duration, and improved survival rates in early‐stage epileptic mice from 67.57% to 88.89% [297]. In a pentylenetetrazol‐induced rat model of epilepsy, this system rapidly suppressed seizures by selectively activating mechanosensitive neurons in the nodose ganglion and modulating pathological activity in the hippocampus and amygdala. Notably, in acute experiments, this aBCI approach demonstrated superior anti‐epileptic efficacy compared with conventional vagus nerve stimulation. By markedly reducing the need for surgical intervention compared with traditional approaches such as DBS and vagus nerve stimulation, ultrasound offers a more accessible and patient‐friendly alternative. Nevertheless, most current evidence remains preclinical, and further well‐designed clinical trials are essential to establish its safety, efficacy, and optimal stimulation parameters for individualized treatment.

## 3 Cognitive and Mental Disorders

Cognitive and psychiatric disorders, such as AD and depression, pose a distinct set of challenges compared with motor disorders. Rather than arising from focal lesions or single abnormal rhythms, these conditions are characterized by gradual, progressive, and often widespread disruptions in neural networks responsible for memory, emotion, and executive function. As a result, the application of BCI technologies in this domain follows a dual and complementary strategy. First, BCI systems can serve as sensitive tools for diagnosis and longitudinal monitoring. By using advanced signal analysis of modalities such as EEG, fNIRS, and SEEG, it is possible to detect subtle, disease‐specific neural signatures, for example, altered neurovascular coupling in AD or state‐dependent changes in amygdala γ activity in depression. These signals provide a means for early detection and objective tracking of disease progression. Second, these neural signatures can be used as feedback signals to guide therapeutic interventions. These range from non‐invasive approaches, such as neurofeedback training aimed at helping patients self‐regulate abnormal brain activity, to invasive strategies, including closed‐loop DBS that delivers targeted, state‐dependent neuromodulation. This section examines these principles in two representative conditions. Section 3.1 focuses on AD, with an emphasis on early diagnosis and cognitive training, while Section 3.2 explores depression, where the development of personalized, closed‐loop DBS represents an emerging frontier in psychiatric treatment.

### 3.1 Alzheimer's Disease

AD is a progressive neurodegenerative disorder characterized by the accumulation of extracellular β‐amyloid (Aβ) plaques and intracellular hyperphosphorylated tau protein, leading to synaptic dysfunction, neuronal loss, and ultimately cognitive decline [298]. With global population aging, the prevalence of AD continues to rise, placing a growing burden on patients, caregivers, and healthcare systems [299]. Recent estimates indicate that approximately 55 million individuals worldwide were living with AD in 2023, with projections suggesting this number could reach nearly 150 million by 2050 [300]. Current treatment strategies, including cholinesterase inhibitors and other symptomatic therapies, provide only modest benefits and do not halt disease progression [223]. Emerging evidence suggests that BCI‐based approaches may offer new opportunities, both for early detection of AD through sensitive neural biomarkers and for slowing cognitive decline through targeted, activity‐dependent training interventions.

The pathological processes underlying AD are thought to begin many years (often decades) before the onset of clinical symptoms, highlighting the critical importance of early detection and intervention. However, current diagnostic approaches rely largely on clinical presentation and conventional neuroimaging, both of which are limited by delayed detection, variability in biomarker expression, and insufficient sensitivity to subtle early‐stage changes [301]. As a result, diagnosis is frequently made only after significant neurodegeneration has already occurred. BCI‐based approaches that analyze brain signals have shown considerable promise for identifying early AD‐related alterations in neural activity [302, 303]. Mild cognitive impairment (MCI), widely regarded as a transitional stage between normal aging and dementia, represents a key target for early detection. Timely identification of MCI could enable early therapeutic intervention and potentially delay or prevent progression to AD, yet it often remains underdiagnosed using conventional clinical methods. Several studies have explored the use of BCI‐driven signal analysis for this purpose. Fukushima et al. developed a character‐based BCI paradigm that recorded event‐related P300 potentials while participants attended to visual stimuli, extracting key features to distinguish between MCI and AD with an accuracy of 80% [304]. Hojjati et al. combined structural MRI‐derived gray matter volume with resting‐state functional connectivity features and applied a SVM model, achieving a prediction accuracy of 97% for the conversion from MCI to AD [305]. Similarly, Ieracitano et al. transformed EEG power spectral data into two‐dimensional spectrograms and applied a CNN, achieving an average pairwise classification accuracy of 89.8% and an overall three‐class accuracy of 83.3% in distinguishing normal aging, MCI, and AD [306]. fNIRS, which measures hemodynamic responses associated with neural activity, has also emerged as a promising tool for early AD detection. A systematic review by Li et al. reported that fNIRS can identify reduced activation in frontal, temporal, and parietal regions in both MCI and AD patients during resting‐state conditions, along with decreased tissue oxygenation and weakened functional connectivity. During cognitive tasks, these patients exhibit attenuated activation across multiple regions, reduced connectivity strength, and diminished signal complexity. Notably, machine learning models applied to fNIRS data have achieved classification accuracies approaching 90% [307]. The integration of multimodal BCI techniques may further enhance diagnostic performance. Chiarelli et al. demonstrated that while EEG and fNIRS metrics alone were insufficient to distinguish early‐stage AD from healthy controls, their combined analysis revealed significantly impaired neurovascular coupling in AD patients, achieving an AUC of 0.905 for classification [308]. Beyond conventional electrophysiological and hemodynamic signals, novel approaches are also being explored. Sefati et al. investigated hippocampal ultra‐weak photon emission (UPE) as a potential biomarker of AD progression in animal models, and found that UPE intensity was significantly elevated in AD rats and reduced following treatment with donepezil [309]. These findings raise the possibility of developing minimally invasive BCI‐based photonic sensors capable of monitoring hippocampal activity through the temporal bone, offering a potential new avenue for screening, diagnosis, and longitudinal monitoring of AD progression [18].

BCI‐based therapeutic strategies for AD have shown encouraging potential, with the aim of restoring cognitive functions such as memory and attention while possibly slowing disease progression. One of the most accessible approaches involves combining BCI with neurofeedback training (NFB). In this paradigm, patients learn to modulate their own brain activity through real‐time feedback. For example, Galvin‐McLaughlin et al. conducted a 6‐week BCI‐NFB program in patients with mild AD, incorporating tasks such as identifying target letters, copying words, and selectively crossing out stimuli. Their preliminary findings suggested that this approach is feasible in AD, although the small sample size necessitates further validation [310]. More targeted neurofeedback strategies have also been explored. Lin et al. focused on frontal γ‐band coherence, which is typically reduced in patients with AD and MCI. By translating this neural feature into a feedback signal using EEG, they enabled patients to directly regulate their own frontal γ activity. Results from a double‐blind, placebo‐controlled trial demonstrated that patients receiving neurofeedback training showed significant improvements in frontal γ coherence [311]. However, given the progressive nature of AD, such active, training‐based approaches may be less suitable for individuals in moderate to advanced stages, where cognitive impairment limits the ability to engage with BCI systems. In these cases, neuromodulation‐based interventions may be more appropriate [312]. Non‐invasive brain stimulation techniques have shown promising results in this regard. For instance, Im et al. applied tDCS to the dorsolateral prefrontal cortex over a 6‐month period, reporting improvements in global cognition and language function, along with a partial preservation of executive abilities [313]. Similarly, Jiang et al. delivered TMS to the bilateral prefrontal cortex over 4 weeks using either high‐frequency (10 Hz) or low‐frequency (2 Hz) protocols. Both approaches improved behavioral and functional outcomes, although high‐frequency stimulation appeared to confer greater cognitive benefits [314]. At the invasive end of the spectrum, DBS has also been investigated as a potential therapeutic option for AD. The fornix, a key white matter tract connecting the hippocampus with other limbic structures and a central component of the Papez circuit, is the most commonly targeted region [315]. A multicenter study by Germann et al. reported that patients receiving fornix‐DBS exhibited reduced hippocampal atrophy, decreased amyloid‐β PET signal, and increased cerebrospinal fluid Aβ/total tau ratios, suggesting a potential disease‐modifying effect [316]. These findings are broadly consistent with observations from pharmacological studies of recently approved agents such as aducanumab and lecanemab, which also target amyloid pathology [317, 318]. In addition, Howard et al. found that fornix‐DBS may increase hippocampal volume in older patients with AD, potentially contributing to cognitive improvements [319].

Despite these advances, several important challenges remain. In particular, patients with advanced AD often experience profound cognitive decline, which may limit their ability to interact with BCI systems or voluntarily modulate neural activity. As a result, extending the benefits of BCI‐based diagnosis and therapy to moderate and advanced stages of the disease remains a key area for future research.

### 3.2 Depression

Depression is a common and disabling psychiatric disorder, and a substantial proportion of patients derive limited benefit from conventional treatments such as pharmacotherapy and psychotherapy. MDD, the most severe form, can progress to treatment‐resistant depression (TRD), which is defined by a lack of response to standard therapeutic interventions [11]. DBS has emerged as a potential alternative, with several early studies reporting encouraging outcomes [320, 321, 322, 323]. For instance, a randomized clinical trial by Bergfeld et al. implanted DBS electrodes in the bilateral ventral anterior limb of the internal capsule in patients with TRD, resulting in significant symptom improvement in 10 of 25 participants, with good overall tolerability [321]. Similarly, Posse et al. targeted the subcallosal cingulate (SCC), reporting response rates exceeding 80% after 6 months of open‐label stimulation [322]. Despite these promising findings, two subsequent large clinical trials were terminated early after interim analyses failed to demonstrate significant differences between active stimulation and control groups [324, 325]. This substantial variability in efficacy arises from the heterogeneity of individual responses among depression patients; different patients may require stimulation of distinct targets with personalized parameters. Furthermore, stimulation should ideally be triggered selectively based on changes in the patient's physiological state, that is, only when pathological states are detected [326]. Closed‐loop neuromodulation offers several potential advantages in this regard. By tailoring stimulation to real‐time neural or physiological signals, it may reduce neural adaptation, minimize side effects, and improve energy efficiency. Importantly, studies have shown that the effects of stimulating a given brain region can depend on the patient's emotional or neural state at the time of stimulation. In some cases, stimulation of the same target may lead to either symptom improvement or worsening, depending on the pre‐existing emotional conditions [327]. Taken together, these findings highlight the need for closed‐loop DBS systems capable of accurately identifying symptom‐relevant brain states and dynamically adjusting stimulation accordingly.

At present, SEEG is primarily used to identify patient‐specific biomarkers that can guide closed‐loop neuromodulation in TRD [328]. Using machine learning approaches, they identified increased γ‐band activity in the bilateral amygdala as a neural correlate of high symptom severity. Based on this finding, the authors implanted recording electrodes in the amygdala and stimulation electrodes in the ventral capsule/ventral striatum (VC/VS), enabling a closed‐loop system in which amygdala γ activity triggered targeted stimulation. Remarkably, the patient required no more than 30 min of stimulation per day to achieve rapid and sustained symptom improvement. Over several months, her Montgomery‐Åsberg Depression Rating Scale score decreased from 36 to below 10, consistent with clinical remission. Although this study highlights the promise of personalized, closed‐loop DBS, its generalizability remains uncertain given that it was conducted in a single patient, and it is unclear whether the same biomarker applies across individuals. An alternative closed‐loop strategy was explored by Sheth et al., who adopted a more comprehensive mapping approach [329]. In a 37‐year‐old male patient with TRD, DBS leads were implanted in both the bilateral SCC and VC/VS, along with 10 SEEG electrodes placed in depression‐related circuits. Over a 9‐day period, the patient performed behavioral tasks designed to probe emotional and cognitive states while receiving stimulation across a wide range of parameters. SEEG recordings were used to identify neural markers associated with positive and negative affective states and to evaluate how different stimulation settings modulated these markers. This individualized mapping approach enabled the selection of optimal stimulation parameters tailored to the patient. After removal of the SEEG electrodes, continuous DBS therapy was delivered using these parameters, resulting in significant symptom improvement over an 8‐month follow‐up period. Notably, symptom worsening occurred when stimulation was discontinued and improved again upon reinitiation, supporting a causal therapeutic effect [330]. Similarly, non‐invasive closed‐loop neuromodulation strategies are also being explored. Increased α‐band activity (8–12 Hz), particularly in the prefrontal cortex, has been associated with depressive symptomatology. Building on this observation, Schwippel et al. developed an EEG‐guided closed‐loop system in which individualized alpha power fluctuations were used to induce tACS over bifrontal regions [331]. After five consecutive days of treatment, patients with MDD showed significant reductions in depressive symptoms along with improvements in quality of life, and 80% achieved remission within 2 weeks. Although these findings demonstrate the feasibility of closed‐loop tACS, further research is required to determine its effectiveness in patients with more severe or treatment‐resistant forms of depression.

## 4 Other Neurological and Psychiatric Disorders

BCI technology has been widely explored in the five major conditions discussed above, namely, stroke, PD, epilepsy, AD, and depression, where a substantial body of research has accumulated. In contrast, its application to other neurological and psychiatric disorders remains at a relatively early stage, particularly with respect to emerging neural recording and stimulation modalities. Nevertheless, many of the core strategies developed in these well‐studied conditions are readily transferable. These include continuous brain‐state monitoring and early warning of pathological events (e.g. seizure prediction), event‐triggered neuromodulation based on disease‐specific neural signatures (as exemplified by closed‐loop DBS in PD), and the restoration or substitution of impaired neurological functions [18]. In individuals with motor impairments arising from a range of neurological conditions, BCI systems have demonstrated value in two principal domains. First, they can support motor recovery through rehabilitation‐focused interventions. Second, they can enable patients to interact with their environment by controlling external devices, thereby compensating for lost motor function [332]. For example, a sensorimotor demultiplexing BCI has been tested in patients with SCI, where residual, subperceptual tactile signals in M1 were decoded alongside motor intentions. This approach enabled intracortically controlled, closed‐loop sensory feedback, nearly restoring the ability to perceive object contact and significantly improving overall sensorimotor performance [333]. This system achieved decoding speeds of up to 62 words per minute with an accuracy of 76.2% across a large vocabulary of 125,000 words, representing a major advance in communication restoration for patients with severe paralysis [334]. In patients with disorders of consciousness, BCI‐based paradigms have been used to detect residual awareness through tactile, auditory, visual, and multimodal stimulation approaches [1, 335, 336]. Qin et al. utilized quantitative EEG to predict 3‐month outcomes in comatose patients in a neurointensive care setting, offering a means of monitoring consciousness levels and informing clinical decision‐making [337]. Pan et al. developed a hybrid BCI paradigm that integrates auditory P300 responses with visual steady‐state visual evoked potentials, demonstrating its feasibility in both healthy individuals and patients with disorders of consciousness [338]. In their study, four healthy participants, one patient in a vegetative state, and one in a minimally conscious state were all able to successfully complete the intended tasks. These findings highlight the potential of hybrid BCI systems as practical bedside tools for assessing residual awareness and monitoring levels of consciousness. Beyond disorders of consciousness, BCI technologies are increasingly being explored in psychiatric conditions, where they enable real‐time detection of emotional or pathological brain states and can trigger targeted interventions. These interventions include neurofeedback approaches aimed at improving patients’ ability to self‐regulate symptoms, as well as neuromodulation techniques [164]. Among these, closed‐loop neural stimulation strategies, encompassing invasive approaches such as DBS and VNS, as well as emerging non‐invasive methods such as ultrasound and temporal interference stimulation, are particularly promising. For example, Nho et al. utilized DBS electrodes to monitor low‐frequency oscillatory activity in the nucleus accumbens and ventral pallidum, key components of the ventral striatum, in patients with OCD [339]. By detecting symptom‐related neural signatures, the system triggered targeted DBS in a closed‐loop manner. This approach led to a rapid 37.5% reduction in Yale‐Brown Obsessive Compulsive Scale scores within 24 h, suggesting the fast‐acting nature of closed‐loop neuromodulation. Over a 2‐year follow‐up, symptom severity continued to decline, ultimately reaching a 50% reduction, suggesting sustained therapeutic benefit. Similarly, Shivacharan et al. investigated a closed‐loop DBS strategy for binge eating disorder, in which increased low‐frequency (δ‐band) activity in the nucleus accumbens was used as a trigger for stimulation [340]. After 6 months of treatment, two patients exhibited improved control over food intake along with meaningful weight loss. In another study, Gill et al. applied a closed‐loop DBS approach in patients with post‐traumatic stress disorder (PTSD), using elevated θ‐band (5–9 Hz) activity in the amygdala as a trigger for stimulation [341]. After 1 year, patients showed significant reductions in PTSD symptoms, accompanied by decreased pathological amygdala θ activity. In addition, Chen et al. have highlighted the broader potential of integrating advanced neural sensing and stimulation capabilities for the treatment of substance use disorders, further extending the scope of BCI‐based interventions [27]. Although these studies remain preliminary and often involve small cohorts, they collectively demonstrate the versatility and promise of BCI technologies across a wide range of neurological and psychiatric conditions. Continued advances in minimally invasive and non‐invasive neural recording and stimulation techniques are likely to accelerate progress in this field, paving the way for more effective and accessible BCI‐based therapies. Although these studies remain preliminary and often involve small cohorts, they collectively demonstrate the versatility and promise of BCI technologies across a wide range of neurological and psychiatric conditions. Continued advances in minimally invasive and non‐invasive neural recording and stimulation techniques are likely to accelerate progress in this field, paving the way for more effective and accessible BCI‐based therapies.

## 5 Conclusion and Prospects

The integration of advanced neural recording and neurostimulation technologies within BCI frameworks is reshaping the landscape of neurorehabilitation. Rather than relying on passive, task‐based training alone, emerging BCI approaches enable active, precise, and closed‐loop modulation of neural circuits. In this review, we have highlighted three particularly promising strategies, namely, endovascular BCI, BCI‐ESCS, and BCI‐DBS, with a primary focus on post‐stroke motor recovery. Endovascular BCI provides a minimally invasive route for high‐quality neural signal acquisition, BCI‐ESCS facilitates brain‐controlled spinal stimulation to restore voluntary movement, and BCI‐DBS utilizes cortical or biomarker signals to dynamically regulate deep brain circuits. Early evidence across these modalities suggests not only immediate assistive benefits but also the potential to drive sustained, plasticity‐dependent functional recovery. Importantly, the underlying principles are not limited to stroke but are broadly applicable to a range of neurological and psychiatric disorders, including PD, epilepsy, AD, and depression, where BCI systems enable real‐time detection and targeted intervention for abnormal brain states.

Despite encouraging progress in both preclinical and early clinical studies, several challenges must be addressed before these technologies can be widely adopted in routine care. From a technical perspective, further improvements are needed in the spatial resolution and long‐term stability of neural recording devices, particularly for endovascular systems, as well as in the precision of stimulation, such as achieving fine motor control at the level of individual digits in spinal stimulation paradigms. In addition, robust and generalizable real‐time decoding algorithms are essential for reliably interpreting diverse neural biomarkers. From a clinical standpoint, much of the current evidence is derived from small‐scale, open‐label feasibility studies. Large, well‐designed randomized controlled trials are urgently needed to establish efficacy, refine stimulation protocols, and identify the patient populations most likely to benefit from each approach. Moreover, the invasive nature of many existing systems highlights the importance of continued innovation toward less invasive alternatives, including advanced endovascular platforms, transcutaneous spinal stimulation, and emerging non‐invasive DBS techniques such as temporal interference and FUS.

Looking ahead, the future of BCI‐based therapy will likely be defined by greater integration and personalization. Combining multiple modalities, for example, using endovascular recording to guide spinal or DBS, may enable multi‐level, closed‐loop systems capable of more comprehensive neural modulation. At the same time, advances in artificial intelligence, biomaterials, and wireless technologies are expected to support the development of more adaptive, durable, and user‐friendly devices. Ultimately, achieving truly personalized neurorehabilitation will require patient‐specific models for target selection and stimulation optimization, along with BCI systems that can continuously adapt to changes in neural activity and functional recovery over time.

Alongside the technical and clinical challenges, the successful translation and broader societal adoption of BCI technologies require careful consideration of their ethical and social implications. Foremost among these is the issue of privacy and data security. Neural recordings constitute a highly sensitive form of personal data, with the potential to reveal aspects of cognition, intention, and emotional state [342]. The protection of such sensitive neural data is therefore critical and necessitates comprehensive security measures, including encrypted wireless transmission, secure data storage, rigorous access control protocols, and the implementation of transparent data governance frameworks co‐designed with patients, clinicians, ethicists, and policymakers [343]. Equally important is ensuring that patients retain full autonomy over how their neural data are collected, stored, and shared. Another key consideration is the psychological and psychosocial impact of long‐term BCI use. Although these technologies provide meaningful opportunities for functional recovery, they may also introduce new challenges. Some patients may experience shifts in body image, sense of identity, or personal agency, for example, questioning whether a movement originates from themselves or the device [344]. Dependence on complex implanted systems may also generate anxiety related to device reliability, maintenance, or potential failure [345]. In addition, managing expectations is critical. Overly optimistic perceptions of BCI as a “cure” may lead to disappointment if outcomes are more modest, potentially affecting mental well‐being and engagement with rehabilitation [346, 347]. For these reasons, ongoing psychological support and counseling should be integrated into BCI treatment programs.

Equitable access represents a further major challenge. The high costs associated with device development, surgical implantation, maintenance, and long‐term rehabilitation raise concerns about the emergence of a “neurotechnology divide,” in which only a limited subset of patients can access these advanced therapies [348]. Addressing this issue will require coordinated efforts across healthcare systems, insurers, regulatory agencies, and broader society. Potential strategies include developing sustainable funding models, reducing costs through technological innovation and streamlined clinical workflows, and advocating for policies that prioritize access based on medical need rather than socioeconomic status [349]. Importantly, addressing these ethical and social dimensions is not optional but fundamental to the responsible development and deployment of BCI technologies. Significant engagement with diverse stakeholders, including patients, caregivers, clinicians, engineers, ethicists, policymakers, and the broader public, will be critical for addressing these complex challenges and promoting the equitable and responsible distribution of the benefits of these neurotechnological innovations.

In summary, BCI‐based neural recording and stimulation technologies offer unprecedented opportunities to restore function and improve quality of life in individuals with neurological and psychiatric disorders. Realizing this potential will require not only continued multidisciplinary innovation to overcome technical and clinical barriers but also a sustained commitment to ethical principles, ensuring that these transformative advances benefit society as a whole.

## Author Contributions

D.C. conceived the idea for the article. Y.X. wrote the first version of the manuscript, with constructive input from Q.Y., Pe.Z., J.S., S.L., Y.S., Z.Z., and Pi.Z., under supervision from Z.T. Y.X. and D.C. prepared the display items. D.C., Y.T., Pi.Z., and Z.T. provided proofreading and input on later versions of the manuscript. All authors approved the final version of the article.

## Funding

This work was supported by the Major Program (JD) of Hubei Province (2023BAA005), the National Natural Science Foundation of China (92148206), the Key Research and Development Program of Wuhan (2024020702030123), Huazhong University of Science and Technology (2024JCYJ044), the Fundamental Research Funds for the Central Universities (YCJJ20251401), and the Research Fund of Tongji Hospital (2022ZHFY01, AI2024B03).

## Ethics Statement

The authors have nothing to report.

## Conflicts of Interest

The authors declare no conflicts of interest.

## Data Availability

The authors have nothing to report.

## References

[1] H. Zhang , L. Jiao , S. Yang , et al., “Brain‐Computer Interfaces: The Innovative Key to Unlocking Neurological Conditions,” International Journal of Surgery 110, no. 9 (2024): 5745–5762. 39166947 10.1097/JS9.0000000000002022 PMC11392146

[2] A. S. Widge , K. K. Ellard , A. C. Paulk , et al., “Treating Refractory Mental Illness With Closed‐Loop Brain Stimulation: Progress Towards a Patient‐Specific Transdiagnostic Approach,” Experimental Neurology 287, no. pt. 4 (2017): 461–472. 27485972 10.1016/j.expneurol.2016.07.021

[3] X. Chai , T. Cao , Q. He , et al., “Brain‐Computer Interface Digital Prescription for Neurological Disorders,” CNS Neuroscience & Therapeutics 30, no. 2 (2024): e14615. 38358054 10.1111/cns.14615 PMC10867871

[4] P. Cuijpers , M. Sijbrandij , S. L. Koole , G. Andersson , A. T. Beekman , and C. F. Reynolds , “The Efficacy of Psychotherapy and Pharmacotherapy in Treating Depressive and Anxiety Disorders: A Meta‐Analysis of Direct Comparisons,” World Psychiatry: Official Journal of the World Psychiatric Association (WPA) 12, no. 2 (2013): 137–148. 23737423 10.1002/wps.20038 PMC3683266

[5] F. D. Broccard , T. Mullen , Y. M. Chi , et al., “Closed‐Loop Brain‐Machine‐Body Interfaces for Noninvasive Rehabilitation of Movement Disorders,” Annals of Biomedical Engineering 42, no. 8 (2014): 1573–1593. 24833254 10.1007/s10439-014-1032-6 PMC4099421

[6] M. Huhn , M. Tardy , L. M. Spineli , et al., “Efficacy of Pharmacotherapy and Psychotherapy for Adult Psychiatric Disorders: A Systematic Overview of Meta‐Analyses,” JAMA Psychiatry 71, no. 6 (2014): 706–715. 24789675 10.1001/jamapsychiatry.2014.112

[7] I. H. Lin , H. T. Tsai , C. Y. Wang , C. Y. Hsu , T. H. Liou , and Y. N. Lin , “Effectiveness and Superiority of Rehabilitative Treatments in Enhancing Motor Recovery Within 6 Months Poststroke: A Systemic Review,” Archives of Physical Medicine and Rehabilitation 100, no. 2 (2019): 366–378. 30686327 10.1016/j.apmr.2018.09.123

[8] R. van der Vliet , R. W. Selles , E. R. Andrinopoulou , et al., “Predicting Upper Limb Motor Impairment Recovery After Stroke: A Mixture Model,” Annals of Neurology 87, no. 3 (2020): 383–393. 31925838 10.1002/ana.25679 PMC7065018

[9] A. Antonini , P. Odin , R. Pahwa , et al., “The Long‐Term Impact of Levodopa/Carbidopa Intestinal Gel on 'Off'‐Time in Patients With Advanced Parkinson's Disease: A Systematic Review,” Advances in Therapy 38, no. 6 (2021): 2854–2890. 34018146 10.1007/s12325-021-01747-1 PMC8189983

[10] A. Cano , E. Fonseca , M. Ettcheto , et al., “Epilepsy in Neurodegenerative Diseases: Related Drugs and Molecular Pathways,” Pharmacy 14, no. 10 (2021): 1057. 10.3390/ph14101057 PMC8538968 34681281

[11] K. S. Kverno and E. Mangano , “Treatment‐Resistant Depression: Approaches to Treatment,” Journal of Psychosocial Nursing and Mental Health Services 59, no. 9 (2021): 7–11. 10.3928/02793695-20210816-01 34459676

[12] R. Cohen Kadosh , “Rethinking Excitation/Inhibition Balance in the Human Brain,” Nature Reviews Neuroscience 26, no. 8 (2025): 451–452. 10.1038/s41583-025-00943-0 40551004

[13] H. E. Marei , “Neural Circuit Mapping and Neurotherapy‐Based Strategies,” Cellular and Molecular Neurobiology 45, no. 1 (2025): 75. 40715588 10.1007/s10571-025-01595-5 PMC12297084

[14] M. C. Dadarlat , R. A. Canfield , and A. L. Orsborn , “Neural Plasticity in Sensorimotor Brain‐Machine Interfaces,” Annual Review of Biomedical Engineering 25 (2023): 51–76. 10.1146/annurev-bioeng-110220-110833 PMC10791144 36854262

[15] R. Mane , T. Chouhan , and C. Guan , “BCI for Stroke Rehabilitation: Motor and Beyond,” Journal of Neural Engineering 17, no. 4 (2020): 041001. 32613947 10.1088/1741-2552/aba162

[16] D. Chen , Z. Zhao , S. Zhang , et al., “Evolving Therapeutic Landscape of Intracerebral Hemorrhage: Emerging Cutting‐Edge Advancements in Surgical Robots, Regenerative Medicine, and Neurorehabilitation Techniques,” Translational Stroke Research 16, no. 3 (2024): 975–989. 38558011 10.1007/s12975-024-01244-x PMC12045821

[17] Y. N. Ma , K. Karako , P. Song , X. Hu , and Y. Xia , “Integrative Neurorehabilitation Using Brain‐Computer Interface: From Motor Function to Mental Health After Stroke,” BioScience Trends 19, no. 3 (2025): 243–251. 40240152 10.5582/bst.2025.01109

[18] J. Li , W. Zhang , Y. Liao , et al., “Neural Decoding Reliability: Breakthroughs and Potential of Brain‐Computer Interfaces Technologies in the Treatment of Neurological Diseases,” Physics of Life Reviews 55 (2025): 1–40. 40850267 10.1016/j.plrev.2025.08.007

[19] K. Kobayashi , K. N. Taylor , H. Shahabi , et al., “Effective Connectivity Relates Seizure Outcome to Electrode Placement in Responsive Neurostimulation,” Brain Communications 6, no. 1 (2024): fcae035. 38390255 10.1093/braincomms/fcae035 PMC10882982

[20] B. J. Edelman , S. Zhang , G. Schalk , et al., “Non‐Invasive Brain‐Computer Interfaces: State of the Art and Trends,” IEEE Reviews in Biomedical Engineering 18 (2025): 26–49. 39186407 10.1109/RBME.2024.3449790 PMC11861396

[21] H. Zheng , L. Niu , W. Qiu , et al., “The Emergence of Functional Ultrasound for Noninvasive Brain–Computer Interface,” Research 6 (2023): 0200. 37588619 10.34133/research.0200 PMC10427153

[22] M. F. Mridha , S. C. Das , M. M. Kabir , A. A. Lima , M. R. Islam , and Y. Watanobe , “Brain‐Computer Interface: Advancement and Challenges,” Sensors 21, no. 17 (2021): 5746. 34502636 10.3390/s21175746 PMC8433803

[23] Y. Sun , X. Chen , B. Liu , et al., “Signal Acquisition of Brain–Computer Interfaces: A Medical‐Engineering Crossover Perspective Review,” Fundamental Research 5, no. 1 (2024): 3–16. 40166113 10.1016/j.fmre.2024.04.011 PMC11955058

[24] M. W. Slutzky , “Brain‐Machine Interfaces: Powerful Tools for Clinical Treatment and Neuroscientific Investigations,” Neuroscientist 25, no. 2 (2019): 139–154. 29772957 10.1177/1073858418775355 PMC6611552

[25] B. F. Saway , C. Palmer , C. Hughes , et al., “The Evolution of Neuromodulation for Chronic Stroke: From Neuroplasticity Mechanisms to Brain‐Computer Interfaces,” Neurotherapeutics: The Journal of the American Society for Experimental NeuroTherapeutics 21, no. 3 (2024): e00337. 38377638 10.1016/j.neurot.2024.e00337 PMC11103214

[26] S. Yang , R. Li , H. Li , et al., “Exploring the Use of Brain‐Computer Interfaces in Stroke Neurorehabilitation,” BioMed Research International 2021 (2021): 9967348. 34239936 10.1155/2021/9967348 PMC8235968

[27] D. Chen , Z. Zhao , J. Shi , et al., “Harnessing the Sensing and Stimulation Function of Deep Brain‐Machine Interfaces: A New Dawn for Overcoming Substance Use Disorders,” Translational Psychiatry 14, no. 1 (2024): 1–11. 39419976 10.1038/s41398-024-03156-8 PMC11487193

[28] X. Cai , Z. Xu , J. Liu , R. Wang , and Y. Wu , “Recent Advances in Intracortical Neural Interfaces for Freely Moving Animals: Technologies and Applications,” Engineering 44 (2025): 73–86.

[29] J. F. M. Brannigan , A. Fry , N. L. Opie , B. C. V. Campbell , P. J. Mitchell , and T. J. Oxley , “Endovascular Brain‐Computer Interfaces in Poststroke Paralysis,” Stroke; A Journal of Cerebral Circulation 55, no. 2 (2024): 474–483. 10.1161/STROKEAHA.123.037719 38018832

[30] M. J. Vansteensel , E. G. M. Pels , M. G. Bleichner , et al., “Fully Implanted Brain–Computer Interface in a Locked‐in Patient With ALS,” New England Journal of Medicine 375, no. 21 (2016): 2060–2066. 27959736 10.1056/NEJMoa1608085 PMC5326682

[31] C. S. Mestais , G. Charvet , F. Sauter‐Starace , M. Foerster , D. Ratel , and A. L. Benabid , “WIMAGINE: Wireless 64‐Channel ECoG Recording Implant for Long Term Clinical Applications,” IEEE Transactions on Neural Systems and Rehabilitation Engineering 23, no. 1 (2015): 10–21. 25014960 10.1109/TNSRE.2014.2333541

[32] V. S. Polikov , P. A. Tresco , and W. M. Reichert , “Response of Brain Tissue to Chronically Implanted Neural Electrodes,” Journal of Neuroscience Methods 148, no. 1 (2005): 1–18. 16198003 10.1016/j.jneumeth.2005.08.015

[33] T. Saxena , L. Karumbaiah , E. A. Gaupp , et al., “The Impact of Chronic Blood‐Brain Barrier Breach on Intracortical Electrode Function,” Biomaterials 34, no. 20 (2013): 4703–4713. 23562053 10.1016/j.biomaterials.2013.03.007

[34] D. Liu , Y. Shan , P. Wei , et al., “Reclaiming Hand Functions After Complete Spinal Cord Injury With Epidural Brain‐Computer Interface,” Rehabilitation Medicine and Physical Therapy (2024), preprint, September 6, 2024, 10.1101/2024.09.05.24313041.

[35] P. Mitchell , S. C. M. Lee , P. E. Yoo , et al., “Assessment of Safety of a Fully Implanted Endovascular Brain‐Computer Interface for Severe Paralysis in 4 Patients: The Stentrode With Thought‐Controlled Digital Switch (SWITCH) Study,” JAMA Neurology 80, no. 3 (2023): 270. 36622685 10.1001/jamaneurol.2022.4847 PMC9857731

[36] M. Taylor‐Rowan , G. Cuthbertson , R. Keir , et al., “The Prevalence of Frailty Among Acute Stroke Patients, and Evaluation of Method of Assessment,” Clinical Rehabilitation 33, no. 10 (2019): 1688–1696. 30971115 10.1177/0269215519841417

[37] R. A. Sastry , N. J. Pertsch , O. Tang , B. Shao , S. A. Toms , and R. J. Weil , “Frailty and Outcomes After Craniotomy for Brain Tumor,” Journal of Clinical Neuroscience 81 (2020): 95–100. 33222979 10.1016/j.jocn.2020.09.002

[38] W. J. Powers , A. A. Rabinstein , T. Ackerson , et al., “Guidelines for the Early Management of Patients With Acute Ischemic Stroke: 2019 Update to the 2018 Guidelines for the Early Management of Acute Ischemic Stroke: A Guideline for Healthcare Professionals From the American Heart Association/American Stroke Association,” Stroke; A Journal of Cerebral Circulation 50, no. 12 (2019): e344–e418. 10.1161/STR.0000000000000211 31662037

[39] S. M. Greenberg , W. C. Ziai , C. Cordonnier , et al., “2022 Guideline for the Management of Patients With Spontaneous Intracerebral Hemorrhage: A Guideline From the American Heart Association/American Stroke Association,” Stroke; A Journal of Cerebral Circulation 53, no. 7 (2022): e282–e361. 10.1161/STR.0000000000000407 35579034

[40] M. Goyal , B. K. Menon , W. H. van Zwam , et al., “Endovascular Thrombectomy After Large‐Vessel Ischaemic Stroke: A Meta‐Analysis of Individual Patient Data From Five Randomised Trials,” Lancet 387, no. 10029 (2016): 1723–1731. 26898852 10.1016/S0140-6736(16)00163-X

[41] T. J. Oxley , N. L. Opie , S. E. John , et al., “Minimally Invasive Endovascular Stent‐Electrode Array for High‐Fidelity, Chronic Recordings of Cortical Neural Activity,” Nature Biotechnology 34, no. 3 (2016): 320–327. 10.1038/nbt.3428 26854476

[42] Q. He , Y. Yang , P. Ge , et al., “The Brain Nebula: Minimally Invasive Brain‐Computer Interface by Endovascular Neural Recording and Stimulation,” Journal of Neurointerventional Surgery 16, no. 12 (2024): 1237–1243. 38388478 10.1136/jnis-2023-021296 PMC11671944

[43] J. Veldema and A. Gharabaghi , “Non‐Invasive Brain Stimulation for Improving Gait, Balance, and Lower Limbs Motor Function in Stroke,” Journal of NeuroEngineering and Rehabilitation 19, no. 1 (2022): 84. 35922846 10.1186/s12984-022-01062-y PMC9351139

[44] I. Rektorová , M. Pupíková , L. Fleury , L. Brabenec , and F. C. Hummel , “Non‐invasive Brain Stimulation: Current and Future Applications in Neurology,” Nature Reviews Neurology 21, no. 12 (2025): 669–686. 40957931 10.1038/s41582-025-01137-z

[45] J. Dawson , A. H. Abdul‐Rahim , and T. J. Kimberley , “Neurostimulation for Treatment of Post‐Stroke Impairments,” Nature Reviews Neurology 20, no. 5 (2024): 259–268. 38570705 10.1038/s41582-024-00953-z

[46] FDA , "FDA Approves First‐of‐Its‐Kind Stroke Rehabilitation System," FDA, August 31, 2021, https://www.fda.gov/news‐events/press‐announcements/fda‐approves‐first‐its‐kind‐stroke‐rehabilitation‐system.

[47] A. M. Lozano , N. Lipsman , H. Bergman , et al., “Deep Brain Stimulation: Current Challenges and Future Directions,” Nature Reviews Neurology 15, no. 3 (2019): 148–160. 30683913 10.1038/s41582-018-0128-2 PMC6397644

[48] F. V. Gouveia , N. M. Warsi , H. Suresh , R. Matin , and G. M. Ibrahim , “Neurostimulation Treatments for Epilepsy: Deep Brain Stimulation, Responsive Neurostimulation and Vagus Nerve Stimulation,” Neurotherapeutics: The Journal of the American Society for Experimental NeuroTherapeutics 21, no. 3 (2024): e00308. 38177025 10.1016/j.neurot.2023.e00308 PMC11103217

[49] N. Grossman , D. Bono , N. Dedic , et al., “Noninvasive Deep Brain Stimulation via Temporally Interfering Electric Fields,” Cell 169, no. 6 (2017): 1029–1041.e16. 28575667 10.1016/j.cell.2017.05.024 PMC5520675

[50] R. Beisteiner , M. Hallett , and A. M. Lozano , “Ultrasound Neuromodulation as a New Brain Therapy,” Advanced Science 10, no. 14 (2023): e2205634. 36961104 10.1002/advs.202205634 PMC10190662

[51] H. Lorach , A. Galvez , V. Spagnolo , et al., “Walking Naturally After Spinal Cord Injury Using a Brain‐Spine Interface,” Nature 618, no. 7963 (2023): 126–133. 37225984 10.1038/s41586-023-06094-5 PMC10232367

[52] S. He , F. Baig , A. Merla , et al., “Beta‐Triggered Adaptive Deep Brain Stimulation During Reaching Movement in Parkinson's Disease,” Brain: A Journal of Neurology 146, no. 12 (2023): 5015–5030. 37433037 10.1093/brain/awad233 PMC10690014

[53] N. D. Sisterson , V. Kokkinos , A. Urban , N. Li , and R. M. Richardson , “Responsive Neurostimulation of the Thalamus Improves Seizure Control in Idiopathic Generalised Epilepsy: Initial Case Series,” Journal of Neurology, Neurosurgery, and Psychiatry 93, no. 5 (2022): 491–498. 35217517 10.1136/jnnp-2021-327512 PMC9016239

[54] GBD 2019 Stroke Collaborators . Global, Regional, and National Burden of Stroke and Its Risk Factors, 1990–2019: A Systematic Analysis for the Global Burden of Disease Study 2019. Lancet Neurology 20, no. 10 (2021): 795–820. 34487721 10.1016/S1474-4422(21)00252-0 PMC8443449

[55] O. Beylerli , I. Gareev , H. Shi , and T. Ilyasova , “Effect of Mesenchymal Stem Cell‐Derived Exosomes on the Inflammatory Response After Stroke,” Brain Hemorrhages 5, no. 5 (2024): 248–256.

[56] C. W. Tsao , A. W. Aday , Z. I. Almarzooq , et al., “Heart Disease and Stroke Statistics‐2022 Update: A Report From the American Heart Association,” Circulation 145, no. 8 (2022): e153–e639. 35078371 10.1161/CIR.0000000000001052

[57] E. S. Lawrence , C. Coshall , R. Dundas , et al., “Estimates of the Prevalence of Acute Stroke Impairments and Disability in a Multiethnic Population,” Stroke; A Journal of Cerebral Circulation 32, no. 6 (2001): 1279–1284. 10.1161/01.str.32.6.1279 11387487

[58] D. T. Wade and R. L. Hewer , “Motor Loss and Swallowing Difficulty After Stroke: Frequency, Recovery, and Prognosis,” Acta Neurologica Scandinavica 76, no. 1 (1987): 50–54. 3630644 10.1111/j.1600-0404.1987.tb03543.x

[59] U. Hammerbeck , S. F. Tyson , P. Samraj , K. Hollands , J. W. Krakauer , and J. Rothwell , “The Strength of the Corticospinal Tract Not the Reticulospinal Tract Determines Upper‐Limb Impairment Level and Capacity for Skill‐Acquisition in the Sub‐Acute Post‐Stroke Period,” Neurorehabilitation and Neural Repair 35, no. 9 (2021): 812–822. 34219510 10.1177/15459683211028243 PMC8414832

[60] C. J. Winstein , J. Stein , R. Arena , et al., “Guidelines for Adult Stroke Rehabilitation and Recovery: A Guideline for Healthcare Professionals From the American Heart Association/American Stroke Association,” Stroke; A Journal of Cerebral Circulation 47, no. 6 (2016): e98–e169. 10.1161/STR.0000000000000098 27145936

[61] E. R. Coleman , R. Moudgal , K. Lang , et al., “Early Rehabilitation After Stroke: A Narrative Review,” Current Atherosclerosis Reports 19, no. 12 (2017): 59. 29116473 10.1007/s11883-017-0686-6 PMC5802378

[62] M. Gittler and A. M. Davis , “Guidelines for Adult Stroke Rehabilitation and Recovery,” JAMA 319, no. 8 (2018): 820–821. 29486016 10.1001/jama.2017.22036

[63] C. M. Stinear , C. E. Lang , S. Zeiler , and W. D. Byblow , “Advances and Challenges in Stroke Rehabilitation,” Lancet Neurology 19, no. 4 (2020): 348–360. 32004440 10.1016/S1474-4422(19)30415-6

[64] U. Chaudhary , N. Birbaumer , and A. Ramos‐Murguialday , “Brain–Computer Interfaces for Communication and Rehabilitation,” Nature Reviews Neurology 12, no. 9 (2016): 513–525. 27539560 10.1038/nrneurol.2016.113

[65] R. Mane , Z. Wu , and D. Wang , “Poststroke Motor, Cognitive and Speech Rehabilitation With Brain‐Computer Interface: A Perspective Review,” Stroke and Vascular Neurology 7, no. 6 (2022): 541–549. 35853669 10.1136/svn-2022-001506 PMC9811566

[66] S. N. Flesher , J. E. Downey , J. M. Weiss , et al., “A Brain‐Computer Interface That Evokes Tactile Sensations Improves Robotic Arm Control,” Science 372, no. 6544 (2021): 831–836. 34016775 10.1126/science.abd0380 PMC8715714

[67] C. Marquez‐Chin and M. R. Popovic , “Functional Electrical Stimulation Therapy for Restoration of Motor Function After Spinal Cord Injury and Stroke: A Review,” Biomedical Engineering Online 19 (2020): 34. 32448143 10.1186/s12938-020-00773-4 PMC7245767

[68] A. Biasiucci , R. Leeb , I. Iturrate , et al., “Brain‐Actuated Functional Electrical Stimulation Elicits Lasting Arm Motor Recovery After Stroke,” Nature Communications 9, no. 1 (2018): 2421. 10.1038/s41467-018-04673-z PMC6010454 29925890

[69] K. B. Baker , E. B. Plow , S. Nagel , et al., “Cerebellar Deep Brain Stimulation for Chronic Post‐Stroke Motor Rehabilitation: A Phase I Trial,” Nature Medicine 29, no. 9 (2023): 2366–2374. 10.1038/s41591-023-02507-0 PMC10504081 37580534

[70] M. P. Powell , N. Verma , E. Sorensen , et al., “Epidural Stimulation of the Cervical Spinal Cord for Post‐Stroke Upper‐Limb Paresis,” Nature Medicine 29, no. 3 (2023): 689–699. 10.1038/s41591-022-02202-6 PMC10291889 36807682

[71] R. D. Penn , S. K. Hilal , W. J. Michelsen , E. S. Goldensohn , and J. Driller , “Intravascular Intracranial EEG Recording. Technical Note,” Journal of Neurosurgery 38, no. 2 (1973): 239–243. 4632831 10.3171/jns.1973.38.2.0239

[72] J. Z. Fan , V. Lopez‐Rivera , and S. A. Sheth , “Over the Horizon: The Present and Future of Endovascular Neural Recording and Stimulation,” Frontiers in Neuroscience 14 (2020): 432. 32435184 10.3389/fnins.2020.00432 PMC7218134

[73] L. Leishangthem , P. SirDeshpande , D. Dua , and S. R. Satti , “Dural Venous Sinus Stenting for Idiopathic Intracranial Hypertension: An Updated Review,” Journal of Neuroradiology [Journal De Neuroradiologie] 46, no. 2 (2019): 148–154. 30219337 10.1016/j.neurad.2018.09.001

[74] T. J. Oxley , P. E. Yoo , G. S. Rind , et al., “Motor Neuroprosthesis Implanted With Neurointerventional Surgery Improves Capacity for Activities of Daily Living Tasks in Severe Paralysis: First In‐Human Experience,” Journal of NeuroInterventional Surgery 13, no. 2 (2021): 102–108. 33115813 10.1136/neurintsurg-2020-016862 PMC7848062

[75] N. L. Opie , N. R. van der Nagel , S. E. John , et al., “Micro‐CT and Histological Evaluation of an Neural Interface Implanted Within a Blood Vessel,” IEEE Transactions on Bio‐Medical Engineering 64, no. 4 (2017): 928–934. 27337706 10.1109/TBME.2016.2552226

[76] N. L. Opie , S. E. John , G. S. Rind , et al., “Chronic Impedance Spectroscopy of an Endovascular Stent‐Electrode Array,” Journal of Neural Engineering 13, no. 4 (2016): 046020. 27378157 10.1088/1741-2560/13/4/046020

[77] S. E. John , N. L. Opie , Y. T. Wong , et al., “Signal Quality of Simultaneously Recorded Endovascular, Subdural and Epidural Signals Are Comparable,” Scientific Reports 8, no. 1 (2018): 8427. 29849104 10.1038/s41598-018-26457-7 PMC5976775

[78] B. Thielen , H. Xu , T. Fujii , et al., “Making a Case for Endovascular Approaches for Neural Recording and Stimulation,” Journal of Neural Engineering 20, no. 1 (2023): 011001. 10.1088/1741-2552/acb086 PMC9928900 36603221

[79] H. C. H. Lui , Y. T. Ng , S. C. H. Yu , J. T. F. Zhuang , and G. K. C. Wong , “Mid‐Term Outcomes in Stent‐Assisted Coil Embolization for Ruptured Cerebral Aneurysms in the Acute Period: A Single Institution Retrospective Review,” Brain Hemorrhages 5, no. 6 (2024): 267–273.

[80] S. A. Raza , N. L. Opie , A. Morokoff , R. P. Sharma , P. J. Mitchell , and T. J. Oxley , “Endovascular Neuromodulation: Safety Profile and Future Directions,” Frontiers in Neurology 11 (2020): 351. 32390937 10.3389/fneur.2020.00351 PMC7193719

[81] B. Abu‐El‐Haija , P. D. Bhave , D. N. Campbell , et al., “Venous Stenosis After Transvenous Lead Placement: A Study of Outcomes and Risk Factors in 212 Consecutive Patients,” Journal of the American Heart Association 4, no. 8 (2015): e001878. 26231843 10.1161/JAHA.115.001878 PMC4599456

[82] C. Sticherling , S. P. Chough , R. L. Baker , et al., “Prevalence of Central Venous Occlusion in Patients With Chronic Defibrillator Leads,” American Heart Journal 141, no. 5 (2001): 813–816. 11320371 10.1067/mhj.2001.114195

[83] D. A. Moses , S. L. Metzger , J. R. Liu , et al., “Neuroprosthesis for Decoding Speech in a Paralyzed Person With Anarthria,” New England Journal of Medicine 385, no. 3 (2021): 217–227. 34260835 10.1056/NEJMoa2027540 PMC8972947

[84] B. Jarosiewicz , A. A. Sarma , D. Bacher , et al., “Virtual Typing by People With Tetraplegia Using a Self‐Calibrating Intracortical Brain‐Computer Interface,” Science Translational Medicine 7, no. 313 (2015): 313ra179. 10.1126/scitranslmed.aac7328 PMC4765319 26560357

[85] D. Navarro‐Orozco and J. C. Sánchez‐Manso , “Neuroanatomy, Middle Cerebral artery,” in StatPearls [Internet] (StatPearls Publishing, 2023), https://www.ncbi.nlm.nih.gov/books/NBK526002/. 30252258

[86] Y. S. Ng , J. Stein , M. Ning , and R. M. Black‐Schaffer , “Comparison of Clinical Characteristics and Functional Outcomes of Ischemic Stroke in Different Vascular Territories,” Stroke; A Journal of Cerebral Circulation 38, no. 8 (2007): 2309–2314. 10.1161/STROKEAHA.106.475483 17615368

[87] K. N. Sheth , “Spontaneous Intracerebral Hemorrhage,” New England Journal of Medicine 387, no. 17 (2022): 1589–1596. 36300975 10.1056/NEJMra2201449

[88] T. Yanagisawa , M. Hirata , Y. Saitoh , et al., “Neural Decoding Using Gyral and Intrasulcal Electrocorticograms,” Neuroimage 45, no. 4 (2009): 1099–1106. 19349227 10.1016/j.neuroimage.2008.12.069

[89] A. Nambu , “Somatotopic Organization of the Primate Basal Ganglia,” Frontiers in Neuroanatomy 5 (2011): 26. 21541304 10.3389/fnana.2011.00026 PMC3082737

[90] P. Romanelli , V. Esposito , D. W. Schaal , and G. Heit , “Somatotopy in the Basal Ganglia: Experimental and Clinical Evidence for Segregated Sensorimotor Channels,” Brain Research Reviews 48, no. 1 (2005): 112–128. 15708631 10.1016/j.brainresrev.2004.09.008

[91] B. A. Teplitzky , A. T. Connolly , J. A. Bajwa , and M. D. Johnson , “Computational Modeling of an Endovascular Approach to Deep Brain Stimulation,” Journal of Neural Engineering 11, no. 2 (2014): 026011. 24608363 10.1088/1741-2560/11/2/026011

[92] J. L. Collinger , B. Wodlinger , J. E. Downey , et al., “High‐Performance Neuroprosthetic Control by an Individual With Tetraplegia,” Lancet 381, no. 9866 (2013): 557–564. 23253623 10.1016/S0140-6736(12)61816-9 PMC3641862

[93] Y. Zhang , Y. Gao , J. Zhou , Z. Zhang , M. Feng , and Y. Liu , “Advances in Brain‐Computer Interface Controlled Functional Electrical Stimulation for Upper Limb Recovery After Stroke,” Brain Research Bulletin 226 (2025): 111354. 40280369 10.1016/j.brainresbull.2025.111354

[94] B. Wodlinger , J. E. Downey , E. C. Tyler‐Kabara , A. B. Schwartz , M. L. Boninger , and J. L. Collinger , “Ten‐Dimensional Anthropomorphic Arm Control in a Human Brain‐Machine Interface: Difficulties, Solutions, and Limitations,” Journal of Neural Engineering 12, no. 1 (2015): 016011. 25514320 10.1088/1741-2560/12/1/016011

[95] A. B. Ajiboye , F. R. Willett , D. R. Young , et al., “Restoration of Reaching and Grasping Movements Through Brain‐Controlled Muscle Stimulation in a Person With Tetraplegia: A Proof‐of‐Concept Demonstration,” Lancet 389, no. 10081 (2017): 1821–1830. 28363483 10.1016/S0140-6736(17)30601-3 PMC5516547

[96] A. Zhang , E. T. Mandeville , L. Xu , C. M. Stary , E. H. Lo , and C. M. Lieber , “Ultraflexible Endovascular Probes for Brain Recording Through Micrometer‐Scale Vasculature,” Science 381, no. 6655 (2023): 306–312. 37471542 10.1126/science.adh3916 PMC11412271

[97] M. M. Shanechi , R. C. Hu , M. Powers , G. W. Wornell , E. N. Brown , and Z. M. Williams , “Neural Population Partitioning and a Concurrent Brain‐Machine Interface for Sequential Motor Function,” Nature Neuroscience 15, no. 12 (2012): 1715–1722. 23143511 10.1038/nn.3250 PMC3509235

[98] M. P. Branco , Z. V. Freudenburg , E. J. Aarnoutse , M. G. Bleichner , M. J. Vansteensel , and N. F. Ramsey , “Decoding Hand Gestures From Primary Somatosensory Cortex Using High‐Density ECoG,” Neuroimage 147 (2017): 130–142. 27926827 10.1016/j.neuroimage.2016.12.004 PMC5322832

[99] J. R. Allen , S. R. Karri , C. Yang , and M. E. Stoykov , “Spinal Cord Stimulation for Poststroke Hemiparesis: A Scoping Review,” American Journal of Occupational Therapy 78, no. 2 (2024): 7802180220. 10.5014/ajot.2024.050533 PMC11017736 38477681

[100] A. Rowald , S. Komi , R. Demesmaeker , et al., “Activity‐Dependent Spinal Cord Neuromodulation Rapidly Restores Trunk and Leg Motor Functions After Complete Paralysis,” Nature Medicine 28, no. 2 (2022): 260–271. 10.1038/s41591-021-01663-5 35132264

[101] R. Melzack and P. D. Wall , “Pain Mechanisms: A New Theory,” Science 150, no. 3699 (1965): 971–979. 5320816 10.1126/science.150.3699.971

[102] R. Ali and J. M. Schwalb , “History and Future of Spinal Cord Stimulation,” Neurosurgery 94, no. 1 (2024): 20–28. 37681953 10.1227/neu.0000000000002654

[103] M. R. Dimitrijevic , Y. Gerasimenko , and M. M. Pinter , “Evidence for a Spinal Central Pattern Generator in Humans,” Annals of the New York Academy of Sciences 860 (1998): 360–376. 9928325 10.1111/j.1749-6632.1998.tb09062.x

[104] S. Grillner and P. Zangger , “On the Central Generation of Locomotion in the Low Spinal Cat,” Experimental Brain Research 34, no. 2 (1979): 241–261. 421750 10.1007/BF00235671

[105] Y. Gerasimenko , R. R. Roy , and V. R. Edgerton , “Epidural Stimulation: Comparison of the Spinal Circuits That Generate and Control Locomotion in Rats, Cats and Humans,” Experimental Neurology 209, no. 2 (2008): 417–425. 17850791 10.1016/j.expneurol.2007.07.015 PMC2288525

[106] R. D. De Leon , J. A. Hodgson , R. R. Roy , and V. R. Edgerton , “Full Weight‐Bearing Hindlimb Standing Following Stand Training in the Adult Spinal Cat,” Journal of Neurophysiology 80, no. 1 (1998): 83–91. 9658030 10.1152/jn.1998.80.1.83

[107] G. Courtine , Y. Gerasimenko , R. van den Brand , et al., “Transformation of Nonfunctional Spinal Circuits Into Functional States After the Loss of Brain Input,” Nature Neuroscience 12, no. 10 (2009): 1333–1342. 19767747 10.1038/nn.2401 PMC2828944

[108] R. Herman , J. He , S. D'Luzansky , W. Willis , and S. Dilli , “Spinal Cord Stimulation Facilitates Functional Walking in a Chronic, Incomplete Spinal Cord Injured,” Spinal Cord 40, no. 2 (2002): 65–68. 11926417 10.1038/sj.sc.3101263

[109] B. Jilge , K. Minassian , F. Rattay , et al., “Initiating Extension of the Lower Limbs in Subjects With Complete Spinal Cord Injury by Epidural Lumbar Cord Stimulation,” Experimental Brain Research 154, no. 3 (2004): 308–326. 14586532 10.1007/s00221-003-1666-3

[110] K. Minassian , B. Jilge , F. Rattay , et al., “Stepping‐Like Movements in Humans With Complete Spinal Cord Injury Induced by Epidural Stimulation of the Lumbar Cord: Electromyographic Study of Compound Muscle Action Potentials,” Spinal Cord 42, no. 7 (2004): 401–416. 15124000 10.1038/sj.sc.3101615

[111] B. Jilge , K. Minassian , F. Rattay , and M. R. Dimitrijevic , “Frequency‐Dependent Selection of Alternative Spinal Pathways With Common Periodic Sensory Input,” Biological Cybernetics 91, no. 6 (2004): 359–376. 15597176 10.1007/s00422-004-0511-5

[112] K. Minassian , I. Persy , F. Rattay , M. M. Pinter , H. Kern , and M. R. Dimitrijevic , “Human Lumbar Cord Circuitries Can be Activated by Extrinsic Tonic Input to Generate Locomotor‐Like Activity,” Human Movement Science 26, no. 2 (2007): 275–295. 17343947 10.1016/j.humov.2007.01.005

[113] V. R. Edgerton , G. Courtine , Y. P. Gerasimenko , et al., “Training Locomotor Networks,” Brain Research Reviews 57, no. 1 (2008): 241–254. 18022244 10.1016/j.brainresrev.2007.09.002 PMC2288528

[114] E. M. Moraud , M. Capogrosso , E. Formento , et al., “Mechanisms Underlying the Neuromodulation of Spinal Circuits for Correcting Gait and Balance Deficits After Spinal Cord Injury,” Neuron 89, no. 4 (2016): 814–828. 26853304 10.1016/j.neuron.2016.01.009

[115] S. Harkema , Y. Gerasimenko , J. Hodes , et al., “Effect of Epidural Stimulation of the Lumbosacral Spinal Cord on Voluntary Movement, Standing, and Assisted Stepping After Motor Complete Paraplegia: A Case Study,” Lancet 377, no. 9781 (2011): 1938–1947. 21601270 10.1016/S0140-6736(11)60547-3 PMC3154251

[116] C. A. Angeli , V. R. Edgerton , Y. P. Gerasimenko , and S. J. Harkema , “Altering Spinal Cord Excitability Enables Voluntary Movements After Chronic Complete Paralysis in Humans,” Brain 137, no. pt. 5 (2014): 1394–1409. 24713270 10.1093/brain/awu038 PMC3999714

[117] E. Rejc , C. Angeli , and S. Harkema , “Effects of Lumbosacral Spinal Cord Epidural Stimulation for Standing After Chronic Complete Paralysis in Humans,” PLoS ONE 10, no. 7 (2015): e0133998. 26207623 10.1371/journal.pone.0133998 PMC4514797

[118] M. L. Gill , P. J. Grahn , J. S. Calvert , et al., “Neuromodulation of Lumbosacral Spinal Networks Enables Independent Stepping After Complete Paraplegia,” Nature Medicine 24, no. 11 (2018): 1677–1682. 10.1038/s41591-018-0175-7 30250140

[119] C. A. Angeli , M. Boakye , R. A. Morton , et al., “Recovery of Over‐Ground Walking After Chronic Motor Complete Spinal Cord Injury,” New England Journal of Medicine 379, no. 13 (2018): 1244–1250. 30247091 10.1056/NEJMoa1803588

[120] E. Rejc , C. A. Angeli , D. Atkinson , and S. J. Harkema , “Motor Recovery After Activity‐Based Training With Spinal Cord Epidural Stimulation in a Chronic Motor Complete Paraplegic,” Scientific Reports 7, no. 1 (2017): 13476. 29074997 10.1038/s41598-017-14003-w PMC5658385

[121] T. Guiho , S. N. Baker , and A. Jackson , “Epidural and Transcutaneous Spinal Cord Stimulation Facilitates Descending Inputs to Upper‐Limb Motoneurons in Monkeys,” Journal of Neural Engineering 18, no. 4 (2021): 046011. 10.1088/1741-2552/abe358 PMC8208633 33540399

[122] M. Capogrosso , J. M. Balaguer , G. Prat‐Ortega , et al., “Supraspinal Control of Motoneurons After Paralysis Enabled by Spinal Cord Stimulation,” Research Square (2025): rs.3.rs–3650257, 10.21203/rs.3.rs-3650257/v1.

[123] Y. Nishimura , S. I. Perlmutter , R. W. Eaton , and E. E. Fetz , “Spike‐Timing‐Dependent Plasticity in Primate Corticospinal Connections Induced During Free Behavior,” Neuron 80, no. 5 (2013): 1301–1309. 24210907 10.1016/j.neuron.2013.08.028 PMC4079851

[124] E. Formento , K. Minassian , F. Wagner , et al., “Electrical Spinal Cord Stimulation Must Preserve Proprioception to Enable Locomotion in Humans With Spinal Cord Injury,” Nature Neuroscience 21, no. 12 (2018): 1728–1741. 30382196 10.1038/s41593-018-0262-6 PMC6268129

[125] N. Wenger , E. M. Moraud , S. Raspopovic , et al., “Closed‐Loop Neuromodulation of Spinal Sensorimotor Circuits Controls Refined Locomotion After Complete Spinal Cord Injury,” Science Translational Medicine 6, no. 255 (2014): 255ra133. 10.1126/scitranslmed.3008325 25253676

[126] N. Wenger , E. M. Moraud , J. Gandar , et al., “Spatiotemporal Neuromodulation Therapies Engaging Muscle Synergies Improve Motor Control After Spinal Cord Injury,” Nature Medicine 22, no. 2 (2016): 138–145. 10.1038/nm.4025 PMC5061079 26779815

[127] F. B. Wagner , J. B. Mignardot , C. G. Le Goff‐Mignardot , et al., “Targeted Neurotechnology Restores Walking in Humans With Spinal Cord Injury,” Nature 563, no. 7729 (2018): 65–71. 30382197 10.1038/s41586-018-0649-2

[128] M. Capogrosso , T. Milekovic , D. Borton , et al., “A Brain‐spine Interface Alleviating Gait Deficits After Spinal Cord Injury in Primates,” Nature 539, no. 7628 (2016): 284–288. 27830790 10.1038/nature20118 PMC5108412

[129] M. Bonizzato , G. Pidpruzhnykova , J. DiGiovanna , et al., “Brain‐Controlled Modulation of Spinal Circuits Improves Recovery From Spinal Cord Injury,” Nature Communications 9, no. 1 (2018): 3015. 10.1038/s41467-018-05282-6 PMC6070513 30068906

[130] S. Yakovenko , V. Mushahwar , V. VanderHorst , G. Holstege , and A. Prochazka , “Spatiotemporal Activation of Lumbosacral Motoneurons in the Locomotor Step Cycle,” Journal of Neurophysiology 87, no. 3 (2002): 1542–1553. 11877525 10.1152/jn.00479.2001

[131] G. Cappellini , Y. P. Ivanenko , N. Dominici , R. E. Poppele , and F. Lacquaniti , “Migration of Motor Pool Activity in the Spinal Cord Reflects Body Mechanics in Human Locomotion,” Journal of Neurophysiology 104, no. 6 (2010): 3064–3073. 20881204 10.1152/jn.00318.2010

[132] Y. P. Ivanenko , R. Grasso , M. Zago , et al., “Temporal Components of the Motor Patterns Expressed by the Human Spinal Cord Reflect Foot Kinematics,” Journal of Neurophysiology 90, no. 5 (2003): 3555–3565. 12853436 10.1152/jn.00223.2003

[133] M. Capogrosso , F. B. Wagner , J. Gandar , et al., “Configuration of Electrical Spinal Cord Stimulation Through Real‐Time Processing of Gait Kinematics,” Nature Protocols 13, no. 9 (2018): 2031–2061. 30190556 10.1038/s41596-018-0030-9

[134] H. Lorach , G. Charvet , J. Bloch , and G. Courtine , “Brain–Spine Interfaces to Reverse Paralysis,” National Science Review 9, no. 10 (2022): nwac009. 36196116 10.1093/nsr/nwac009 PMC9522392

[135] L. D. Hachem , G. Balbinot , and M. G. Fehlings , “A Digital Bridge to Reverse Paralysis,” Cell Research 33, no. 12 (2023): 892–893. 37460803 10.1038/s41422-023-00845-9 PMC10709562

[136] S. Moorjani , S. Walvekar , E. E. Fetz , and S. I. Perlmutter , “Movement‐Dependent Electrical Stimulation for Volitional Strengthening of Cortical Connections in Behaving Monkeys,” Proceedings National Academy of Science USA 119, no. 27 (2022): e2116321119. 10.1073/pnas.2116321119 PMC9271159 35759657

[137] D. J. Guggenmos , M. Azin , S. Barbay , et al., “Restoration of Function After Brain Damage Using a Neural Prosthesis,” Proceedings National Academy of Science USA 110, no. 52 (2013): 21177–21182. 10.1073/pnas.1316885110 PMC3876197 24324155

[138] E. Pirondini , E. Carranza , J. M. Balaguer , et al., “Poststroke Arm and Hand Paresis: Should We Target the Cervical Spinal Cord?,” Trends in Neuroscience (Tins) 45, no. 8 (2022): 568–578. 35659414 10.1016/j.tins.2022.05.002

[139] K. S. Hayward and J. Bernhardt , “Aspiring to Restore Arm and Hand Function After Stroke,” Lancet Neurology 22, no. 6 (2023): 464–465. 37210089 10.1016/S1474-4422(23)00150-3

[140] D. M. Griffin and P. L. Strick , “The Motor Cortex Uses Active Suppression to Sculpt Movement,” Science Advances 6, no. 34 (2020): eabb8395. 32937371 10.1126/sciadv.abb8395 PMC7442473

[141] M. Kinoshita , R. Matsui , S. Kato , et al., “Genetic Dissection of the Circuit for Hand Dexterity in Primates,” Nature 487, no. 7406 (2012): 235–238. 22722837 10.1038/nature11206

[142] D. C. Lu , V. R. Edgerton , M. Modaber , et al., “Engaging Cervical Spinal Cord Networks to Reenable Volitional Control of Hand Function in Tetraplegic Patients,” Neurorehabilitation and Neural Repair 30, no. 10 (2016): 951–962. 27198185 10.1177/1545968316644344 PMC5374120

[143] B. Barra , S. Conti , M. G. Perich , et al., “Epidural Electrical Stimulation of the Cervical Dorsal Roots Restores Voluntary Upper Limb Control in Paralyzed Monkeys,” Nature Neuroscience 25, no. 7 (2022): 924–934. 35773543 10.1038/s41593-022-01106-5

[144] E. H. Choi , S. Gattas , N. J. Brown , et al., “Epidural Electrical Stimulation for Spinal Cord Injury,” Neural Regeneration Research 16, no. 12 (2021): 2367–2375. 33907008 10.4103/1673-5374.313017 PMC8374568

[145] C. Rosso , R. Valabregue , Y. Attal , et al., “Contribution of Corticospinal Tract and Functional Connectivity in Hand Motor Impairment After Stroke,” PLoS ONE 8, no. 9 (2013): e73164. 24086272 10.1371/journal.pone.0073164 PMC3785485

[146] T. Tian , S. Zhang , and M. Yang , “Recent Progress and Challenges in the Treatment of Spinal Cord Injury,” Protein Cell 14, no. 9 (2023): 635–652. 36856750 10.1093/procel/pwad003 PMC10501188

[147] Y. Moon , T. Zuleger , M. Lamberti , et al., “Characterization of Motor‐Evoked Responses Obtained With Transcutaneous Electrical Spinal Stimulation From the Lower‐Limb Muscles After Stroke,” Brain Sciences 11, no. 3 (2021): 289. 33652677 10.3390/brainsci11030289 PMC7996860

[148] X. Li , T. Xue , Z. Li , and J. Zhang , “Invasive Electrical Nerve Stimulation for Post‐Stroke Motor Rehabilitation,” Chinese Medical Journal 137, no. 20 (2024): 2495–2497. 39252152 10.1097/CM9.0000000000003286 PMC11479480

[149] R. M. de Freitas , A. Sasaki , D. G. Sayenko , et al., “Selectivity and Excitability of Upper‐Limb Muscle Activation During Cervical Transcutaneous Spinal Cord Stimulation in Humans,” Journal of Applied Physiology 131, no. 2 (2021): 746–759. 34138648 10.1152/japplphysiol.00132.2021

[150] Y. Giat , J. Mizrahi , and M. Levy , “A Musculotendon Model of the Fatigue Profiles of Paralyzed Quadriceps Muscle Under FES,” IEEE Transactions on Bio‐Medical Engineering 40, no. 7 (1993): 664–674. 8244427 10.1109/10.237696

[151] M. M. Shanechi , R. C. Hu , and Z. M. Williams , “A Cortical‐Spinal Prosthesis for Targeted Limb Movement in Paralysed Primate Avatars,” Nature Communications 5 (2014): 3237. 10.1038/ncomms4237 PMC3932632 24549394

[152] M. Capogrosso , N. Wenger , S. Raspopovic , et al., “A Computational Model for Epidural Electrical Stimulation of Spinal Sensorimotor Circuits,” Journal of Neuroscience: The Official Journal of the Society for Neuroscience 33, no. 49 (2013): 19326–19340. 24305828 10.1523/JNEUROSCI.1688-13.2013 PMC6618777

[153] S. Samejima , A. Khorasani , V. Ranganathan , et al., “Brain‐Computer‐Spinal Interface Restores Upper Limb Function After Spinal Cord Injury,” IEEE Transactions on Neural Systems and Rehabilitation Engineering: A Publication of the IEEE Engineering in Medicine and Biology Society 29 (2021): 1233–1242. 34138712 10.1109/TNSRE.2021.3090269

[154] I. Seáñez and M. Capogrosso , “Motor Improvements Enabled by Spinal Cord Stimulation Combined With Physical Training After Spinal Cord Injury: Review of Experimental Evidence in Animals and Humans,” Bioelectronic Medicine 7, no. 1 (2021): 16. 34706778 10.1186/s42234-021-00077-5 PMC8555080

[155] A. Jackson and J. B. Zimmermann , “Neural Interfaces for the Brain and Spinal Cord—Restoring Motor Function,” Nature Reviews Neurology 8, no. 12 (2012): 690–699. 23147846 10.1038/nrneurol.2012.219

[156] B. J. Edelman , J. Meng , D. Suma , et al., “Noninvasive Neuroimaging Enhances Continuous Neural Tracking for Robotic Device Control,” Science Robotics 4, no. 31 (2019): eaaw6844. 31656937 10.1126/scirobotics.aaw6844 PMC6814169

[157] N. Greiner , B. Barra , G. Schiavone , et al., “Recruitment of Upper‐Limb Motoneurons With Epidural Electrical Stimulation of the Cervical Spinal Cord,” Nature Communications 12, no. 1 (2021): 435. 10.1038/s41467-020-20703-1 PMC7815834 33469022

[158] K. Kato , Y. Nishihara , and Y. Nishimura , “Stimulus Outputs Induced by Subdural Electrodes on the Cervical Spinal Cord in Monkeys,” Journal of Neural Engineering 17, no. 1 (2020): 016044. 32023224 10.1088/1741-2552/ab63a3

[159] J. S. Calvert , P. J. Grahn , K. D. Zhao , and K. H. Lee , “Emergence of Epidural Electrical Stimulation to Facilitate Sensorimotor Network Functionality After Spinal Cord Injury,” Neuromodulation: Journal of the International Neuromodulation Society 22, no. 3 (2019): 244–252. 30840354 10.1111/ner.12938

[160] C. Moritz , E. C. Field‐Fote , C. Tefertiller , et al., “Non‐Invasive Spinal Cord Electrical Stimulation for Arm and Hand Function in Chronic Tetraplegia: A Safety and Efficacy Trial,” Nature Medicine 30, no. 5 (2024): 1276–1283. 10.1038/s41591-024-02940-9 PMC11108781 38769431

[161] Y. Sui , H. Yu , C. Zhang , Y. Chen , C. Jiang , and L. Li , “Deep Brain‐Machine Interfaces: Sensing and Modulating the Human Deep Brain,” National Science Review 9, no. 10 (2022): nwac212. 36644311 10.1093/nsr/nwac212 PMC9834907

[162] G. J. B. Elias , A. A. Namasivayam , and A. M. Lozano , “Deep Brain Stimulation for Stroke: Current Uses and Future Directions,” Brain Stimulation 11, no. 1 (2018): 3–28. 29089234 10.1016/j.brs.2017.10.005

[163] M. DeLong and T. Wichmann , “Deep Brain Stimulation for Movement and Other Neurologic Disorders,” Annals of the New York Academy of Sciences 1265 (2012): 1–8. 22823512 10.1111/j.1749-6632.2012.06608.x PMC4314938

[164] L. L. Oganesian and M. M. Shanechi , “Brain–Computer Interfaces for Neuropsychiatric Disorders,” Nature Reviews Bioengineering 2, no. 8 (2024): 653–670. 10.1038/s44222-024-00177-2 PMC12453833 40988938

[165] D. J. Lee , C. S. Lozano , R. F. Dallapiazza , and A. M. Lozano , “Current and Future Directions of Deep Brain Stimulation for Neurological and Psychiatric Disorders,” Journal of Neurosurgery 131, no. 2 (2019): 333–342. 31370011 10.3171/2019.4.JNS181761

[166] G. Kumar and C. R. Soni , “Central Post‐Stroke Pain: Current Evidence,” Journal of the Neurological Sciences 284, no. 1‐2 (2009): 10–17. 19419737 10.1016/j.jns.2009.04.030

[167] M. R. Paro , M. Dyrda , S. Ramanan , et al., “Deep Brain Stimulation for Movement Disorders After Stroke: A Systematic Review of the Literature,” Journal of Neurosurgery 138, no. 6 (2022): 1688–1701. 36308482 10.3171/2022.8.JNS221334

[168] J. C. Ho , E. M. Grigsby , A. Damiani , et al., “Potentiation of Cortico‐Spinal Output via Targeted Electrical Stimulation of the Motor Thalamus,” Nature Communications 15, no. 1 (2024): 8461. 10.1038/s41467-024-52477-1 PMC11445460 39353911

[169] S. D. Krämer , M. K. Schuhmann , J. Volkmann , and F. Fluri , “Deep Brain Stimulation in the Subthalamic Nucleus Can Improve Skilled Forelimb Movements and Retune Dynamics of Striatal Networks in a Rat Stroke Model,” International Journal of Molecular Sciences 23, no. 24 (2022): 15862. 36555504 10.3390/ijms232415862 PMC9779486

[170] J. Xu , B. Liu , S. Liu , et al., “Efficacy and Safety of Deep Brain Stimulation in Mesencephalic Locomotor Region for Motor Function in Patients With Post‐Stroke Hemiplegia: A Study Protocol for a Multi‐Center Double‐Blind Crossover Randomized Controlled Trial,” Frontiers in Neurology 15 (2024): 1355104. 39193146 10.3389/fneur.2024.1355104 PMC11347412

[171] N. Cho , J. W. Squair , V. Aureli , et al., “Hypothalamic Deep Brain Stimulation Augments Walking After Spinal Cord Injury,” Nature Medicine 30, no. 12 (2024): 3676–3686. 10.1038/s41591-024-03306-x 39623087

[172] R. N. Lemon , “Descending Pathways in Motor Control,” Annual Review of Neuroscience 31 (2008): 195–218. 10.1146/annurev.neuro.31.060407.125547 18558853

[173] S. P. Hsu , C. F. Lu , B. F. Lin , et al., “Effects of Bihemispheric Transcranial Direct Current Stimulation on Motor Recovery in Subacute Stroke Patients: A Double‐Blind, Randomized Sham‐Controlled Trial,” Journal of NeuroEngineering and Rehabilitation 20, no. 1 (2023): 27. 36849990 10.1186/s12984-023-01153-4 PMC9969953

[174] M. Coscia , M. J. Wessel , U. Chaudary , et al., “Neurotechnology‐Aided Interventions for Upper Limb Motor Rehabilitation in Severe Chronic Stroke,” Brain 142, no. 8 (2019): 2182–2197. 31257411 10.1093/brain/awz181 PMC6658861

[175] J. P. Lefaucheur , A. Aleman , C. Baeken , et al., “Evidence‐Based Guidelines on the Therapeutic Use of Repetitive Transcranial Magnetic Stimulation (rTMS): An Update (2014‐2018),” Clinical Neurophysiology: Official Journal of the International Federation of Clinical Neurophysiology 131, no. 2 (2020): 474–528. 31901449 10.1016/j.clinph.2019.11.002

[176] M. Bonizzato and M. Martinez , “An Intracortical Neuroprosthesis Immediately Alleviates Walking Deficits and Improves Recovery of Leg Control After Spinal Cord Injury,” Science Translational Medicine 13, no. 586 (2021): eabb4422. 33762436 10.1126/scitranslmed.abb4422

[177] A. Machado and K. B. Baker , “Upside Down Crossed Cerebellar Diaschisis: Proposing Chronic Stimulation of the Dentatothalamocortical Pathway for Post‐Stroke Motor Recovery,” Frontiers in Integrative Neuroscience 6 (2012): 20. 22661933 10.3389/fnint.2012.00020 PMC3357012

[178] A. G. Machado , J. Cooperrider , H. T. Furmaga , et al., “Chronic 30‐hz Deep Cerebellar Stimulation Coupled With Training Enhances Post‐Ischemia Motor Recovery and Peri‐Infarct Synaptophysin Expression in Rodents,” Neurosurgery 73, no. 2 (2013): 344–353, discussion 353. 23670034 10.1227/01.neu.0000430766.80102.ac

[179] H. J. Park , H. Furmaga , J. Cooperrider , J. T. Gale , K. B. Baker , and A. G. Machado , “Modulation of Cortical Motor Evoked Potential After Stroke During Electrical Stimulation of the Lateral Cerebellar Nucleus,” Brain Stimulation 8, no. 6 (2015): 1043–1048. 26215752 10.1016/j.brs.2015.06.020 PMC4656069

[180] J. Cooperrider , H. Furmaga , E. Plow , et al., “Chronic Deep Cerebellar Stimulation Promotes Long‐Term Potentiation, Microstructural Plasticity, and Reorganization of Perilesional Cortical Representation in a Rodent Model,” Journal of Neuroscience: The Official Journal of the Society for Neuroscience 34, no. 27 (2014): 9040–9050. 24990924 10.1523/JNEUROSCI.0953-14.2014 PMC4078081

[181] A. M. Shah , S. Ishizaka , M. Y. Cheng , et al., “Optogenetic Neuronal Stimulation of the Lateral Cerebellar Nucleus Promotes Persistent Functional Recovery After Stroke,” Scientific Reports 7 (2017): 46612. 28569261 10.1038/srep46612 PMC5451884

[182] W. J. Neumann , R. Gilron , S. Little , and G. Tinkhauser , “Adaptive Deep Brain Stimulation: From Experimental Evidence Toward Practical Implementation,” Movement Disorders 38, no. 6 (2023): 937–948. 37148553 10.1002/mds.29415

[183] S. Zanos , “Closed‐Loop Neuromodulation in Physiological and Translational Research,” Cold Spring Harbor Perspectives in Medicine 9, no. 11 (2019): a034314. 30559253 10.1101/cshperspect.a034314 PMC6824403

[184] S. L. Schmidt , A. H. Chowdhury , K. T. Mitchell , et al., “At Home Adaptive Dual Target Deep Brain Stimulation in Parkinson's Disease With Proportional Control,” Brain: A Journal of Neurology 147, no. 3 (2024): 911–922. 38128546 10.1093/brain/awad429 PMC10907084

[185] N. C. Swann , C. de Hemptinne , M. C. Thompson , et al., “Adaptive Deep Brain Stimulation for Parkinson's disease Using Motor Cortex Sensing,” Journal of Neural Engineering 15, no. 4 (2018): 046006. 29741160 10.1088/1741-2552/aabc9b PMC6021210

[186] J. A. Herron , M. C. Thompson , T. Brown , H. J. Chizeck , J. G. Ojemann , and A. L. Ko , “Cortical Brain‐Computer Interface for Closed‐Loop Deep Brain Stimulation,” IEEE Transactions on Neural Systems and Rehabilitation Engineering: A Publication of the IEEE Engineering in Medicine and Biology Society 25, no. 11 (2017): 2180–2187. 28541211 10.1109/TNSRE.2017.2705661

[187] B. Kim and C. Winstein , “Can Neurological Biomarkers of Brain Impairment be Used to Predict Poststroke Motor Recovery? A Systematic Review,” Neurorehabilitation and Neural Repair 31, no. 1 (2017): 3–24. 27503908 10.1177/1545968316662708

[188] R. Mane , E. Chew , K. S. Phua , et al., “Prognostic and Monitory EEG‐Biomarkers for BCI Upper‐Limb Stroke Rehabilitation,” IEEE Transactions on Neural Systems and Rehabilitation Engineering: A Publication of the IEEE Engineering in Medicine and Biology Society 27, no. 8 (2019): 1654–1664. 31247558 10.1109/TNSRE.2019.2924742

[189] L. A. Boyd , K. S. Hayward , N. S. Ward , et al., “Biomarkers of Stroke Recovery: Consensus‐Based Core Recommendations From the Stroke Recovery and Rehabilitation Roundtable,” International Journal of Stroke: Official Journal of the International Stroke Society 12, no. 5 (2017): 480–493. 28697711 10.1177/1747493017714176 PMC6791523

[190] W. J. Neumann , F. Staub‐Bartelt , A. Horn , et al., “Long Term Correlation of Subthalamic Beta Band Activity With Motor Impairment in Patients With Parkinson's Disease,” Clinical Neurophysiology: Official Journal of the International Federation of Clinical Neurophysiology 128, no. 11 (2017): 2286–2291. 29031219 10.1016/j.clinph.2017.08.028 PMC5779610

[191] T. Morishita , M. S. Okun , A. Burdick , C. E. Jacobson , and K. D. Foote , “Cerebral Venous Infarction: A Potentially Avoidable Complication of Deep Brain Stimulation Surgery,” Neuromodulation 16, no. 5 (2013): 407–413, discussion 413. 23738501 10.1111/ner.12052 PMC3772976

[192] T. Morishita , K. D. Foote , A. P. Burdick , et al., “Identification and Management of Deep Brain Stimulation Intra‐ and Postoperative Urgencies and Emergencies,” Parkinsonism & Related Disorders 16, no. 3 (2010): 153–162. 19896407 10.1016/j.parkreldis.2009.10.003 PMC2829374

[193] A. Gorgulho , A. A. F. De Salles , L. Frighetto , and E. Behnke , “Incidence of Hemorrhage Associated With Electrophysiological Studies Performed Using Macroelectrodes and Microelectrodes in Functional Neurosurgery,” Journal of Neurosurgery 102, no. 5 (2005): 888–896. 15926715 10.3171/jns.2005.102.5.0888

[194] D. K. Binder , G. M. Rau , and P. A. Starr , “Risk Factors for Hemorrhage During Microelectrode‐Guided Deep Brain Stimulator Implantation for Movement Disorders,” Neurosurgery 56, no. 4 (2005): 722–732, discussion 722–732. 15792511 10.1227/01.neu.0000156473.57196.7e

[195] C. Hamani , E. Richter , J. M. Schwalb , and A. M. Lozano , “Bilateral Subthalamic Nucleus Stimulation for Parkinson's Disease: A Systematic Review of the Clinical Literature,” Neurosurgery 56, no. 6 (2005): 1313–1321, discussion 1321–1324. 15918948 10.1227/01.neu.0000159714.28232.c4

[196] A. Videnovic and L. V. Metman , “Deep Brain Stimulation for Parkinson's Disease: Prevalence of Adverse Events and Need for Standardized Reporting,” Movement Disorders 23, no. 3 (2008): 343–349. 17987644 10.1002/mds.21753

[197] B. T. Himes , G. W. Mallory , A. S. Abcejo , et al., “Contemporary Analysis of the Intraoperative and Perioperative Complications of Neurosurgical Procedures Performed in the Sitting Position,” Journal of Neurosurgery 127, no. 1 (2017): 182–188. 27494821 10.3171/2016.5.JNS152328

[198] V. Krishna , F. Sammartino , and A. Rezai , “A Review of the Current Therapies, Challenges, and Future Directions of Transcranial Focused Ultrasound Technology: Advances in Diagnosis and Treatment,” JAMA Neurology 75, no. 2 (2018): 246–254. 29228074 10.1001/jamaneurol.2017.3129

[199] H. Kim , J. Kim , J. Kim , S. Oh , K. Choi , and J. Yoon , “Magnetothermal‐Based Non‐Invasive Focused Magnetic Stimulation for Functional Recovery in Chronic Stroke Treatment,” Scientific Reports 13, no. 1 (2023): 4988. 36973390 10.1038/s41598-023-31979-w PMC10042827

[200] I. R. Violante , K. Alania , A. M. Cassarà , et al., “Non‐Invasive Temporal Interference Electrical Stimulation of the human Hippocampus,” Nature Neuroscience 26, no. 11 (2023): 1994–2004. 37857775 10.1038/s41593-023-01456-8 PMC10620081

[201] M. J. Wessel , E. Beanato , T. Popa , et al., “Noninvasive Theta‐Burst Stimulation of the Human Striatum Enhances Striatal Activity and Motor Skill Learning,” Nature Neuroscience 26, no. 11 (2023): 2005–2016. 37857774 10.1038/s41593-023-01457-7 PMC10620076

[202] Z. Huang , C. C. Charalambous , M. Chen , et al., “Low Intensity Focused Ultrasound Stimulation in Stroke: A Phase I Safety & Feasibility Trial,” Brain Stimulation 18, no. 1 (2025): 179–187. 39842609 10.1016/j.brs.2025.01.015

[203] C. Yang , Y. Xu , Y. Du , et al., “Transcranial Temporal Interference Subthalamic Stimulation for Treating Motor Symptoms in Parkinson's Disease: A Pilot Study,” Brain Stimulation 17, no. 6 (2024): 1250–1252. 39486623 10.1016/j.brs.2024.10.012

[204] J. R. Wolpaw , N. Birbaumer , D. J. McFarland , G. Pfurtscheller , and T. M. Vaughan , “Brain‐Computer Interfaces for Communication and Control,” Clinical Neurophysiology 113, no. 6 (2002): 767–791. 12048038 10.1016/s1388-2457(02)00057-3

[205] G. A. Tabot , J. F. Dammann , J. A. Berg , et al., “Restoring the Sense of Touch With a Prosthetic Hand Through a Brain Interface,” Proceedings National Academy of Science USA 110, no. 45 (2013): 18279–18284. 10.1073/pnas.1221113110 PMC3831459 24127595

[206] R. P. Rao , “Towards Neural Co‐processors for the Brain: Combining Decoding and Encoding in Brain‐Computer Interfaces,” Current Opinion in Neurobiology 55 (2019): 142–151. 30954862 10.1016/j.conb.2019.03.008 PMC6860027

[207] C. Hughes , A. Herrera , R. Gaunt , and J. Collinger , “Bidirectional Brain‐Computer Interfaces,” in Handbook of Clinical Neurology (Elsevier, 2020), 163–181. 10.1016/B978-0-444-63934-9.00013-5 32164851

[208] M. A. Lebedev and M. A. L. Nicolelis , “Brain‐Machine Interfaces: From Basic Science to Neuroprostheses and Neurorehabilitation,” Physiological Reviews 97, no. 2 (2017): 767–837. 28275048 10.1152/physrev.00027.2016

[209] R. Y. Lim , M. Jiang , K. K. Ang , X. Lin , and C. Guan , “Brain‐Computer‐Brain System for Individualized Transcranial Alternating Current Stimulation With Concurrent EEG Recording: A Healthy Subject Pilot Study,” in 2024 46th Annual International Conference of the IEEE Engineering in Medicine and Biology Society (EMBC) (IEEE, 2024), 1–4, 10.1109/EMBC53108.2024.10782251. 40039276

[210] A. J. Bergquist , J. M. Clair , O. Lagerquist , C. S. Mang , Y. Okuma , and D. F. Collins , “Neuromuscular Electrical Stimulation: Implications of the Electrically Evoked Sensory Volley,” European Journal of Applied Physiology 111, no. 10 (2011): 2409–2426. 21805156 10.1007/s00421-011-2087-9

[211] N. L. Opie , S. E. John , G. S. Rind , et al., “Focal Stimulation of the Sheep Motor Cortex With a Chronically Implanted Minimally Invasive Electrode Array Mounted on an Endovascular Stent,” Nature Biomedical Engineering 2, no. 12 (2018): 907–914. 10.1038/s41551-018-0321-z 31015727

[212] C. Neudorfer , K. Bhatia , A. Boutet , et al., “Endovascular Deep Brain Stimulation: Investigating the Relationship Between Vascular Structures and Deep Brain Stimulation Targets,” Brain Stimulation 13, no. 6 (2020): 1668–1677. 33035721 10.1016/j.brs.2020.09.016

[213] V. A. Coenen , B. Sajonz , T. Prokop , et al., “The Dentato‐rubro‐thalamic Tract as the Potential Common Deep Brain Stimulation Target for Tremor of Various Origin: An Observational Case Series,” Acta Neurochirurgica 162, no. 5 (2020): 1053–1066. 31997069 10.1007/s00701-020-04248-2 PMC7156360

[214] G. Rajah , H. Saber , R. Singh , and L. Rangel‐Castilla , “Endovascular Delivery of Leads and Stentrodes and Their Applications to Deep Brain Stimulation and Neuromodulation: A Review,” Neurosurgical Focus 45, no. 2 (2018): E19. 10.3171/2018.4.FOCUS18130 30064310

[215] K. Brodmann , Vergleichende Lokalisationslehre Der Grosshirnrinde in Ihren Prinzipien Dargestellt Auf Grund Des Zellenbaues (Barth, 1909).

[216] E. Heming , A. Sanden , and Z. H. T. Kiss , “Designing a Somatosensory Neural Prosthesis: Percepts Evoked by Different Patterns of Thalamic Stimulation,” Journal of Neural Engineering 7, no. 6 (2010): 064001. 21084731 10.1088/1741-2560/7/6/064001

[217] Y. Moon , C. Yang , N. C. Veit , et al., “Noninvasive Spinal Stimulation Improves Walking in Chronic Stroke Survivors: A Proof‐of‐Concept Case Series,” BioMedical Engineering OnLine 23, no. 1 (2024): 38. 38561821 10.1186/s12938-024-01231-1 PMC10986021

[218] J. B. Zimmermann and A. Jackson , “Closed‐Loop Control of Spinal Cord Stimulation to Restore Hand Function After Paralysis,” Frontiers in Neuroscience 8 (2014): 87. 24904251 10.3389/fnins.2014.00087 PMC4032985

[219] X. L. Huang , M. Y. Wu , C. C. Wu , et al., “Neuromodulation Techniques in Poststroke Motor Impairment Recovery: Efficacy, Challenges, and Future Directions,” Tzu Chi Medical Journal 36, no. 2 (2024): 136–141. 38645790 10.4103/tcmj.tcmj_247_23 PMC11025597

[220] X. Li , K. B. Baker , and K. O'Laughlin , “Paired DBS and TMS Reveals Dentato‐Cortical Facilitation Underlying Upper Extremity Movement in Chronic Stroke Survivors,” Neurorehabilitation and Neural Repair 38, no. 2 (2024): 109–121. 38156644 10.1177/15459683231219265 PMC10922453

[221] C. Nielsen , V. Siersma , E. Ghaziani , N. Beyer , S. P. Magnusson , and C. Couppé , “Health‐Related Quality of Life and Physical Function in Individuals With parkinson's Disease After a Multidisciplinary Rehabilitation Regimen—A Prospective Cohort Feasibility Study,” International Journal of Environmental Research and Public Health 17, no. 20 (2020): 7668. 33096677 10.3390/ijerph17207668 PMC7589165

[222] E. Ortega‐Robles , R. I. Carino‐Escobar , J. Cantillo‐Negrete , and O. Arias‐Carrión , “Brain–Computer Interfaces in Parkinson's Disease Rehabilitation,” Biomimetics 10, no. 8 (2025): 488. 40862861 10.3390/biomimetics10080488 PMC12383679

[223] H. Tayebi , S. Azadnajafabad , S. F. Maroufi , et al., “Applications of Brain‐Computer Interfaces in Neurodegenerative Diseases,” Neurosurgical Review 46, no. 1 (2023): 131. 37256332 10.1007/s10143-023-02038-9

[224] E. R. Dorsey , T. Sherer , M. S. Okun , and B. R. Bloem , “The Emerging Evidence of the Parkinson Pandemic,” Journal of Parkinson's Disease 8, no. s1 (2018): S3–S8. 10.3233/JPD-181474 PMC6311367 30584159

[225] M. J. Armstrong and M. S. Okun , “Diagnosis and Treatment of Parkinson Disease: A Review,” JAMA 323, no. 6 (2020): 548–560. 32044947 10.1001/jama.2019.22360

[226] S. Marceglia , M. Guidetti , I. E. Harmsen , et al., “Deep Brain Stimulation: Is It Time to Change Gears by Closing the Loop?,” Journal of Neural Engineering 18, no. 6 (2021): 061001. 10.1088/1741-2552/ac3267 34678794

[227] M. Arlotti , M. Colombo , A. Bonfanti , et al., “A New Implantable Closed‐Loop Clinical Neural Interface: First Application in Parkinson's Disease,” Frontiers in Neuroscience 15 (2021): 763235. 34949982 10.3389/fnins.2021.763235 PMC8689059

[228] A. Schnitzler and J. Gross , “Normal and Pathological Oscillatory Communication in the Brain,” Nature Reviews Neuroscience 6, no. 4 (2005): 285–296. 15803160 10.1038/nrn1650

[229] F. Stocchi , D. Bravi , A. Emmi , and A. Antonini , “Parkinson Disease Therapy: Current Strategies and Future Research Priorities,” Nature Reviews Neuroscience 20, no. 12 (2024): 695–707. 10.1038/s41582-024-01034-x 39496848

[230] J. L. Busch , J. Kaplan , J. G. V. Habets , et al., “Single Threshold Adaptive Deep Brain Stimulation in Parkinson's Disease Depends on Parameter Selection, Movement state and Controllability of Subthalamic Beta Activity,” Brain Stimulation 17, no. 1 (2024): 125–133. 38266773 10.1016/j.brs.2024.01.007

[231] S. Little , A. Pogosyan , S. Neal , et al., “Adaptive Deep Brain Stimulation in Advanced Parkinson Disease,” Annals of Neurology 74, no. 3 (2013): 449–457. 23852650 10.1002/ana.23951 PMC3886292

[232] T. Bocci , M. Prenassi , M. Arlotti , et al., “Eight‐Hours Conventional Versus Adaptive Deep Brain Stimulation of the Subthalamic Nucleus in Parkinson's Disease,” Npj Parkinson's Disease 7, no. 1 (2021): 88. 10.1038/s41531-021-00229-z PMC8478873 34584095

[233] L. Caffi , L. M. Romito , C. Palmisano , et al., “Adaptive vs. Conventional Deep Brain Stimulation: One‐Year Subthalamic Recordings and Clinical Monitoring in a Patient With Parkinson's Disease,” Bioengineering 11, no. 10 (2024): 990. 39451366 10.3390/bioengineering11100990 PMC11504236

[234] Q. Li , K. Wang , J. Li , et al., “Adaptive vs Conventional Deep Brain Stimulation of the Subthalamic Nucleus for Treatment of Parkinson's Disease: A Multicenter Retrospective Study,” Neuromodulation (2025): S1094–7159(25)00186–00182. 10.1016/j.neurom.2025.04.011 40488687

[235] Q. An , Z. Yin , R. Ma , et al., “Adaptive Deep Brain Stimulation for Parkinson's Disease: Looking Back at the Past Decade on Motor Outcomes,” Journal of Neurology 270, no. 3 (2023): 1371–1387. 36471098 10.1007/s00415-022-11495-z

[236] G. Lio , S. Thobois , B. Ballanger , B. Lau , and P. Boulinguez , “Removing Deep Brain Stimulation Artifacts From the Electroencephalogram: Issues, Recommendations and an Open‐Source Toolbox,” Clinical Neurophysiology: Official Journal of the International Federation of Clinical Neurophysiology 129, no. 10 (2018): 2170–2185. 30144660 10.1016/j.clinph.2018.07.023

[237] J. Ansó , M. Benjaber , B. Parks , et al., “Concurrent Stimulation and Sensing in Bi‐Directional Brain Interfaces: A Multi‐Site Translational Experience,” Journal of Neural Engineering 19, no. 2 (2022): 026025. 10.1088/1741-2552/ac59a3 PMC9095704 35234664

[238] N. C. Swann , C. de Hemptinne , S. Miocinovic , et al., “Gamma Oscillations in the Hyperkinetic State Detected With Chronic Human Brain Recordings in Parkinson's Disease,” Journal of Neuroscience: Official Journal of the Society for Neuroscience 36, no. 24 (2016): 6445–6458. 10.1523/JNEUROSCI.1128-16.2016 PMC5015781 27307233

[239] A. A. Kühn , D. Williams , A. Kupsch , et al., “Event‐Related Beta Desynchronization in Human Subthalamic Nucleus Correlates With Motor Performance,” Brain: A Journal of Neurology 127, no. pt. 4 (2004): 735–746. 14960502 10.1093/brain/awh106

[240] N. E. Crone , D. L. Miglioretti , B. Gordon , and R. P. Lesser , “Functional Mapping of Human Sensorimotor Cortex With Electrocorticographic Spectral Analysis. II. Event‐Related Synchronization in the Gamma Band,” Brain: A Journal of Neurology 121, no. pt. 12 (1998): 2301–2315. 9874481 10.1093/brain/121.12.2301

[241] K. J. Miller , E. C. Leuthardt , G. Schalk , et al., “Spectral Changes in Cortical Surface Potentials During Motor Movement,” Journal of Neurology: Official Journal of Social Neuroscience 27, no. 9 (2007): 2424–2432. 10.1523/JNEUROSCI.3886-06.2007 PMC6673496 17329441

[242] M. M. Turconi , S. Mezzarobba , G. Franco , et al., “BCI‐Based Neuro‐Rehabilitation Treatment for Parkinson's Disease: Cases Report,” TSPC2014.

[243] V. Lavermicocca , A. R. Dellomonaco , A. Tedesco , M. Notarnicola , R. Di Fede , and P. P. Battaglini , “[Neurofeedback in Parkinson's Disease: Technologies in Speech and Language Therapy],” Recenti Progressi in Medicina 109, no. 2 (2018): 130–132. 29493639 10.1701/2865.28908

[244] T. S. de Aguiar Pettenuzzo , K. M. Estivalet , B. M. Strey , and F. Cechetti , “Brain‐Computer Interface in Cognitive Rehabilitation for Individuals With Parkinson's Disease and Improvement in Occupational Performance: A Pilot Study,” Brazilian Journal of Physical Therapy 29 (2025): 101433.

[245] L. Subramanian , J. V. Hindle , S. Johnston , et al., “Real‐Time Functional Magnetic Resonance Imaging Neurofeedback for Treatment of Parkinson's Disease,” Journal of Neurology: Official Journal of Social Neuroscience 31, no. 45 (2011): 16309–16317. 10.1523/JNEUROSCI.3498-11.2011 PMC6633236 22072682

[246] K. Buyukturkoglu , M. Rana , and S. Ruiz , “Volitional Regulation of the Supplementary Motor Area With fMRI‐BCI Neurofeedback in Parkinson's Disease: A Pilot Study,” in 2013 6th International IEEE/EMBS Conference on Neural Engineering (NER) (IEEE, 2013), 677–681, 10.1109/NER.2013.6696025.

[247] M. Lamoš , M. Bočková , F. Missey , et al., “Noninvasive Temporal Interference Stimulation of the Subthalamic Nucleus in Parkinson's Disease Reduces Beta Activity,” Movement Disorders 40, no. 6 (2025): 1051–1060. 40202094 10.1002/mds.30134 PMC12160966

[248] G. Darmani , H. Ramezanpour , C. Sarica , et al., “Individualized Non‐Invasive Deep Brain Stimulation of the Basal Ganglia Using Transcranial Ultrasound Stimulation,” Nature Communications 16, no. 1 (2025): 2693. 10.1038/s41467-025-57883-7 PMC11923056 40108143

[249] G. Xu , T. Ren , Y. Chen , and W. Che , “A One‐Dimensional CNN‐LSTM Model for Epileptic Seizure Recognition Using EEG Signal Analysis,” Frontiers in Neuroscience 14 (2020): 578126. 33390878 10.3389/fnins.2020.578126 PMC7772824

[250] R. D. Thijs , R. Surges , T. J. O'Brien , and J. W. Sander , “Epilepsy in Adults,” Lancet 393, no. 10172 (2019): 689–701. 30686584 10.1016/S0140-6736(18)32596-0

[251] X. Y. Liu , W. L. Wang , M. Liu , et al., “Recent Applications of EEG‐Based Brain‐Computer‐Interface in the Medical Field,” Military Medical Research 12 (2025): 14. 40128831 10.1186/s40779-025-00598-z PMC11931852

[252] L. Kuhlmann , K. Lehnertz , M. P. Richardson , B. Schelter , and H. P. Zaveri , “Seizure Prediction—Ready for a New Era,” Nature Reviews Neurology 14, no. 10 (2018): 618–630. 30131521 10.1038/s41582-018-0055-2

[253] C. E. Elger and C. Hoppe , “Diagnostic Challenges in Epilepsy: Seizure Under‐Reporting and Seizure Detection,” Lancet Neurology 17, no. 3 (2018): 279–288. 29452687 10.1016/S1474-4422(18)30038-3

[254] T. Proix , W. Truccolo , M. G. Leguia , et al., “Forecasting Seizure Risk in Adults With Focal Epilepsy: A Development and Validation Study,” Lancet Neurology 20, no. 2 (2021): 127–135. 33341149 10.1016/S1474-4422(20)30396-3 PMC7968722

[255] J. L. McKee , M. C. Kaufman , A. K. Gonzalez , et al., “Leveraging Electronic Medical Record‐Embedded Standardised Electroencephalogram Reporting to Develop Neonatal Seizure Prediction Models: A Retrospective Cohort Study,” Lancet, Digit Health 5, no. 4 (2023): e217–e226. 36963911 10.1016/S2589-7500(23)00004-3 PMC10065843

[256] H. S. Nogay and H. Adeli , “Detection of Epileptic Seizure Using Pretrained Deep Convolutional Neural Network and Transfer Learning,” European Neurology 83, no. 6 (2020): 602–614. 33423031 10.1159/000512985

[257] O. Faust , U. R. Acharya , H. Adeli , and A. Adeli , “Wavelet‐Based EEG Processing for Computer‐Aided Seizure Detection and Epilepsy Diagnosis,” Seizure: The Journal of the British Epilepsy Association 26 (2015): 56–64. 10.1016/j.seizure.2015.01.012 25799903

[258] Y. Tang and D. Durand , “A Tunable Support Vector Machine Assembly Classifier for Epileptic Seizure Detection,” Expert Systems with Applications 39, no. 4 (2012): 3925–3938. 22563146 10.1016/j.eswa.2011.08.088 PMC3341176

[259] X. Wei , L. Zhou , Z. Chen , L. Zhang , and Y. Zhou , “Automatic Seizure Detection Using Three‐Dimensional CNN Based on Multi‐Channel EEG,” BMC Medical Informatics and Decision Making 18, no. S5 (2018): 111. 30526571 10.1186/s12911-018-0693-8 PMC6284363

[260] I. Ahmad , X. Wang , D. Javeed , P. Kumar , O. W. Samuel , and S. Chen , “A Hybrid Deep Learning Approach for Epileptic Seizure Detection in EEG Signals,” IEEE Journal of Biomedical and Health Informatics 30, no. 2 (2025): 1019–1029. 10.1109/JBHI.2023.3265983 37037252

[261] A. Burrello , L. Cavigelli , K. Schindler , L. Benini , and A. Rahimi , “Laelaps: An Energy‐Efficient Seizure Detection Algorithm From Long‐Term Human iEEG Recordings Without False Alarms,” in 2019 Design, Automation & Test in Europe Conference & Exhibition (DATE) (IEEE, 2019), 752–757, 10.23919/DATE.2019.8715186.

[262] M. C. Tjepkema‐Cloostermans , R. C. V. de Carvalho , and M. van Putten , “Deep Learning for Detection of Focal Epileptiform Discharges From Scalp EEG Recordings,” Clinical Neurophysiology: Official Publication of the International Federation of Clinical Neurophysiology 129, no. 10 (2018): 2191–2196. 10.1016/j.clinph.2018.06.024 30025804

[263] Y. Zhong , Y. Wang , Z. He , et al., “Closed‐Loop Wearable Ultrasound Deep Brain Stimulation System Based on EEG in Mice,” Journal of Neural Engineering 18, no. 4 (2021): 0460e8. 10.1088/1741-2552/ac1d5c 34388739

[264] W. P. Welch , B. Sitwat , and Y. Sogawa , “Use of Vagus Nerve Stimulator on Children With Primary Generalized Epilepsy,” Journal of Child Neurology 33, no. 7 (2018): 449–452. 29651891 10.1177/0883073818766599

[265] Y. T. Fang , Y. T. Lin , W. L. Tseng , et al., “Neuroimmunomodulation of Vagus Nerve Stimulation and the Therapeutic Implications,” Frontiers in Aging Neuroscience 15 (2023): 1173987. 37484689 10.3389/fnagi.2023.1173987 PMC10358778

[266] L. D. Hachem , S. M. Wong , and G. M. Ibrahim , “The Vagus Afferent Network: Emerging Role in Translational Connectomics,” Neurosurgical Focus 45, no. 3 (2018): E2. 10.3171/2018.6.FOCUS18216 30173606

[267] R. S. Fisher , P. Afra , M. Macken , et al., “Automatic Vagus Nerve Stimulation Triggered by Ictal Tachycardia: Clinical Outcomes and Device Performance–The U.S. E‐37 Trial,” Neuromodulation: Journal of International Neuromodulation Society 19, no. 2 (2016): 188–195. 10.1111/ner.12376 PMC5064739 26663671

[268] P. Boon , K. Vonck , K. van Rijckevorsel , et al., “A Prospective, Multicenter Study of Cardiac‐Based Seizure Detection to Activate Vagus Nerve Stimulation,” Seizure: The Journal of the British Epilepsy Association 32 (2015): 52–61. 10.1016/j.seizure.2015.08.011 26552564

[269] C. M. DeGiorgio , S. C. Schachter , A. Handforth , et al., “Prospective Long‐Term Study of Vagus Nerve Stimulation for the Treatment of Refractory Seizures,” Epilepsia 41, no. 9 (2000): 1195–1200. 10999559 10.1111/j.1528-1157.2000.tb00325.x

[270] N. M. Warsi , H. Yan , H. Suresh , et al., “The Anterior and Centromedian Thalamus: Anatomy, Function, and Dysfunction in Epilepsy,” Epilepsy Research 182 (2022): 106913. 35395570 10.1016/j.eplepsyres.2022.106913

[271] W. Grodd , V. J. Kumar , A. Schüz , T. Lindig , and K. Scheffler , “The Anterior and Medial Thalamic Nuclei and the Human Limbic System: Tracing the Structural Connectivity Using Diffusion‐Weighted Imaging,” Scientific Reports 10, no. 1 (2020): 10957. 32616764 10.1038/s41598-020-67770-4 PMC7331724

[272] J. P. Aggleton , S. M. O'Mara , S. D. Vann , N. F. Wright , M. Tsanov , and J. T. Erichsen , “Hippocampal‐Anterior Thalamic Pathways for Memory: Uncovering a Network of Direct and Indirect Actions,” European Journal of Neuroscience 31, no. 12 (2010): 2292–2307. 20550571 10.1111/j.1460-9568.2010.07251.x PMC2936113

[273] T. A. M. Bouwens van der Vlis , O. Schijns , F. Schaper , et al., “Deep Brain Stimulation of the Anterior Nucleus of the Thalamus for Drug‐Resistant Epilepsy,” Neurosurgical Review 42, no. 2 (2019): 287–296. 29306976 10.1007/s10143-017-0941-x PMC6502776

[274] V. Salanova , T. Witt , R. Worth , et al., “Long‐Term Efficacy and Safety of Thalamic Stimulation for Drug‐Resistant Partial Epilepsy,” Neurology 84, no. 10 (2015): 1017–1025. 25663221 10.1212/WNL.0000000000001334 PMC4352097

[275] A. Ilyas , D. Pizarro , A. K. Romeo , K. O. Riley , and S. Pati , “The Centromedian Nucleus: Anatomy, Physiology, and Clinical Implications,” Journal of Clinical Neuroscience: Official Journal of the Neurosurgical Society of Australasia 63 (2019): 1–7. 30827880 10.1016/j.jocn.2019.01.050

[276] R. S. Fisher , “Deep Brain Stimulation of Thalamus for Epilepsy,” Neurobiology of Disease 179 (2023): 106045. 36809846 10.1016/j.nbd.2023.106045

[277] R. S. Fisher , S. Uematsu , G. L. Krauss , et al., “Placebo‐Controlled Pilot Study of Centromedian Thalamic Stimulation in Treatment of Intractable Seizures,” Epilepsia 33, no. 5 (1992): 841–851. 1396427 10.1111/j.1528-1157.1992.tb02192.x

[278] F. Velasco , M. Velasco , F. Jiménez , et al., “Predictors in the Treatment of Difficult‐to‐Control Seizures by Electrical Stimulation of the Centromedian Thalamic Nucleus,” Neurosurgery 47, no. 2 (2000): 295–304, discussion 304–305. 10942002 10.1097/00006123-200008000-00007

[279] S. Agashe , D. Burkholder , K. Starnes , et al., “Centromedian Nucleus of the Thalamus Deep Brain Stimulation for Genetic Generalized Epilepsy: A Case Report and Review of Literature,” Frontiers in Human Neuroscience 16 (2022): 858413. 35669200 10.3389/fnhum.2022.858413 PMC9164300

[280] M. Ravan , S. Sabesan , and O. D'Cruz , “On Quantitative Biomarkers of VNS Therapy Using EEG and ECG Signals,” IEEE Transactions on Biomedical Engineering 64, no. 2 (2017): 419–428. 28113195 10.1109/TBME.2016.2554559

[281] E. Krook‐Magnuson , J. N. Gelinas , I. Soltesz , and G. Buzsáki , “Neuroelectronics and Biooptics: Closed‐Loop Technologies in Neurological Disorders,” JAMA Neurology 72, no. 7 (2015): 823–829. 25961887 10.1001/jamaneurol.2015.0608 PMC4501886

[282] E. Krook‐Magnuson , C. Armstrong , M. Oijala , and I. Soltesz , “On‐Demand Optogenetic Control of Spontaneous Seizures in Temporal Lobe Epilepsy,” Nature Communications 4 (2013): 1376. 10.1038/ncomms2376 PMC3562457 23340416

[283] M. J. Morrell , “Responsive Cortical Stimulation for the Treatment of Medically Intractable Partial Epilepsy,” Neurology 77, no. 13 (2011): 1295–1304. 21917777 10.1212/WNL.0b013e3182302056

[284] G. M. Winston , S. Guadix , M. T. Lavieri , et al., “Closed‐Loop Vagal Nerve Stimulation for Intractable Epilepsy: A Single‐Center Experience,” Seizure: The Journal of the British Epilepsy Association 88 (2021): 95–101. 10.1016/j.seizure.2021.03.030 33839564

[285] D. N. Anderson , C. M. Charlebois , E. H. Smith , et al., “Closed‐Loop Stimulation in Periods With Less Epileptiform Activity Drives Improved Epilepsy Outcomes,” Brain: A Journal Of Neurology 147, no. 2 (2024): 521–531. 37796038 10.1093/brain/awad343 PMC10834245

[286] H. C. Skrehot , D. J. Englot , and Z. Haneef , “Neuro‐Stimulation in Focal Epilepsy: A Systematic Review and Meta‐Analysis,” Epilepsy & Behavior: E&B 142 (2023): 109182. 10.1016/j.yebeh.2023.109182 36972642

[287] Z. Haneef and H. C. Skrehot , “Neurostimulation in Generalized Epilepsy: A Systematic Review and Meta‐Analysis,” Epilepsia 64, no. 4 (2023): 811–820. 36727550 10.1111/epi.17524

[288] Z. Lin , L. Meng , J. Zou , et al., “Non‐Invasive Ultrasonic Neuromodulation of Neuronal Excitability for Treatment of Epilepsy,” Theranostics 10, no. 12 (2020): 5514–5526. 32373225 10.7150/thno.40520 PMC7196311

[289] J. Zou , S. Yi , L. Niu , et al., “Neuroprotective Effect of Ultrasound Neuromodulation on Kainic Acid‐Induced Epilepsy in Mice,” IEEE Transactions on Ultrasonics, Ferroelectrics and Frequency Control 68, no. 9 (2021): 3006–3016. 33979280 10.1109/TUFFC.2021.3079628

[290] J. Zou , L. Meng , Z. Lin , et al., “Ultrasound Neuromodulation Inhibits Seizures in Acute Epileptic Monkeys,” Iscience 23, no. 5 (2020): 101066. 32361593 10.1016/j.isci.2020.101066 PMC7200788

[291] C. C. Lee , C. C. Chou , F. J. Hsiao , et al., “Pilot Study of Focused Ultrasound for Drug‐Resistant Epilepsy,” Epilepsia 63, no. 1 (2022): 162–175. 34729772 10.1111/epi.17105 PMC9297900

[292] S. G. Chen , C. H. Tsai , C. J. Lin , et al., “Transcranial Focused Ultrasound Pulsation Suppresses Pentylenetetrazol Induced Epilepsy in Vivo,” Brain Stimulation 13, no. 1 (2020): 35–46. 31575487 10.1016/j.brs.2019.09.011

[293] J. Kubanek , J. Brown , P. Ye , K. B. Pauly , T. Moore , and W. Newsome , “Remote, Brain Region‐Specific Control of Choice Behavior With Ultrasonic Waves,” Science Advances 6, no. 21 (2020): eaaz4193. 32671207 10.1126/sciadv.aaz4193 PMC7314556

[294] T. Choi , M. Koo , J. Joo , T. Kim , Y. M. Shon , and J. Park , “Bidirectional Neuronal Control of Epileptiform Activity by Repetitive Transcranial Focused Ultrasound Stimulations,” Advanced Science 11, no. 2 (2024): e2302404. 37997163 10.1002/advs.202302404 PMC10787102

[295] J. M. Stern , N. M. Spivak , S. A. Becerra , et al., “Safety of Focused Ultrasound Neuromodulation in Humans With Temporal Lobe Epilepsy,” Brain Stimulation 14, no. 4 (2021): 1022–1031. 34198105 10.1016/j.brs.2021.06.003

[296] H. Yang , Y. Yuan , X. Wang , and X. Li , “Closed‐Loop Transcranial Ultrasound Stimulation for Real‐Time Non‐Invasive Neuromodulation in Vivo,” Frontiers in Neuroscience 14 (2020): 445. 32477055 10.3389/fnins.2020.00445 PMC7235408

[297] J. Zou , H. Chen , X. Chen , et al., “Noninvasive Closed‐Loop Acoustic Brain‐Computer Interface for Seizure Control,” Theranostics 14, no. 15 (2024): 5965–5981. 39346532 10.7150/thno.99820 PMC11426232

[298] L. Peng , Z. Zhang , Q. Li , Z. Song , C. Yan , and H. Ling , “Unveiling the Multifaceted Pathogenesis and Therapeutic Drugs of Alzheimer's Disease: A Comprehensive Review,” Heliyon 10, no. 20 (2024): e39217. 39629139 10.1016/j.heliyon.2024.e39217 PMC11612466

[299] J. J. Jalbert , L. A. Daiello , and K. L. Lapane , “Dementia of the Alzheimer Type,” Epidemiologic Reviews 30 (2008): 15–34. 18635578 10.1093/epirev/mxn008

[300] A. D. International and M. University , “World Alzheimer Report 2021: Journey Through the Diagnosis of Dementia,” published September 21, 2021, https://www.alzint.org/resource/world‐alzheimer‐report‐2021/.

[301] B. Dubois , H. H. Feldman , C. Jacova , et al., “Advancing Research Diagnostic Criteria for Alzheimer's Disease: The IWG‐2 Criteria,” Lancet Neurology 13, no. 6 (2014): 614–629. 24849862 10.1016/S1474-4422(14)70090-0

[302] J. Jeong , “EEG Dynamics in Patients With Alzheimer's Disease,” Clinical Neurophysiology: Official Journal of the International Federation of Clinical Neurophysiology 115, no. 7 (2004): 1490–1505. 15203050 10.1016/j.clinph.2004.01.001

[303] C. Micanovic and S. Pal , “The Diagnostic Utility of EEG in Early‐Onset Dementia: A Systematic Review of the Literature With Narrative Analysis,” Journal of Neural Transmission 121, no. 1 (2014): 59–69. 23900731 10.1007/s00702-013-1070-5

[304] A. Fukushima , R. Morooka , H. Tanaka , H. Kentaro , A. Tugawa , and H. Hanyu , “Classification of Dementia Type Using the Brain‐Computer Interface,” Artificial Life and Robotics 26, no. 2 (2021): 216–221.

[305] S. H. Hojjati , A. Ebrahimzadeh , A. Khazaee , A. Babajani‐Feremi , and Alzheimer's Disease Neuroimaging Initiative , “Predicting Conversion From MCI to AD by Integrating Rs‐fMRI and Structural MRI,” Computers in Biology and Medicine 102 (2018): 30–39. 30245275 10.1016/j.compbiomed.2018.09.004

[306] C. Ieracitano , N. Mammone , A. Bramanti , A. Hussain , and F. C. Morabito , “A Convolutional Neural Network Approach for Classification of Dementia Stages Based on 2D‐Spectral Representation of EEG Recordings,” Neurocomputing 323 (2019): 96–107.

[307] H. Li , X. Yang , and L. Gong , “Functional Near‐Infrared Spectroscopy for Identifying Mild Cognitive Impairment and Alzheimer's Disease: A Systematic Review,” Frontiers in Neurology 16 (2025): 1578375. 40948656 10.3389/fneur.2025.1578375 PMC12425739

[308] A. M. Chiarelli , D. Perpetuini , P. Croce , et al., “Evidence of Neurovascular Un‐Coupling in Mild Alzheimer's Disease Through Multimodal EEG‐fNIRS and Multivariate Analysis of Resting‐State Data,” Biomedicines 9, no. 4 (2021): 337. 33810484 10.3390/biomedicines9040337 PMC8066873

[309] N. Sefati , T. Esmaeilpour , V. Salari , et al., “Monitoring Alzheimer's Disease via Ultraweak Photon Emission,” Iscience 27, no. 1 (2024): 108744. 38235338 10.1016/j.isci.2023.108744 PMC10792242

[310] D. Galvin‐McLaughlin , D. Klee , and T. Memmott , “Methodology and Preliminary Data on Feasibility of a Neurofeedback Protocol to Improve Visual Attention to Letters in Mild Alzheimer's Disease,” Contemporary Clinical Trials Communications 28 (2022): 100950. 35754975 10.1016/j.conctc.2022.100950 PMC9228283

[311] Y. Lin , I. W. Shu , and F. Singh , “Frontal Gamma as a Marker of Effective Training During Neurofeedback to Improve Memory in Patients With Mild Cognitive Impairment,” in 2023 11th International IEEE/EMBS Conference on Neural Engineering (NER) (IEEE, 2023), 1–4, 10.1109/NER52421.2023.10123807.

[312] I. Lanni , G. Chiacchierini , C. Papagno , V. Santangelo , and P. Campolongo , “Treating Alzheimer's Disease With Brain Stimulation: From Preclinical Models to Non‐Invasive Stimulation in Humans,” Neuroscience and Biobehavioral Reviews 165 (2024): 105831. 39074672 10.1016/j.neubiorev.2024.105831

[313] J. J. Im , H. Jeong , M. Bikson , et al., “Effects of 6‐Month At‐Home Transcranial Direct Current Stimulation on Cognition and Cerebral Glucose Metabolism in Alzheimer's Disease,” Brain Stimulation 12, no. 5 (2019): 1222–1228. 31196835 10.1016/j.brs.2019.06.003 PMC6703942

[314] W. Jiang , Z. Wu , L. Wen , et al., “The Efficacy of High‐ or Low‐Frequency Transcranial Magnetic Stimulation in Alzheimer's Disease Patients With Behavioral and Psychological Symptoms of Dementia,” Advances in Therapy 39, no. 1 (2022): 286–295. 34716559 10.1007/s12325-021-01964-8

[315] F. Zhang , Y. Meng , and W. Zhang , “Deep Brain Stimulation for the Treatment of Alzheimer's Disease: A Safer and More Effective Strategy,” Neural Regeneration Research 21, no. 5 (2026): 1899–1909. 40536997 10.4103/NRR.NRR-D-24-01088 PMC12694621

[316] J. Germann , R. S. C. Amaral , J. Tomaszczyk , et al., “Biomarker Changes Associated With fornix Deep Brain Stimulation in Alzheimer's Disease,” Alzheimer's Dement: Journal of Alzheimer's Association 21, no. 6 (2025): e70394. 10.1002/alz.70394 PMC12198481 40566800

[317] C. J. Swanson , Y. Zhang , S. Dhadda , et al., “A Randomized, Double‐Blind, Phase 2b Proof‐of‐Concept Clinical Trial in Early Alzheimer's Disease With Lecanemab, an Anti‐aβ Protofibril Antibody,” Alzheimer's Research & Therapy 13, no. 1 (2021): 80. 10.1186/s13195-021-00813-8 PMC8053280 33865446

[318] J. Sevigny , P. Chiao , T. Bussière , et al., “The Antibody Aducanumab Reduces Aβ Plaques in Alzheimer's Disease,” Nature 537, no. 7618 (2016): 50–56. 27582220 10.1038/nature19323

[319] C. W. Howard , M. Reich , L. Luo , et al., “Cognitive Outcomes of Deep Brain Stimulation Depend on Age and Hippocampal Connectivity in Parkinson's and Alzheimer's Disease,” Alzheimer's Dement 21, no. 8 (2025): e70498. 40838518 10.1002/alz.70498 PMC12368798

[320] D. A. Malone , D. D. Dougherty , A. R. Rezai , et al., “Deep Brain Stimulation of the Ventral Capsule/Ventral Striatum for Treatment‐Resistant Depression,” Biological Psychiatry 65, no. 4 (2009): 267–275. 18842257 10.1016/j.biopsych.2008.08.029 PMC3486635

[321] I. O. Bergfeld , M. Mantione , M. L. C. Hoogendoorn , et al., “Deep Brain Stimulation of the Ventral Anterior Limb of the Internal Capsule for Treatment‐Resistant Depression: A Randomized Clinical Trial,” JAMA Psychiatry 73, no. 5 (2016): 456–464. 27049915 10.1001/jamapsychiatry.2016.0152

[322] P. Riva‐Posse , K. S. Choi , P. E. Holtzheimer , et al., “A Connectomic Approach for Subcallosal Cingulate Deep Brain Stimulation Surgery: Prospective Targeting in Treatment‐Resistant Depression,” Molecular Psychiatry 23, no. 4 (2018): 843–849. 28397839 10.1038/mp.2017.59 PMC5636645

[323] A. M. Lozano , P. Giacobbe , C. Hamani , et al., “A Multicenter Pilot Study of Subcallosal Cingulate Area Deep Brain Stimulation for Treatment‐Resistant Depression,” Journal of Neurosurgery 116, no. 2 (2012): 315–322. 22098195 10.3171/2011.10.JNS102122

[324] D. D. Dougherty , A. R. Rezai , L. L. Carpenter , et al., “A Randomized Sham‐Controlled Trial of Deep Brain Stimulation of the Ventral Capsule/Ventral Striatum for Chronic Treatment‐Resistant Depression,” Biological Psychiatry 78, no. 4 (2015): 240–248. 25726497 10.1016/j.biopsych.2014.11.023

[325] P. E. Holtzheimer , M. M. Husain , S. H. Lisanby , et al., “Subcallosal Cingulate Deep Brain Stimulation for Treatment‐Resistant Depression: A Multisite, Randomised, Sham‐Controlled Trial,” Lancet, Psychiatry 4, no. 11 (2017): 839–849. 28988904 10.1016/S2215-0366(17)30371-1

[326] A. S. Widge , “Closed‐Loop Deep Brain Stimulation for Psychiatric Disorders,” Harvard Review of Psychiatry 31, no. 3 (2023): 162–171. 37171475 10.1097/HRP.0000000000000367 PMC10188203

[327] K. W. Scangos , G. S. Makhoul , L. P. Sugrue , E. F. Chang , and A. D. Krystal , “State‐Dependent Responses to Intracranial Brain Stimulation in a Patient With Depression,” Nature Medicine 27, no. 2 (2021): 229–231. 10.1038/s41591-020-01175-8 PMC8284979 33462446

[328] K. W. Scangos , A. N. Khambhati , P. M. Daly , et al., “Closed‐Loop Neuromodulation in an Individual With Treatment‐Resistant Depression,” Nature Medicine 27, no. 10 (2021): 1696–1700. 10.1038/s41591-021-01480-w PMC11219029 34608328

[329] S. A. Sheth , K. R. Bijanki , B. Metzger , et al., “Deep Brain Stimulation for Depression Informed by Intracranial Recordings,” Biological Psychiatry 92, no. 3 (2022): 246–251. 35063186 10.1016/j.biopsych.2021.11.007 PMC9124238

[330] A. A. Fingelkurts and A. A. Fingelkurts , “Altered Structure of Dynamic Electroencephalogram Oscillatory Pattern in Major Depression,” Biological Psychiatry 77, no. 12 (2015): 1050–1060. 25662102 10.1016/j.biopsych.2014.12.011

[331] T. Schwippel , F. Pupillo , Z. Feldman , et al., “Closed‐Loop Transcranial Alternating Current Stimulation for the Treatment of Major Depressive Disorder: An Open‐Label Pilot Study,” American Journal of Psychiatry 181, no. 9 (2024): 842–845. 39108159 10.1176/appi.ajp.20230838

[332] M. Orban , M. Elsamanty , K. Guo , S. Zhang , and H. Yang , “A Review of Brain Activity and EEG‐Based Brain–Computer Interfaces for Rehabilitation Application,” Bioengineering 9, no. 12 (2022): 768. 36550974 10.3390/bioengineering9120768 PMC9774292

[333] P. D. Ganzer , S. C. Colachis , M. A. Schwemmer , et al., “Restoring the Sense of Touch Using a Sensorimotor Demultiplexing Neural Interface,” Cell 181, no. 4 (2020): 763–773.e12. 32330415 10.1016/j.cell.2020.03.054

[334] S. L. Metzger , K. T. Littlejohn , A. B. Silva , et al., “A High‐Performance Neuroprosthesis for Speech Decoding and Avatar Control,” Nature 620, no. 7976 (2023): 1037–1046. 37612505 10.1038/s41586-023-06443-4 PMC10826467

[335] J. D. Sitt , J. R. King , and I. El Karoui , “Large Scale Screening of Neural Signatures of Consciousness in Patients in a Vegetative or Minimally Conscious State,” Brain: A Journal of Neurology 137, no. pt. 8 (2014): 2258–2270. 24919971 10.1093/brain/awu141 PMC4610185

[336] Q. He , J. He , Y. Yang , and J. Zhao , “Brain‐Computer Interfaces in Disorders of Consciousness,” Neuroscience Bulletin 39, no. 2 (2023): 348–352. 35941403 10.1007/s12264-022-00920-y PMC9905465

[337] N. Qin , Q. Cao , F. Li , W. Wang , X. Peng , and L. Wang , “A Nomogram Based on Quantitative EEG to Predict the Prognosis of Nontraumatic Coma Patients in the Neuro‐Intensive Care Unit,” Intensive & Critical Care Nursing 83 (2024): 103618. 38171953 10.1016/j.iccn.2023.103618

[338] J. Pan , Q. Xie , Y. He , et al., “Detecting Awareness in Patients With Disorders of Consciousness Using a Hybrid Brain‐Computer Interface,” Journal of Neural Engineering 11, no. 5 (2014): 56007. 10.1088/1741-2560/11/5/056007 25082743

[339] Y. H. Nho , C. E. Rolle , U. Topalovic , et al., “Responsive Deep Brain Stimulation Guided by Ventral Striatal Electrophysiology of Obsession Durably Ameliorates Compulsion,” Neuron 112, no. 1 (2024): 73–83.e4. 37865084 10.1016/j.neuron.2023.09.034 PMC10841397

[340] R. S. Shivacharan , C. E. Rolle , D. A. N. Barbosa , et al., “Pilot Study of Responsive Nucleus Accumbens Deep Brain Stimulation for Loss‐of‐Control Eating,” Nature Medicine 28, no. 9 (2022): 1791–1796. 10.1038/s41591-022-01941-w PMC9499853 36038628

[341] J. L. Gill , J. A. Schneiders , M. Stangl , et al., “A Pilot Study of Closed‐Loop Neuromodulation for Treatment‐Resistant Post‐Traumatic Stress Disorder,” Nature Communications 14, no. 1 (2023): 2997. 10.1038/s41467-023-38712-1 PMC10209131 37225710

[342] E. Waisberg , J. Ong , and A. G. Lee , “Ethical Considerations of Neuralink and Brain‐Computer Interfaces,” Annals of Biomedical Engineering 52, no. 8 (2024): 1937–1939. 38602573 10.1007/s10439-024-03511-2

[343] E. C. Gordon and A. K. Seth , “Ethical Considerations for the Use of Brain–Computer Interfaces for Cognitive Enhancement,” PLoS Biology 22, no. 10 (2024): e3002899. 39466848 10.1371/journal.pbio.3002899 PMC11542783

[344] E. Klein , T. Brown , M. Sample , A. R. Truitt , and S. Goering , “Engineering the Brain: Ethical Issues and the Introduction of Neural Devices,” Hastings Center Report 45, no. 6 (2015): 26–35. 10.1002/hast.515 26556144

[345] S. Burwell , M. Sample , and E. Racine , “Ethical Aspects of Brain Computer Interfaces: A Scoping Review,” BMC Medical Ethics 18 (2017): 60. 29121942 10.1186/s12910-017-0220-y PMC5680604

[346] W. Glannon , “Neuromodulation, Agency and Autonomy,” Brain Topography 27, no. 1 (2014): 46–54. 23322211 10.1007/s10548-012-0269-3

[347] A. Fenton and S. Alpert , “Extending Our View on Using BCIs for Locked‐in Syndrome,” Neuroethics 1, no. 2 (2008): 119–132.

[348] C. Gerardi and C. Xinaris , “Beyond Human Limits: The Ethical, Social, and Regulatory Implications of Human Enhancement,” Frontiers in Medicine 12 (2025): 1595213. 40703282 10.3389/fmed.2025.1595213 PMC12283604

[349] P. Jaszczuk , D. Bratelj , C. Capone , et al., “Advances in Neuromodulation and Digital Brain‐Spinal Cord Interfaces for Spinal Cord Injury,” International Journal of Molecular Sciences 26, no. 13 (2025): 6021. 40649800 10.3390/ijms26136021 PMC12249536

